# One-Pot Silylation–Amination Synthesis of 3‑*N*‑Substituted Benzo[*d*]isothiazole 1,1-Dioxides

**DOI:** 10.1021/acsomega.6c03641

**Published:** 2026-07-06

**Authors:** Guilherme Arraché Gonçalves, Lídia Klatt Oliveira, Mauro Neves Muniz, Júlia Oliveira Santos, Sidnei Moura, Rafael Stieler, Cristiano Valim Bizarro, Luiz Augusto Basso, Pablo Machado

**Affiliations:** † Instituto Nacional de Ciência e Tecnologia em Tuberculose, Centro de Pesquisas em Biologia Molecular e Funcional, 28102 Pontifícia Universidade Católica do Rio Grande do Sul, 90616-900 Porto Alegre, Rio Grande do Sul, Brazil; ‡ Laboratório de Biotecnologia de Produtos Naturais e Sintéticos, 58802 Universidade de Caxias do Sul, 95070-560 Caxias do Sul, Rio Grande do Sul, Brazil; § Laboratório de Catálise Molecular, Instituto de Química, 28124 Universidade Federal do Rio Grande do Sul, 90501-970 Porto Alegre, Rio Grande do Sul, Brazil; ∥ Programa de Pós-Graduação em Biologia Celular e Molecular, Pontifícia Universidade Católica do Rio Grande do Sul, 90616-900 Porto Alegre, Rio Grande do Sul, Brazil; ⊥ Programa de Pós-Graduação em Medicina e Ciências da Saúde, Pontifícia Universidade Católica do Rio Grande do Sul, 90616-900 Porto Alegre, Rio Grande do Sul, Brazil

## Abstract

An alternative silylation–amination route for the synthesis of 3-*N*-substituted benzo­[*d*]­isothiazole 1,1-dioxides is presented. These saccharin-based scaffolds have attracted increasing interest in medicinal chemistry, particularly as potential modulators of hypoxia-inducible factor-2. The preparation of such structures is often associated with chlorination–amination approaches, which suffer from operational drawbacks, the use of chlorinating reagents, and variable efficiency. Herein, we report a one-pot silylation–amination protocol employing hexamethyldisilazane as the silylating agent. The method is operationally simple, applicable to a broad substrate scope, and affords the desired products in moderate to excellent yields (31–99%) under solvent-free conditions.

## Graphical abstract

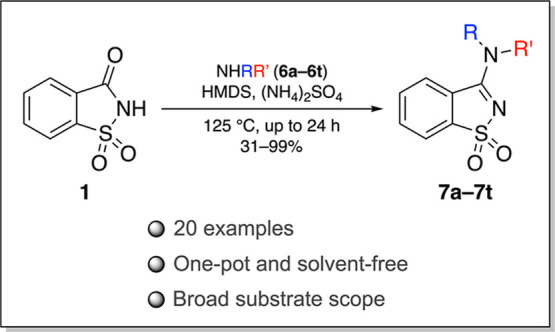

## 1 Introduction

Over the past decades, the artificial sweetener saccharin (**1**) has drawn the attention of medicinal chemists (Figure 1A). Its benzo­[*d*]­isothiazol-3­(2*H*)-one 1,1-dioxide core offers a versatile framework for both structural diversification and biological evaluation.^1,2^

**Figure 1:**
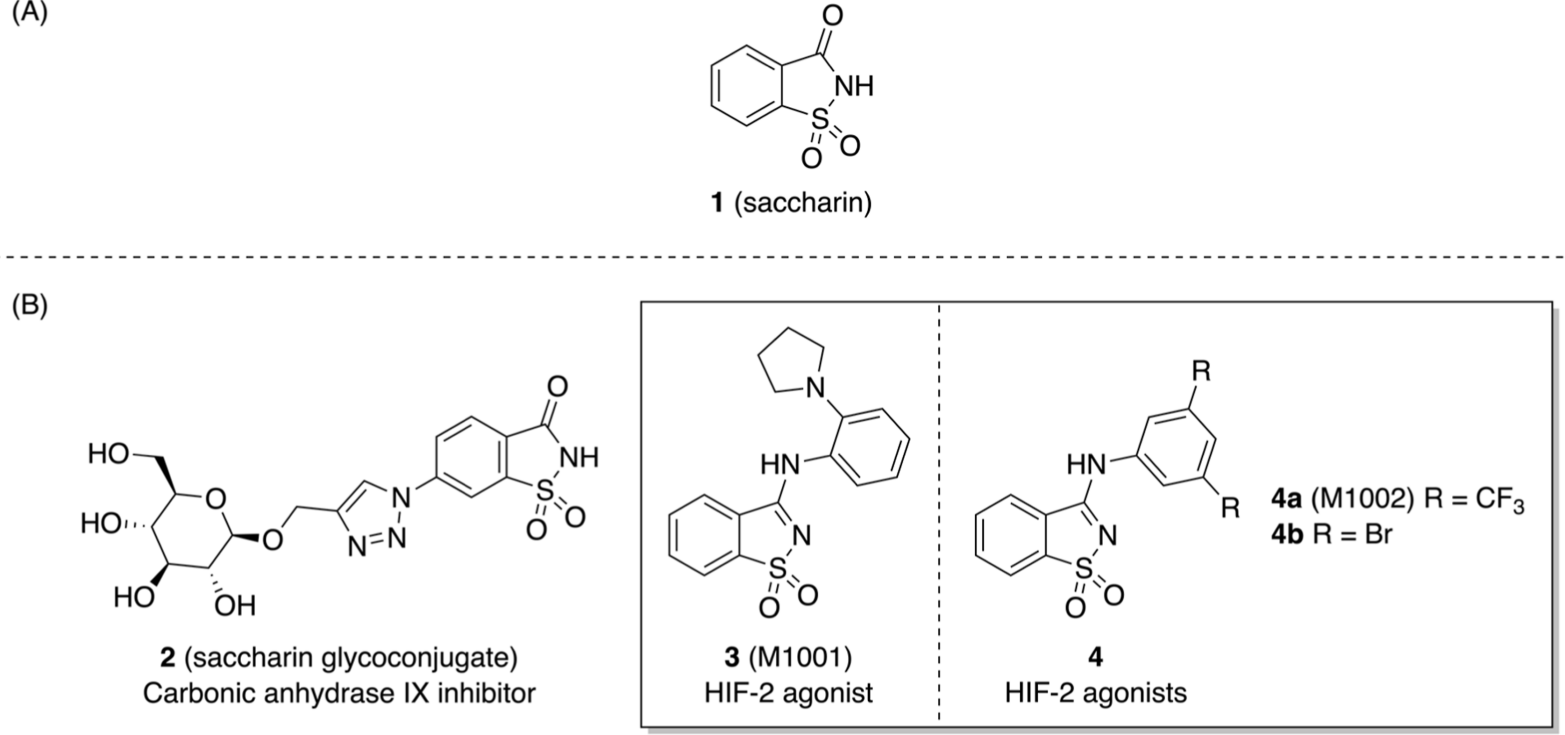
(A) Chemical structure of saccharin (**1**). (B) Saccharin derivatives (**2–4**) and their respective biological activities.

Saccharin (**1**) is a calorie-free consumable that exhibits nanomolar inhibitory activity and remarkable selectivity against the carbonic anhydrase IX (CA IX) isoform,^3^ an emerging tumor-associated target (Figure 1A).^4^ Further exploration of the benzo­[*d*]­isothiazol-3­(2*H*)-one 1,1-dioxide nucleus has led to the development of other CA IX inhibitors, such as the saccharin glycoconjugate (**2**) (Figure 1B).^5^ Beyond their activity targeting CA IX, saccharin derivatives have also been investigated as potential modulators of hypoxia-inducible factor-2 (HIF-2) in the treatment of renal anemia. An affinity selection–mass spectrometry screening assay identified M1001 (**3**) as the first-in-class HIF-2 allosteric agonist (Figure 1B).^6^ In addition, structure–activity relationship (SAR) studies based on the hit (**3**) yielded ligands M1002 (**4a**)^6^ and (**4b**) (Figure 1B),^7^ which showed improved HIF-2 agonistic effect. Aside from this activity profile, the saccharin substructure is also found in analogs that have shown fungicidal,^8^ anti-inflammatory,^9^ and antioxidant activities.^10^

Given this broad biological relevance, the development of efficient methodologies to access structurally diverse saccharin-based series in adequate yields, while minimizing environmental and operational hazards, is crucial. The amide portion of the saccharin (**1**) core is commonly subjected to structural modifications (Figure 1A). In this context, 3-*N*-substituted benzo­[*d*]­isothiazole 1,1-dioxides (e.g., **3** and **4a–4b**, Figure 1B) serve as scaffolds that occupy a chemical space frequently explored in SAR investigations.^6,7,11–14^ The standard procedure for synthesizing them involves the chlorination of the carbonyl group, followed by amination with primary or secondary amines (Scheme 1A,B).^7,11–14^ Although widely employed, the chlorination–amination approach is characterized by marked shortcomings. It requires the use of hazardous and environmentally detrimental chlorinating agents (e.g., POCl_3_, PCl_5_, and SOCl_2_).^11,12,15^ Moreover, the need for a nitrogen atmosphere during the chlorination step increases operational demands and resource consumption. Concerning the amination step, reaction yields reported by previous studies suggest that it is a process with potentially variable efficiency.^7,11–14^ Such limitations may compromise the project workflow while increasing both costs and waste generation.^16^

**Scheme 1:**
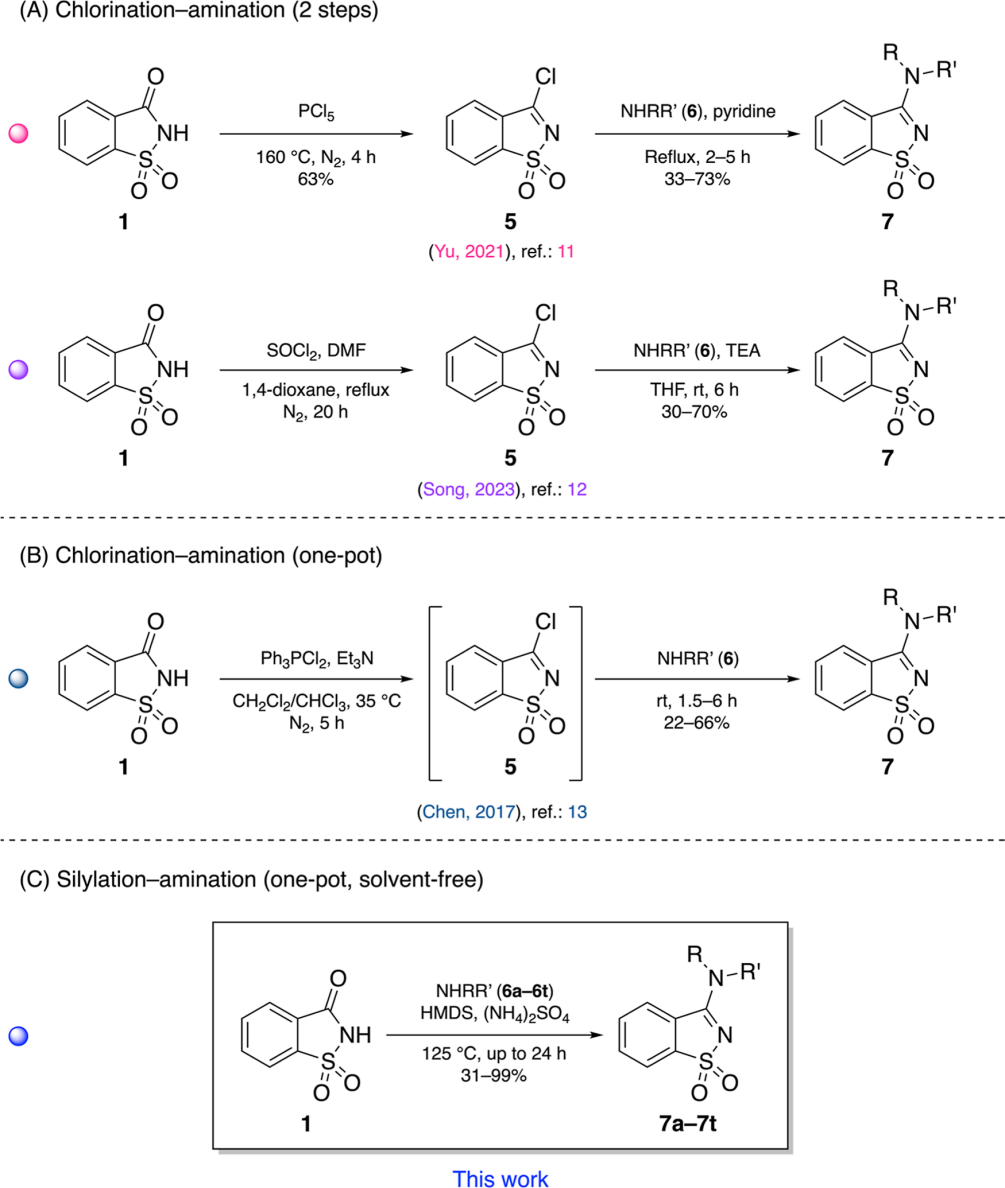
(A,B) Conventional Chlorination–Amination Routes to 3-*N*-Substituted benzo­[*d*]­isothiazole 1,1-Dioxides. (C) Silylation–Amination Strategy Proposed in This Work

In line with our research program efforts, we aim to develop alternative synthetic methodologies that deliver products in satisfactory yields while reducing environmental impact.^17,18^ The organosilicon field has been a subject of interest in our group in recent years.^18,19^ One particular procedure, known as silylation–amination, has significantly enhanced the efficiency of the synthetic routes used in our projects.^18,20^ We have developed a one-pot protocol for accessing *N*-phenethylquinazolin-4-amines under solvent-free conditions, utilizing hexamethyldisilazane (HMDS) as a silylating agent.^18^ This strategy avoided the chlorination step and afforded products with excellent yields compared to existing methodologies. Additionally, the generation of chemical waste was considerably reduced, since it did not require chlorinating agents, solvent medium (e.g., CH_2_Cl_2_ and CHCl_3_), weak bases (e.g., triethylamine and pyridine), and reaction workup.

Inspired by these results, we hypothesized that the silylation–amination method could be applied to **1** to synthesize 3-*N*-substituted benzo­[*d*]­isothiazole 1,1-dioxides (Figure 1A). Although earlier studies did not extensively explore silylation–amination on five-membered hydroxy *N*-heterocycles,^21^ literature evidence supports the feasibility of this approach for saccharin. Indeed, the benzo­[*d*]­isothiazol-3­(2*H*)-one 1,1-dioxide nucleus exhibits lactam–lactim tautomerism—a key structural feature for silylation–amination.^21^ Moreover, previous works have shown that saccharin can undergo silylation in the presence of HMDS.^22^ Such evidence reinforced the plausibility of applying the silylation–amination strategy, since the in situ formation of a silylated intermediate has been proposed to be essential for product formation.^21^ Building on this rationale, we report herein a one-pot procedure to synthesize 3-*N*-substituted benzo­[*d*]­isothiazole 1,1-dioxides from saccharin and amines mediated by HMDS (Scheme 1C).

## 2 Results and Discussion

The silylation–amination reaction was studied on the saccharin core to obtain 3-*N*-substituted benzo­[*d*]­isothiazole 1,1-dioxides. Accordingly, the synthetic strategy consisted of reacting saccharin (**1**) with primary and secondary amines (**6a–6t**) in the presence of HMDS and catalytic amounts of ammonium sulfate ((NH_4_)_2_SO_4_) to afford the desired products (**7a–7t**) (Scheme 2).

**Scheme 2:**
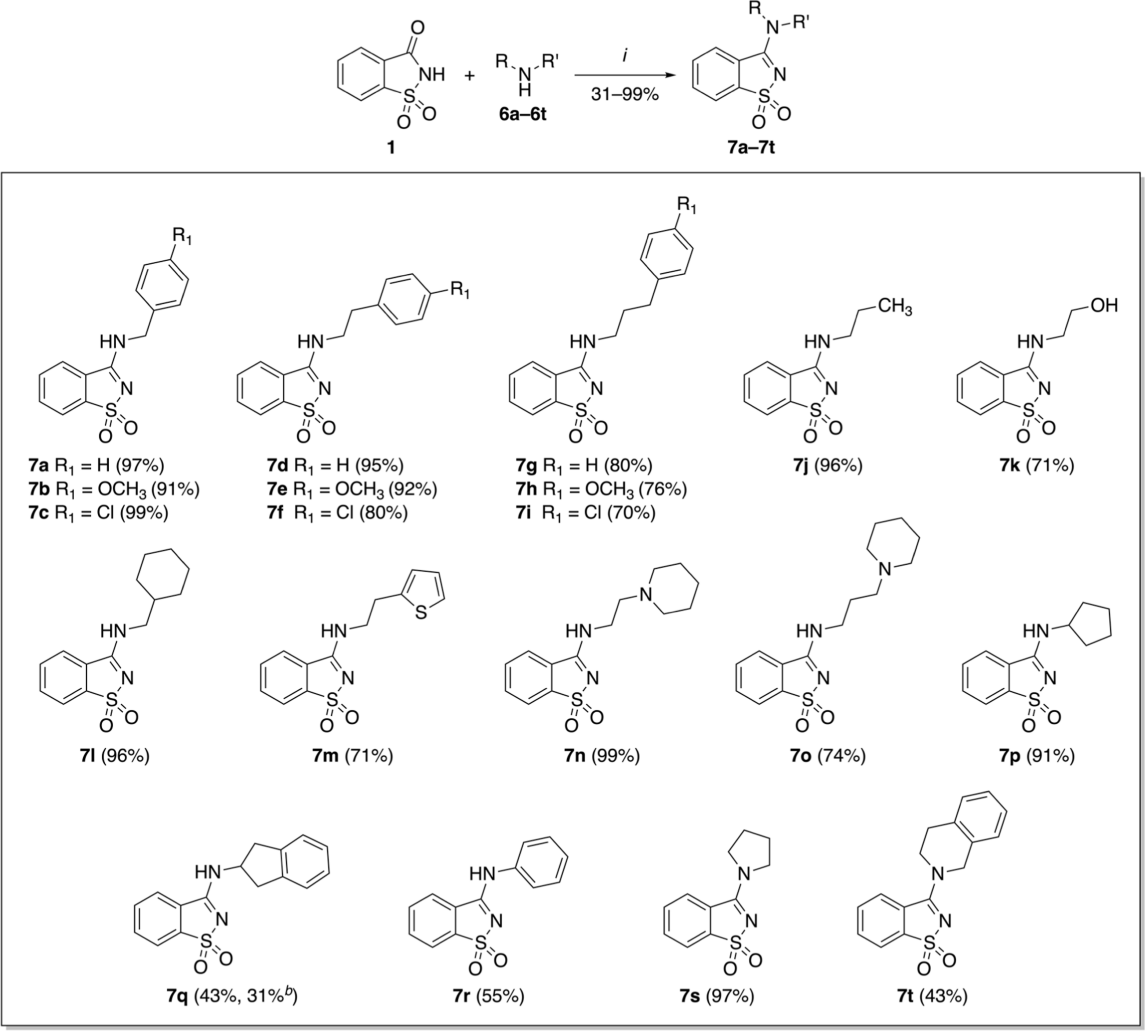
Synthesis of 3-*N*-Substituted benzo­[*d*]­isothiazole 1,1-Dioxides^a^

a Conditions and reagents are as follows: (*i*) HMDS, (NH_4_)_2_SO_4_, 125 °C, up to 24 h, 31–99%.
b Yield obtained from recrystallization.

Initial experiments used benzylamine (**6a**) as a model substrate (Scheme 2). The selection of **6a** was based on its anticipated favorable reactivity during the amination process.^21^ The latter features a readily available localized lone pair at the amine nitrogen. Furthermore, the amine moiety is sterically accessible and can therefore act as an effective nucleophile. The reaction was performed using saccharin (**1**) as the limiting reagent and 1.4 equiv of both **6a** and HMDS, corresponding to a 1:1.4:1.4 ratio (saccharin/benzylamine/HMDS). An excess of amine and HMDS was used to favor the silylation–amination process. It has been proposed that an excess of amine promotes the solubilization of the starting material, enabling proper silylation and following amination; in parallel, HMDS generates activated lipophilic silylated intermediates and, in combination with the excess amine, serves as the reaction medium.^21^ Since previous reports have shown that **1** requires a catalyst to undergo silylation with HMDS,^22^ (NH_4_)_2_SO_4_ was employed as a catalytic additive at 10 mol %. The literature identifies (NH_4_)_2_SO_4_, together with *p*-toluenesulfonic acid hydrate, trifluoromethanesulfonic acid, and perfluorobutanesulfonic acid, as among the most practical catalysts for promoting the silylation–amination process.^21^ Its usefulness was further demonstrated in an earlier report from our research group,^18^ supporting its selection as the catalyst employed in the present work. The reactants were heated at 125 °C under solvent-free conditions, corresponding to the boiling point of HMDS and commonly employed to promote efficient silylation.^21,23,24^ It should be noted that the conversion was achieved without a nitrogen or other inert gas atmosphere, indicating that such conditions are not required. Afterward, the crude reaction material was purified by column chromatography and provided the expected product 3-(benzylamino)­benzo­[*d*]­isothiazole 1,1-dioxide (**7a**) in 97% yield. The proposed structure of **7a** was confirmed by spectroscopic and spectrometric data (Supporting Information) and corroborated by earlier studies.^11,13,25^ These conditions were subsequently applied to evaluate the substrate scope.

After the encouraging results achieved with **6a**, we investigated the applicability of the developed protocol using primary (**6b–6r**) and secondary amines (**6s–6t**) (see the Supporting Information for the list of amines) (Scheme 2). A total of 20 3-*N*-substituted benzo­[*d*]­isothiazole 1,1-dioxide examples (**7a–7t**) were synthesized, with yields ranging from 31–99%. As starting material **1** was fully consumed in all cases, variations in the isolated yields may be associated with purification efficiency rather than reaction conversion. In addition, the low solubility observed for some products may have affected their isolation, and these aspects are addressed where appropriate. The structures of the synthesized compounds **7a–7t** were confirmed by spectroscopic and spectrometric data (Supporting Information).

Similar results were obtained for other benzylamine derivatives bearing electron-donating (**7b**, R_1_ = OCH_3_, 91%) and electron-withdrawing (**7c**, R_1_ = Cl, 99%) substituents at the *para* position (Scheme 2). The phenethylamine products **7d** (R_1_ = H, 95%) and **7e** (R_1_ = OCH_3_, 92%) displayed comparable yields to their corresponding benzylamine analogues, whereas compound **7f** (R_1_ = Cl) exhibited a slight reduction (80%). The phenylpropylamine-based derivatives **7g** (R_1_ = H, 80%), **7h** (R_1_ = OCH_3_, 76%), and **7i** (R_1_ = Cl, 70%) afforded lower yields compared to shorter-chain homologues. Regarding products **7h** and **7i**, it is noteworthy that the protocol was adapted due to the salt form of the amines employed (**6h** and **6i**) in their respective reactions. Preliminary experiments with **6h** were unsuccessful due to the absence of an effective reaction medium, leading to a completely solid reaction mixture. We progressively increased the amount of HMDS and reached a proportion of 4.0 equiv to ensure complete solubilization of the reactants. Overall, the reaction times for products **7a–7i** were up to 4 h; notably, compound **7f** was obtained within 5 min.

The proposed protocol also went smoothly with propylamine (**6j**), providing the product **7j** after 2 h in 96% yield (Scheme 2). In contrast, silylation–amination with cyclohexanemethylamine (**6l**) was completed within 20 min, yielding **7l** in 96%. Concerning the aminoalcohol-based derivative **7k**, additional steps were applied. The limiting reagent was consumed after 25 min, although the reaction did not proceed directly to the product **7k**. This outcome was anticipated due to the hydroxyl group of ethanolamine (**6k**), which is prone to silylation upon treatment with HMDS.^21^ Prior investigations involving purines and nucleosides have proposed that additional hydroxyl or amino functionalities can undergo silylation under comparable conditions, and the corresponding trimethylsilyl group can be removed.^26,27^ Thus, treatment of the putative silylated analogue of **7k** with a methanol–water mixture provided the desired derivative **7k** in 71% yield after purification.

The heterocycle-containing derivatives **7m–7o**, obtained from amines **6m–6o**, were synthesized in good to excellent yields (71–99%) (Scheme 2). Compared to the phenethylamine analogue **7d** (R_1_ = H, 2 h, 95%), the thiopheneethylamine derivative compound **7m** was obtained in a shorter reaction time (10 min) and isolated in a lower, although acceptable, yield of 71%. Rapid silylation–amination was also observed with 2-(piperidin-1-yl)­ethan-1-amine (**6n**), affording the piperidine-based product **7n** within 15 min in 99% yield. However, a lower yield (74%) was obtained for the longer-chain homologue **7o**, a trend also observed for derivatives **7d**–**7i**. Although this observation may suggest a relationship between the number of methylene units in the alkyl chain and the isolated yields, this interpretation should be made with caution, given the limited data set and the possible contribution of other factors (e.g., differences in product solubility and purification method efficiency).

Benzoisothiazole 1,1-dioxides **7p** and **7q,** bearing cyclopentylamine and aminoindane functionalities, respectively, provided contrasting results (Scheme 2). Silylation–amination with cyclopentylamine (**6p**) was achieved within 35 min and gave product **7p** in 91% yield. Conversely, when reacted with 2,3-dihydro-1*H*-inden-2-amine (**6q**), the starting material **1** required 4 h for its complete consumption. Since amine **6q** was used in its salt form, the protocol was adapted using the same conditions described for **6h** and **6i**. The crude reaction mixture was partially soluble in most solvents; hence, we increased the polarity of the mobile phase (hexane–ethyl acetate, 2:8) to promote solubilization and ensure proper purification via column chromatography. Despite these adjustments, **7q** was isolated in moderate yield (43%) with impurities, and subsequently purified by recrystallization from acetone (31%). The poor solubility of **7q** could explain the reduced efficiency of purification. Nevertheless, the latter added structural diversity to the series, as it can be regarded as the phenylogous analogue of **7p** and a conformationally restricted homologue of phenethylamine-based **7d**.

To further assess the scope of the protocol, aniline (**6r**) was evaluated as a less nucleophilic primary amine (Scheme 2). Complete consumption of the starting material **1** required extended time (24 h), providing compound **7r** in 55% yield. The longer reaction time may be associated with delocalization of the nitrogen lone pair of **6r** into the aromatic π-system, thereby compromising amination effectiveness. Similar to **7q**, the moderate yield could be attributed to purification efficiency, as both the crude reaction mixture and the isolated product **7r** were poorly soluble in various solvents.

Secondary amines, such as pyrrolidine (**6s**) and 1,2,3,4-tetrahydroisoquinoline (**6t**), were included to explore the generality of the silylation–amination protocol (Scheme 2). Reaction with **6s** gave compound **7s** within 30 min in excellent yield (97%). By contrast, we found that the bulkier **6t** reached completion in 4 h to afford product **7t**. The crude reaction mixture was insoluble in most solvents, preventing purification by column chromatography. As a result, compound **7t** was isolated in 43% yield after multiple washings with an acetonitrile–methanol mixture. Likewise, derivative **7t** may be viewed as a conformationally restricted analogue of both benzylamine-derived **7a** and phenethylamine-based **7d**.

Furthermore, to demonstrate the performance of the developed protocol, representative literature examples of 3-*N*-substituted benzo­[*d*]­isothiazole 1,1-dioxides were compared with the present methodology (Table 1). Most of the products synthesized from the silylation–amination approach exhibited higher isolated yields than those obtained using the chlorination–amination method. The present methodology afforded a higher average yield (84.9%, *n* = 7) than the mean literature value (51.4%, *n* = 11), representing an approximate 65% relative improvement. As discussed previously, the moderate yield observed for compound **7r** (55%) may be attributed to its low solubility, potentially impacting the efficiency of the purification process. In general, reported results indicate that the chlorination–amination strategy can lead to variable outcomes, as exemplified by compounds **7a** (50–85%)^11,13,25^ and **7r** (22–82%).^7,12,13^ These earlier strategies rely on preactivation steps, chlorinating agents, and solvent-based conditions. By comparison, the present protocol provided products with satisfactory yields in a one-pot procedure under solvent-free conditions.

**Table 1:**
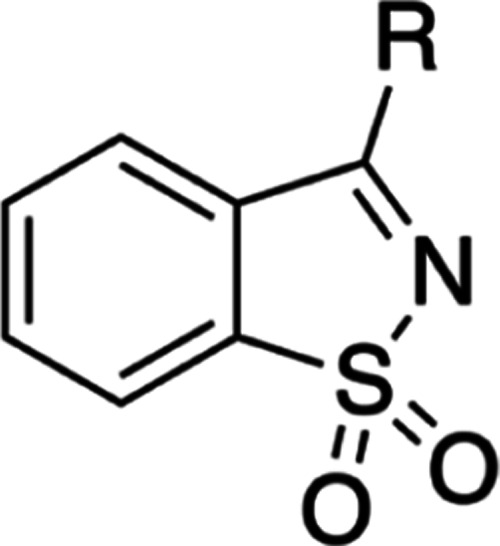

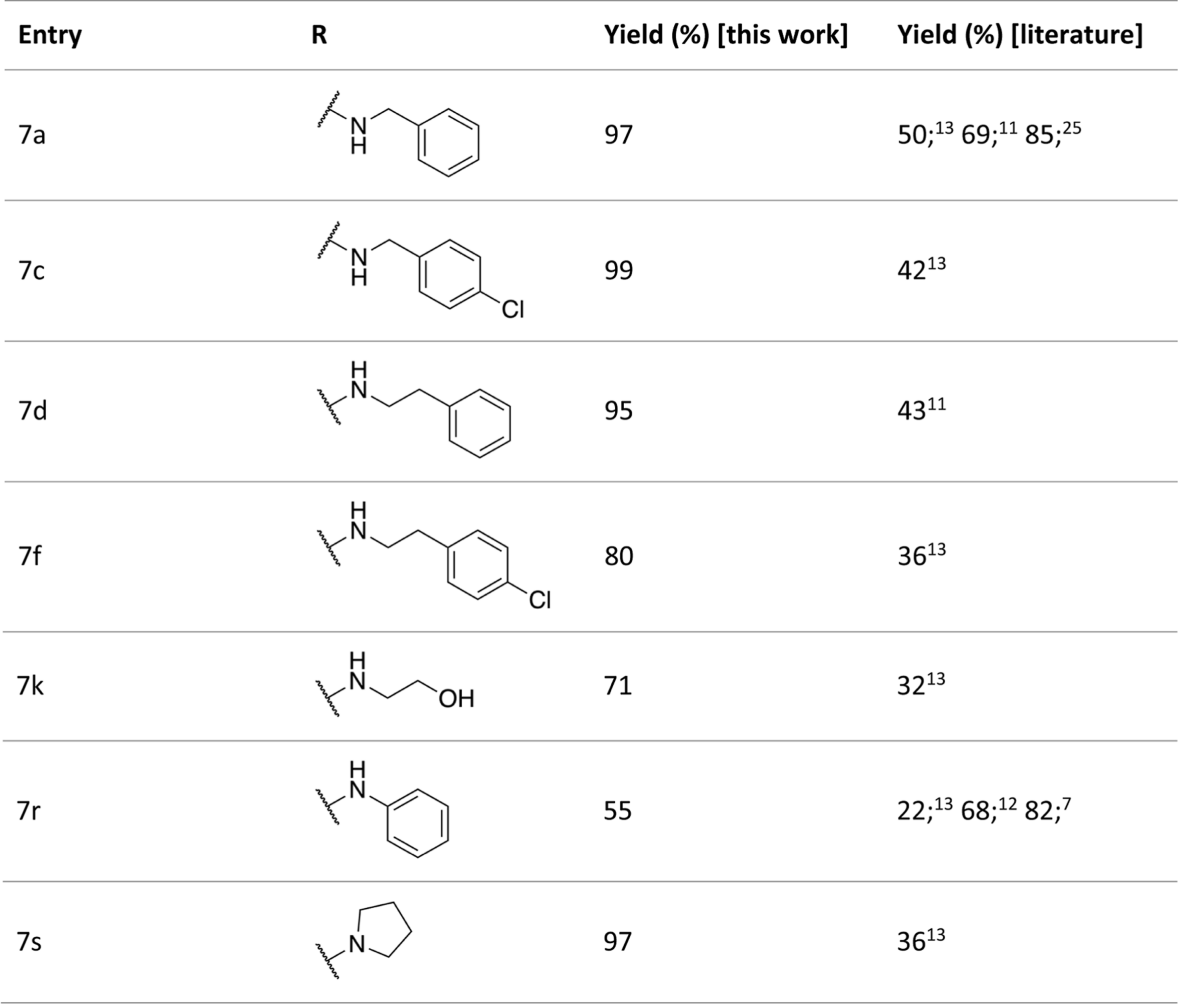
Comparison of Isolated Yields for Selected 3-*N*-substituted benzo­[*d*]­isothiazole 1,1-Dioxides Obtained by Silylation–Amination (This Work) and Chlorination–Amination Methods Reported in the Literature

To assess the scalability of the protocol, the synthesis of 3-(phenethylamino)­benzo­[*d*]­isothiazole 1,1-dioxide (**7d**) (Scheme 2) was carried out on a 10-fold scale (from 0.5 to 5.0 mmol of **1**) while maintaining identical molar ratios of all reagents. Complete consumption of **1** was achieved within 2 h, in agreement with the previously determined reaction time. After reaction workup, the crude product was purified by recrystallization from acetonitrile, affording **7d** in 60% yield without the need for chromatographic purification.

Single-crystal X-ray diffraction analysis (SC-XRD) was performed to unequivocally confirm the molecular structure of the synthesized 3-*N*-substituted benzo­[*d*]­isothiazole 1,1-dioxide. The derivative 3-(propylamino)­benzo­[*d*]­isothiazole 1,1-dioxide (**7j**) (Scheme 2) crystallized as colorless single plate-shaped crystals suitable for SC-XRD data collection. The molecular structure of **7j** (C_10_H_12_N_2_O_2_S), which crystallizes in the triclinic P1̅ space group, is shown in Figure 2. Briefly, the S1–O1 (1.432 Å) and S1–O2 (1.443 Å) bond lengths are very similar, with O1–S1–O2 bond angle of 114.86°. The S1–N2 (1.615 Å) and S1–C1 (1.762 Å) are the longest bonds distances in the molecule and are associated with the S1 atom in the saccharyl core. On the other hand, it is noteworthy that N2–C7 (1.329 Å) and N1–C7 (1.314 Å) are shorter than N1–C8 (1.462 Å) and C6–C7 (1.485 Å), which is consistent with partial double-bond character in the N1–C7–N2 core. These observations are comparable with those reported for the ethylamine homologue 3-(ethylamino)­benzo­[*d*]­isothiazole 1,1-dioxide.^28^ The detailed crystallographic data are available in the Supporting Information.

**Figure 2:**
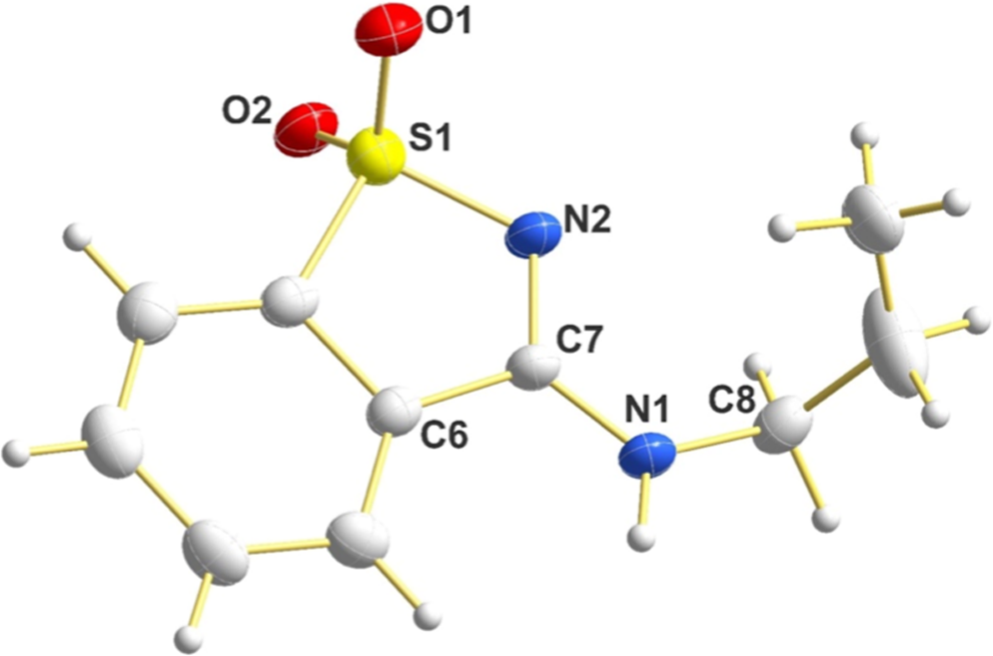
Molecular structure of 3-(propylamino)­benzo­[*d*]­isothiazole 1,1-dioxide (**7j**), with the main atoms labeled. Thermal ellipsoids are drawn at the 50% probability level. The terminal methyl group is disordered over two positions; for clarity, only one of them is shown.

Finally, a plausible silylation–amination reaction pathway to 3-*N*-substituted benzo­[*d*]­isothiazole 1,1-dioxides is proposed (Scheme 3). Ammonium sulfate ((NH_4_)_2_SO_4_) may assist HMDS activation via proton transfer to form bis­(trimethylsilyl)­ammonium, with ammonia as side product. Subsequently, bis­(trimethylsilyl)­ammonium may exhibit a polarized N–Si bond due to protonation, thereby promoting trimethylsilylation of the lactim tautomer of saccharin (**1**). Silylation of **1** generates trimethylsilanamine, which deprotonates the positively charged species to give the silylated intermediate (**1-OTMS**), with (trimethylsilyl)-λ^4^-azane as a side product. Next, the amination step occurs via nucleophilic attack of amine **6** at the electron-deficient carbon of **1-OTMS**, leading to the formation of a tetrahedral intermediate (**1-TI**). This proposal is supported by the putative tetrahedral adduct isolated and characterized (Supporting Information). The addition affording **1-TI** is suggested to be stabilized by resonance and inductive effects exerted by the sulfonyl moiety. Afterward, elimination of the trimethylsilyloxy group occurs, resulting in the negatively charged trimethylsilanolate side product. Ultimately, trimethylsilanolate neutralizes the aminated species, yielding 3-*N*-substituted benzo­[*d*]­isothiazole 1,1-dioxide (**7**), with trimethylsilanol as a side product. In parallel, (trimethylsilyl)-λ^4^-azane serves as a source for an additional trimethylsilylation reaction. The silylated species is deprotonated by the ammonia formed as a side product, resulting in **1-OTMS** and promoting the regeneration of ((NH_4_)_2_SO_4_).

**Scheme 3:**
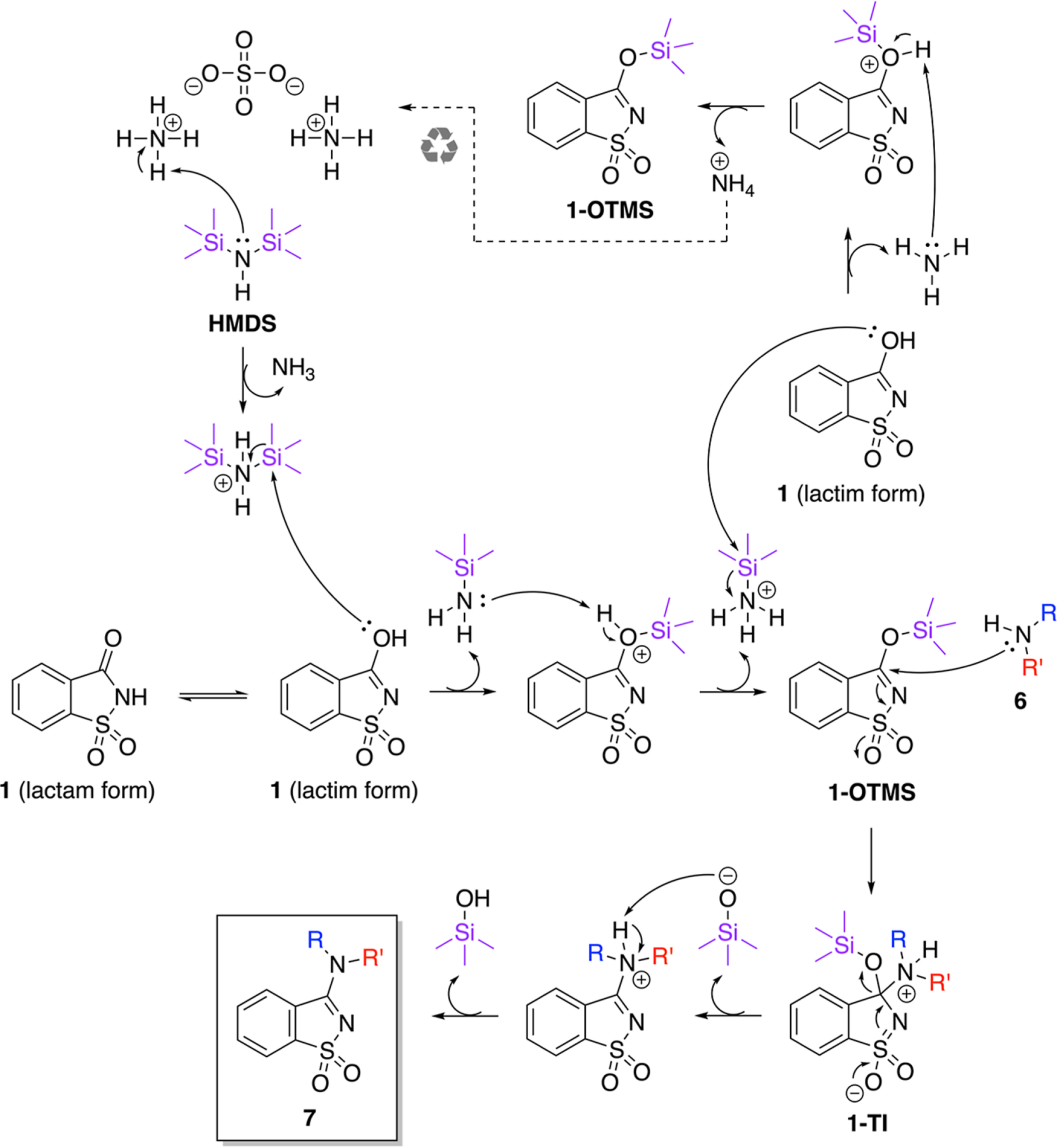
Plausible Silylation–Amination Reaction Pathway to 3-*N*-Substituted benzo­[*d*]­isothiazole 1,1-Dioxides

## 3 Conclusions

Overall, we developed an alternative synthetic route to access 3-*N*-substituted benzo­[*d*]­isothiazole 1,1-dioxides based on the silylation–amination strategy. Earlier studies employed chlorination–amination methodologies, which are associated with both operational and environmental issues, additional steps, and variable yields. These limitations motivated the investigation of more efficient approaches, directing our efforts toward organosilicon chemistry. Notably, the hypothesis that the silylation–amination mediated by HMDS could be applied to saccharin proved fruitful. Twenty 3-*N*-substituted benzo­[*d*]­isothiazole 1,1-dioxides were synthesized with moderate to excellent yields (31–99%) in a one-pot procedure. The protocol was consistent across a range of substrates, avoided the chlorination step, and enabled product formation under solvent-free conditions. Finally, given the growing interest in saccharin and its derivatives in medicinal chemistry, this methodology may facilitate a more efficient exploration of both known and novel chemical spaces.

## 4 Experimental Section

### 4.1 General Methods

All solvents and reagents were obtained from commercial sources and used without further purification. When relevant, the relative purity of some reagents was determined by high-performance liquid chromatography (HPLC). The progress of the reaction was monitored using thin-layer chromatography (TLC) with Merck TLC Silica gel 60 F254. The products were purified by column chromatography on silica gel 60 Å (70–230 mesh, 0.063–0.200 mm). The mobile phases were previously determined based on TLC analysis (*R*_f_ ≈ 0.5). The mobile phases used for each synthesized compound are described in Supporting Information. Melting points (m.p.) were determined using a Microquímica MQAPF-302 apparatus. Fourier transform infrared (FT-IR) spectra were recorded on PerkinElmer Spectrum 100 FT-IR spectrometer with a Universal Attenuated Total Reflectance (UATR) sampling accessory. Spectra were collected over the 4000–650 cm^–1^ range with 4 scans. Stretching and bending vibrations were expressed in cm^–1^. Data acquisition and processing were performed using the Spectrum software (PerkinElmer). ^1^H and ^13^C nuclear magnetic resonance (NMR) spectra were acquired on an Avance III HD Bruker spectrometer (Bruker Corporation, Fällanden, Switzerland) with standard pulse sequences operating at 400 MHz for ^1^H nuclei and 100 MHz for ^13^C nuclei. Chemical shifts (δ) were expressed in parts per million (ppm) relative to DMSO-*d*_6_, which were used as the solvent, and to trimethylsilane, as an internal standard. The data were acquired according to the instrument parameters and processed using MestReNova 14.2.3 software (Mestrelab Research). Compound purity was measured using a Dionex UltiMate 3000 HPLC system (Thermo Fisher Scientific Inc., Waltham, MA, USA) equipped with a dual pump, automatic injector, and UV detector. Stock solutions (1.0 mg/mL) of each product were prepared in acetonitrile–methanol (1:1, v/v) and diluted to 0.5 mg/mL for analysis. For data acquisition and processing, calculations were performed using the Chromeleon 6.80 SR11 software Build 3160 (183,147). The HPLC conditions: reversed-phase column, 5 μm Nucleodur C-18 (250 × 4.6 mm); flow rate, 1.5 mL/min; UV detection at 254 nm; 100% water (1% acetic acid) was maintained from 0 to 7 min, followed by a linear gradient from 100% water (1% acetic acid) to 90% acetonitrile–methanol (1:1, v/v) from 7 to 15 min and subsequently returned to 100% water (1% acetic acid) in 5 min and maintained for more 10 min. All the synthesized compounds were ≥95% pure. High-resolution mass spectrometry (HRMS) analyses were performed on Bruker MicroTOF-QII using electrospray ionization (ESI) (Universidade de Caxias do Sul, Brazil). Sample solutions in methanol were individually infused into the ESI source using a syringe pump (Harvard Apparatus, Hamilton, Reno, USA) at a flow rate of 150 μL min^–1^. The ESI­(+)-MS and tandem ESI­(+)-MS/MS profiles were obtained under the following conditions: capillary voltage of +3500 V, cone voltage of +40 V, and desolvation temperature of 100 °C. For ESI­(+)-MS/MS, the collision energy for collision-induced dissociation was optimized for each component. Data were acquired over the *m*/*z* range 100–2000 at a scan rate of two scans s^–1^, providing a resolution of 18.000 (fwhm) at *m*/*z* 200. Data acquisition and processing were performed using the DataAnalysis software (Bruker Scientific). Single-crystal X-ray diffraction (SC-XRD) data were collected on a Bruker D8 QUEST Fixed Chi Diffractometer (Analytical Central Service, Institute of Chemistry, Universidade Federal do Rio Grande do Sul, Brazil) equipped with a PHOTON IV CPAD detector and an Oxford Cryostream 1000 low-temperature device using Mo Kα radiation (λ = 0.71073 Å). The crystallographic data for the structure reported in this paper have been deposited in the Cambridge Crystallographic Data Centre with CCDC number 2535307. These data can be obtained free of charge from The Cambridge Crystallographic Data Centre via www.ccdc.cam.ac.uk/structures.

### 4.2 General Procedure for the Synthesis of 3-*N*-substituted benzo­[*d*]­isothiazole 1,1-dioxides (**7a–7t**)

In a sealed 10 mL Schlenk tube, saccharin (**1**) (0.5 mmol, 1.0 equiv), amine (**6a–6t**) (0.7 mmol, 1.4 equiv), hexamethyldisilazane (HMDS) (0.7 mmol, 1.4 equiv), and ammonium sulfate ((NH_4_)_2_SO_4_) (0.05 mmol, 0.1 equiv) were heated at 125 °C (Scheme 2). Four equivalents (4.0 equiv) of HMDS were employed when the amine was used in its salt form. The reaction progress was monitored by TLC using mixtures of hexane–ethyl acetate, typically starting from a 1:1 ratio and adjusting the polarity as needed. Following reaction completion, the material was solubilized, transferred to a 100 mL round-bottom flask, and concentrated using a rotary evaporator. The crude solid was purified by column chromatography using a proper solvent mixture under isocratic conditions. The mobile phases used for purification and the reaction times are specified in the product descriptions (Supporting Information).

## Supplementary Material



## Notes

Experimental section, synthetic procedures, analytical, spectroscopy, and spectrometry data. ^1^H and ^13^C NMR spectra for all synthesized compounds, single-crystal X-ray diffraction analysis, and additional experiments are also provided (PDF)

## Abbreviations

CA IX: carbonic anhydrase IX
HIF-2: hypoxia-inducible factor-2
SAR: structure–activity relationship
HMDS: hexamethyldisilazane
HPLC: high-performance liquid chromatography
TLC: thin-layer chromatography
m.p.: melting point
FT-IR: Fourier transform infrared
UATR: universal attenuated total reflectance
NMR: nuclear magnetic resonance
ppm: parts per million
HRMS: high-resolution mass spectrometry
ESI: electrospray ionization
SC-XRD: single-crystal X-ray diffraction
equiv.: equivalent.
