# Supplementary material for: One-Pot Silylation–Amination Synthesis of 3‑N‑Substituted Benzo[d]isothiazole 1,1-Dioxides
Source: ACS Omega. 2026 Jul 6;11(28):42537–45. doi: 10.1021/acsomega.6c03641 (PMC13393046; doi:10.1021/acsomega.6c03641)
Supplement: Supplementary file 1 [file ao6c03641_si_001.pdf]

Supporting Information

One-Pot Silylation–Amination Synthesis of 3-*N*-Substituted Benzo[*d*]isothiazole 1,1-Dioxides

Guilherme Arraché Gonçalves[1], Lídia Klatt Oliveira[1], Mauro Neves Muniz[1], Júlia Oliveira Santos[1], Sidnei Moura[2], Rafael Stieler[3], Cristiano Valim Bizarro[1,4], Luiz Augusto Basso[1,4,5], Pablo Machado*[1,4,5]

[1] Instituto Nacional de Ciência e Tecnologia em Tuberculose, Centro de Pesquisas em Biologia Molecular e Funcional, Pontifícia Universidade Católica do Rio Grande do Sul, 90616-900 Porto Alegre, Rio Grande do Sul, Brazil

[2] Laboratório de Biotecnologia de Produtos Naturais e Sintéticos, Universidade de Caxias do Sul, 95070-560 Caxias do Sul, Rio Grande do Sul, Brazil

[3] Laboratório de Catálise Molecular, Instituto de Química, Universidade Federal do Rio Grande do Sul, 90501-970 Porto Alegre, Rio Grande do Sul, Brazil

[4] Programa de Pós-Graduação em Biologia Celular e Molecular, Pontifícia Universidade Católica do Rio Grande do Sul, 90616-900 Porto Alegre, Rio Grande do Sul, Brazil

[5] Programa de Pós-Graduação em Medicina e Ciências da Saúde, Pontifícia Universidade Católica do Rio Grande do Sul, 90616-900 Porto Alegre, Rio Grande do Sul, Brazil

*Corresponding author: Pablo Machado (pablo.machado@pucrs.br; Tel.: +55-51-3320-3629)

Supporting Information

TABLE OF CONTENTS

1. MATERIALS AND METHODS 3

1.1. General methods 3

2. EXPERIMENTAL SECTION 5

2.1. General procedure for the synthesis of 3-*N*-substituted benzo[*d*]isothiazole 1,1-dioxides (**7a**–**7t**) 5

2.2. Isolation and characterization of a putative tetrahedral adduct (**7c**-adduct) from the reaction leading to **7c** 19

2.3. Attempted silylation–amination of benzo[*d*]isothiazol-3(2*H*)-one (**8**) using the developed protocol 20

2.4. Scale-Up of 3-(phenethylamino)benzo[*d*]isothiazole 1,1-dioxide (**7d**) 21

2.5. Experimental notes 22

3. ANALYTICAL DATA 24

3.1. ^{1}H and ^{13}C NMR spectra of the synthesized compounds 24

3.2. Spectroscopic and mass spectrometric data of the putative tetrahedral adduct (**7c**-adduct) 44

3.3. Single-crystal X-ray diffraction analysis of 3-(propylamino)benzo[*d*]isothiazole 1,1-dioxide (**7j**) 46

4. REFERENCES 55

Supporting Information

1. MATERIALS AND METHODS

1.1. General methods

All solvents and reagents were obtained from commercial sources and used without further purification. When relevant, the relative purity of some reagents was determined by high-performance liquid chromatography (HPLC). The progress of the reaction was monitored using thin-layer chromatography (TLC) with Merck TLC Silica gel 60 F254. The products were purified by column chromatography on silica gel 60 Å (70–230 mesh, 0.063–0.200 mm). The mobile phases were previously determined based on TLC analysis ($R_f \approx 0.5$). The mobile phases used for each synthesized compound are described in Section 2.1. Melting points (m.p.) were determined using a Microquímica MQAPF-302 apparatus and were uncorrected. Fourier transform infrared (FT-IR) spectra were recorded on Perkin-Elmer Spectrum 100 FT-IR spectrometer with a Universal Attenuated Total Reflectance (UATR) sampling accessory. Spectra were collected over the 4000–650 cm^{-1} range with 4 scans. Stretching and bending vibrations were expressed in cm^{-1}. Data acquisition and processing were performed using the Spectrum software (PerkinElmer). ^{1}H and ^{13}C nuclear magnetic resonance (NMR) spectra were acquired on an Avance III HD Bruker spectrometer (Bruker Corporation, Fällanden, Switzerland) with standard pulse sequences operating at 400 MHz for ^{1}H nuclei and 100 MHz for ^{13}C nuclei. Chemical shifts (δ) were expressed in parts per million (ppm) relative to DMSO-d_6, which were used as the solvent, and to trimethylsilane, as an internal standard. The data were acquired according to the instrument parameters and processed using MestReNova 14.2.3 software (Mestrelab Research). Compound purity was measured using a Dionex UltiMate 3000 HPLC system (Thermo Fisher Scientific Inc., Waltham, MA, USA) equipped with a dual pump, automatic injector, and UV detector. Stock solutions (1.0 mg/mL) of each product were prepared in acetonitrile–methanol (1:1, v/v) and diluted to 0.5 mg/mL for analysis. For data acquisition and processing, calculations were performed using the Chromeleon 6.80 SR11 software Build 3160 (183147). The HPLC conditions: reversed-phase column, 5 µm Nucleodur C-18 (250 × 4.6 mm); flow rate, 1.5 mL/min; UV detection at 254 nm; 100% water (1% acetic acid) was maintained from 0 to 7 min, followed by a linear gradient from 100% water (1% acetic acid) to 90% acetonitrile–methanol (1:1, v/v) from 7 to 15 min and subsequently returned to 100% water (1% acetic acid) in 5 min and maintained for more 10 min. All the synthesized compounds were ≥ 95% pure. High-resolution mass spectrometry (HRMS) analyses were performed on Bruker MicroTOF-QII using electrospray ionization (ESI) (Universidade de Caxias do Sul, Brazil). Sample solutions in methanol

were individually infused into the ESI source using a syringe pump (Harvard Apparatus, Hamilton, Reno, USA) at a flow rate of 150 µL min^{-1}. The ESI(+)-MS and tandem ESI(+)-MS/MS profiles were obtained under the following conditions: capillary voltage of +3500 V, cone voltage of +40 V, and desolvation temperature of 100 °C. For ESI(+)-MS/MS, the collision energy for collision-induced dissociation was optimized for each component. Data were acquired over the *m/z* range 100–2000 at a scan rate of two scans s^{-1}, providing a resolution of 18.000 (FWHM) at *m/z* 200. Data acquisition and processing were performed using the DataAnalysis software (Bruker Scientific). Single-crystal X-ray diffraction (SC-XRD) data were collected on a Bruker D8 QUEST Fixed Chi Diffractometer (Analytical Central Service, Institute of Chemistry, Universidade Federal do Rio Grande do Sul, Brazil) equipped with a PHOTON IV CPAD detector and an Oxford Cryostream 1000 low-temperature device using Mo Kα radiation (λ = 0.71073 Å). The crystallographic data for the structure reported in this paper have been deposited in the Cambridge Crystallographic Data Centre with CCDC number 2535307. These data can be obtained free of charge from The Cambridge Crystallographic Data Centre via www.ccdc.cam.ac.uk/structures.

2. EXPERIMENTAL SECTION

2.1. General procedure for the synthesis of 3-*N*-substituted benzo[*d*]isothiazole 1,1-dioxides (7a–7t)

In a sealed 10 mL Schlenk tube, saccharin (**1**) (0.5 mmol, 1.0 equiv.), amine (**6a–6t**) (0.7 mmol, 1.4 equiv.), hexamethyldisilazane (HMDS) (0.7 mmol, 1.4 equiv.), and ammonium sulfate ($(NH_4)_2SO_4$) (0.05 mmol, 0.1 equiv.) were heated at 125 °C (**Scheme S1**). Four equivalents (4.0 equiv.) of HMDS were employed when the amine was used in its salt form. The reaction progress was monitored by TLC using mixtures of hexane–ethyl acetate, typically starting from a 1:1 ratio and adjusting the polarity as needed. Following reaction completion, the material was solubilized, transferred to a 100 mL round-bottom flask, and concentrated using a rotary evaporator. The crude solid was purified by column chromatography using a proper solvent mixture under isocratic conditions. The mobile phases used for purification and the reaction times are specified in the product descriptions.

Scheme S1. Synthesis of 3-*N*-substituted benzo[*d*]isothiazole 1,1-dioxides.[a]

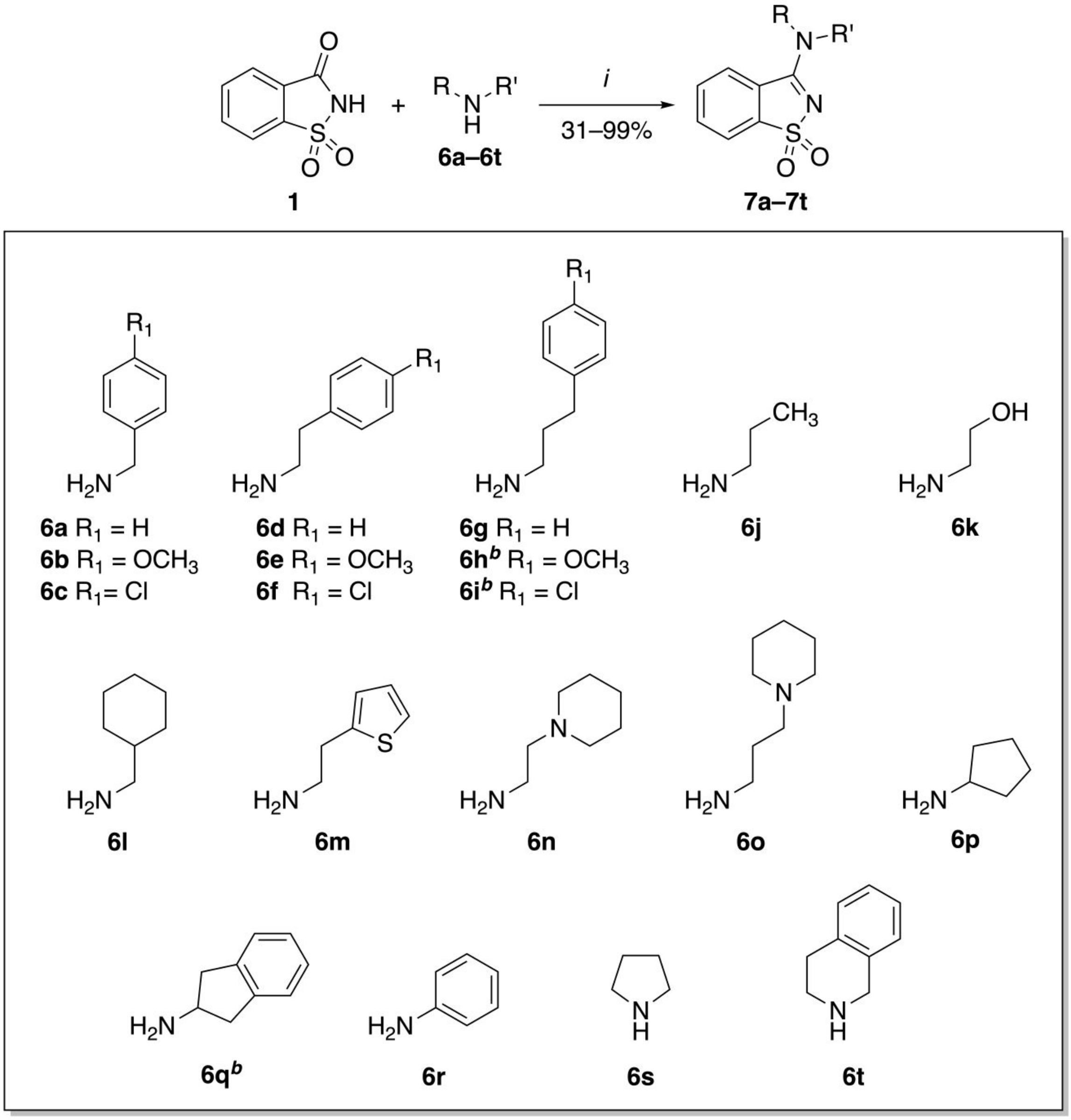

[a]Conditions and reagents are as follows: *i*) HMDS, $(NH_4)_2SO_4$, 125 °C, up to 24 h, 31–99%.

[b]Amines used as their hydrochloride salts (.HCl).

3-(benzylamino)benzo[*d*]isothiazole 1,1-dioxide (7a). Product obtained according to the general procedure described in Section 2.1, using amine (**6a**). Reaction time: 120 min (2 h). Column chromatography on silica gel (hexane–ethyl acetate, 4:6). Appearance: yellowish white solid. Yield: 97%, 132 mg. m.p. 209 °C. HPLC: 99.7% at 254 nm (t_R = 15.7 min). FT-IR (ATR cm^{-1}): 1148 (S=O), 1279 (S=O), 1348 (C-N), 1591 (C=C), 1621 (C=N), 2853 (-CH_2-), 2922 (-CH_2-), 3053 (C-H_{arom}), 3112 (C-H_{arom}), 3309 (N-H). ^1H NMR (400 MHz, DMSO-d_6) δ ppm: 4.71 (s, 2H, CH_2), 7.26 – 7.35 (m, 1H, CH_{arom}), 7.34 – 7.44 (m, 4H, CH_{arom}), 7.79 – 7.87 (m, 2H, CH_{arom}), 8.00 (dd, *J* = 2.8, 5.9 Hz, 1H, CH_{arom}), 8.23 (dd, *J* = 3.0, 5.7 Hz, 1H, CH_{arom}), 9.94 (s, 1H, NH). ^{13}C (100 MHz, DMSO-d_6) δ ppm: 45.98, 121.23, 123.02, 127.49, 127.61, 127.72, 128.54, 133.13, 133.53, 137.11, 142.25, 159.48. HRMS (ESI): *m/z* calcd for $C_{14}H_{13}N_2O_2S$ $[M + H]^+$: 273.0692; found, 273.0686.

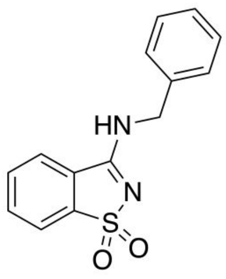

7a

3-((4-methoxybenzyl)amino)benzo[*d*]isothiazole 1,1-dioxide (7b). Product obtained according to the general procedure described in Section 2.1 using amine (**6b**). Reaction time: 60 min (1 h). Column chromatography on silica gel (hexane–ethyl acetate, 4:6). Appearance: white solid. Yield: 91%, 137 mg. m.p. 187–188 °C. HPLC: 99.6% at 254 nm (t_R = 15.3 min). FT-IR (ATR cm^{-1}): 1034 (C-O), 1149 (S=O), 1249 (S=O), 1345 (C-N), 1589 (C=C), 1618 (C=N), 2838 (-CH_2-), 2934 (-CH_2-), 3041 (C-H_{arom}), 3111 (C-H_{arom}), 3319 (N-H). ^1H NMR (400 MHz, DMSO-d_6) δ ppm: 3.74 (s, 3H, OCH_3), 4.63 (s, 2H, CH_2), 6.94 (d, *J* = 8.7 Hz, 2H, CH_{arom}), 7.33 (d, *J* = 8.5 Hz, 2H, CH_{arom}), 7.77 – 7.87 (m, 2H, CH_{arom}), 7.94 – 8.03 (m, 1H, CH_{arom}), 8.18 – 8.25 (m, 1H, CH_{arom}), 9.87 (s, 1H, NH). ^{13}C (100 MHz, DMSO-d_6) δ ppm: 45.53, 55.10, 113.94, 121.22, 122.99, 127.67, 129.00, 129.25, 133.11, 133.50, 142.23, 158.73, 159.25. HRMS (ESI): *m/z* calcd for $C_{15}H_{15}N_2O_3S$ $[M + H]^+$: 303.0798; found, 303.0790.

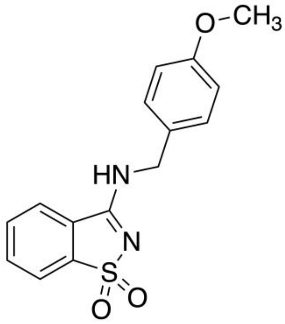

7b

3-((4-chlorobenzyl)amino)benzo[*d*]isothiazole 1,1-dioxide (7c). Product obtained according to the general procedure described in Section 2.1, using amine (**6c**). Reaction time: 60 min (1 h). Column chromatography on silica gel (hexane–ethyl acetate, 4:6). Appearance: white solid. Yield: 99%, 151 mg. m.p. 244–245 °C. HPLC: 98.7% at 254 nm (t_R = 15.9 min). FT-IR (ATR cm^{-1}): 1148 (S=O), 1285 (S=O), 1348 (C-N), 1589 (C=C), 1620 (C=N), 2856 ($-CH_2-$), 2920 ($-CH_2-$), 3065 ($C-H_{arom}$), 3108 ($C-H_{arom}$), 3297 (N-H). ^1H NMR (400 MHz, DMSO-d_6) δ ppm: 4.69 (s, 2H, CH_2), 7.38 – 7.52 (m, 4H, CH_{arom}), 7.79 – 7.93 (m, 2H, CH_{arom}), 7.95 – 8.05 (m, 1H, CH_{arom}), 8.16 – 8.25 (m, 1H, CH_{arom}), 9.94 (s, 1H, NH). ^{13}C (100 MHz, DMSO-d_6) δ ppm: 45.25, 121.27, 123.02, 127.54, 128.50, 129.60, 132.10, 133.17, 133.59, 136.16, 142.20, 159.53. HRMS (ESI): *m/z* calcd for $C_{14}H_{12}ClN_2O_2S$ $[M + H]^+$: 307.0303; found, 307.0290.

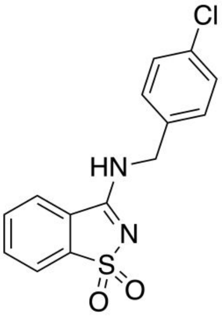

7c

3-(phenethylamino)benzo[*d*]isothiazole 1,1-dioxide (7d). Product obtained according to the general procedure described in Section 2.1, using amine (**6d**). Reaction time: 120 min (2 h). Column chromatography on silica gel (hexane–ethyl acetate, 35:65). Appearance: white solid. Yield: 95%, 136 mg. m.p. 247–248 °C. HPLC: 98.7% at 254 nm (t_R = 17.9 min). FT-IR (ATR cm^{-1}): 1148 (S=O), 1273 (S=O), 1348 (C-N), 1589 (C=C), 1616 (C=N), 2864 ($-CH_2-$), 2936 ($-CH_2-$), 3027 ($C-H_{arom}$), 3106 ($C-H_{arom}$), 3317 (N-H). ^1H NMR (400 MHz, DMSO-d_6) δ ppm: 2.99 (t, *J* = 7.5 Hz, 2H, CH_2), 3.70 (q, J = 7.6 Hz, 2H, CH_2), 7.18 – 7.25 (m, 1H, CH_{arom}), 7.25 – 7.34 (m, 4H, CH_{arom}), 7.79 – 7.85 (m, 2H, CH_{arom}), 7.98 (dd, *J*

= 3.0, 5.7 Hz, 1H, CH_{arom}), 8.15 (dd, *J* = 3.0, 5.7 Hz, 1H, CH_{arom}), 9.53 (s, 1H, NH). ^{13}C (100 MHz, DMSO-d_6) δ ppm: 33.78, 43.99, 121.20, 122.83, 126.37, 127.62, 128.45, 128.64, 133.10, 133.45, 138.61, 142.07, 159.20. HRMS (ESI): *m/z* calcd for $C_{15}H_{15}N_2O_2S$ $[M + H]^+$: 287.0849; found, 287.0857.

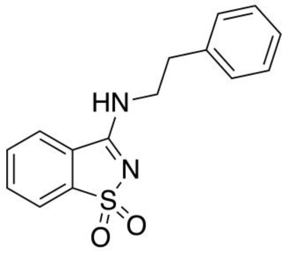

7d

3-((4-methoxyphenethyl)amino)benzo[*d*]isothiazole 1,1-dioxide (7e). Product obtained according to the general procedure described in Section 2.1, using amine (**6e**). Reaction time: 30 min (0.5 h). Column chromatography on silica gel (hexane–ethyl acetate, 35:65). Appearance: white solid. Yield: 92%, 145 mg. m.p. 229–230 °C. HPLC: 99.7% at 254 nm (t_R = 16.2 min). FT-IR (ATR cm^{-1}): 1061 (C-O), 1146 (S=O), 1243 (S=O), 1346 (C-N), 1592 (C=C), 1631 (C=N), 2837 (-CH_2-), 2932 (-CH_2-), 3000-3062 (C-H_{arom}), 3326 (N-H). ^1H NMR (400 MHz, DMSO-d_6) δ ppm: 2.92 (t, *J* = 7.4 Hz, 2H, CH_2), 3.65 (t, *J* = 7.1 Hz, 2H, CH_2), 3.71 (s, 3H, OCH_3), 6.87 (d, *J* = 8.2 Hz, 2H, CH_{arom}), 7.19 (d, *J* = 8.3 Hz, 2H, CH_{arom}), 7.77 – 7.86 (m, 2H, CH_{arom}), 7.93 – 8.06 (m, 1H, CH_{arom}), 8.11 – 8.20 (m, 1H, CH_{arom}), 9.50 (s, 1H, NH). ^{13}C (100 MHz, DMSO-d_6) δ ppm: 32.93, 44.28, 54.97, 113.88, 121.21, 122.84, 127.65, 129.63, 130.44, 133.10, 133.45, 142.07, 157.87, 159.19. HRMS (ESI): *m/z* calcd for $C_{16}H_{17}N_2O_3S$ $[M + H]^+$: 317.0954; found, 317.0955.

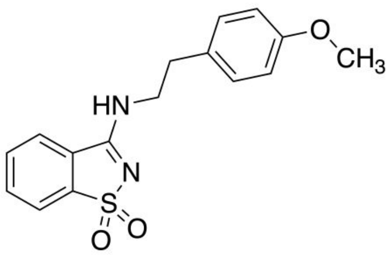

7e

3-((4-chlorophenethyl)amino)benzo[*d*]isothiazole 1,1-dioxide (7f). Product obtained according to the general procedure described in Section 2.1, using amine (**6f**). Reaction time: 5 min (0.08 h). Column chromatography on silica gel (hexane–ethyl acetate, 35:65). Appearance: white solid. Yield: 80%, 128 mg. m.p. 231–232 °C. HPLC: 98.1% at 254 nm (t_R = 16.6 min). FT-IR (ATR cm^{-1}): 1148 (S=O), 1272 (S=O), 1349 (C-N), 1591 (C=C), 1627 (C=N), 2874 (-CH_2-), 2940 (-CH_2-), 3062 (C-H_{arom}), 3108 (C-

H_{arom}), 3293 (N-H). ^{1}H NMR (400 MHz, DMSO-d_6) δ ppm: 2.98 (t, *J* = 7.3 Hz, 2H, CH_2), 3.69 (t, *J* = 7.2 Hz, 2H, CH_2), 7.30 (d, *J* = 8.7 Hz, 2H, CH_{arom}), 7.35 (d, *J* = 8.5 Hz, 2H, CH_{arom}), 7.77 – 7.86 (m, 2H, CH_{arom}), 7.97 (dd, *J* = 3.3, 5.4 Hz, 1H, CH_{arom}), 8.14 (dd, *J* = 3.0, 5.7 Hz, 1H, CH_{arom}), 9.49 (s, 1H, NH). ^{13}C (100 MHz, DMSO-d_6) δ ppm: 33.04, 43.74, 121.21, 122.83, 127.57, 128.34, 130.56, 131.05, 133.10, 133.46, 137.65, 142.05, 159.24. HRMS (ESI): *m/z* calcd for $C_{15}H_{14}ClN_2O_2S$ [M + H]$^+$: 321.0459; found, 321.0459.

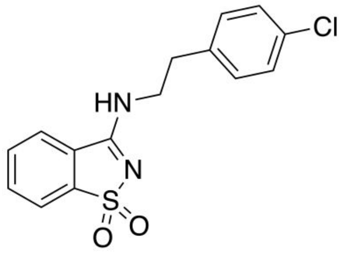

7f

3-((3-phenylpropyl)amino)benzo[*d*]isothiazole 1,1-dioxide (7g). Product obtained according to the general procedure described in Section 2.1, using amine (**6g**). Reaction time: 180 min (3 h). Column chromatography on silica gel (hexane–ethyl acetate, 3:7). Appearance: white solid. Yield: 80%, 120 mg. m.p. 180 °C. HPLC: 99.9% at 254 nm (t_R = 17.1 min). FT-IR (ATR cm^{-1}): 1148 (S=O), 1272 (S=O), 1346 (C-N), 1593 (C=C), 1624 (C=N), 2857 (-CH_2-), 2920 (-CH_2-), 3027-3115 (C-H_{arom}), 3353 (N-H). ^{1}H NMR (400 MHz, DMSO-d_6) δ ppm: 1.97 (p, *J* = 7.4 Hz, 2H, CH_2), 2.69 (t, *J* = 7.6 Hz, 2H, CH_2), 3.49 (t, *J* = 7.2 Hz, 2H, CH_2), 7.18 (t, *J* = 7.0 Hz, 1H, CH_{arom}), 7.22 – 7.32 (m, 4H, CH_{arom}), 7.79 – 7.87 (m, 2H, CH_{arom}), 7.93 – 8.03 (m, 1H, CH_{arom}), 8.14 – 8.22 (m, 1H, CH_{arom}), 9.41 (s, 1H, NH). ^{13}C (100 MHz, DMSO-d_6) δ ppm: 29.69, 32.39, 42.28, 121.14, 122.89, 125.84, 127.69, 128.29, 133.00, 133.38, 141.25, 142.13, 159.23. HRMS (ESI): *m/z* calcd for $C_{16}H_{17}N_2O_2S$ [M + H]$^+$: 301.1005; found, 301.1001.

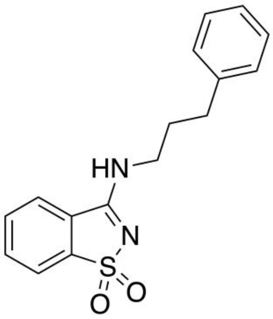

7g

3-((3-(4-methoxyphenyl)propyl)amino)benzo[*d*]isothiazole 1,1-dioxide (7h). Product obtained according to the general procedure described in Section 2.1, with minor modifications. Four

equivalents of HMDS (2.0 mmol, 4.0 equiv.) were used due to the amine (**6h**) being in its salt form. Reaction time: 60 min (1 h). Column chromatography on silica gel (hexane–ethyl acetate, 3:7). Appearance: white solid. Yield: 76%, 125 mg. m.p. 158–159 °C. HPLC: 99.8% at 254 nm (t_R = 16.3 min). FT-IR (ATR cm^{-1}): 1041 (C-O), 1152 (S=O), 1274 (S=O), 1351 (C-N), 1592 (C=C), 1619 (C=N), 2834 ($-CH_2-$), 2921 ($-CH_2-$), 3046 ($C-H_{arom}$), 3111 ($C-H_{arom}$), 3353 (N-H). ^1H NMR (400 MHz, DMSO-d_6) δ ppm: 1.93 (p, *J* = 7.4 Hz, 2H, CH_2), 2.62 (t, *J* = 7.6 Hz, 2H, CH_2), 3.47 (t, *J* = 7.2 Hz, 2H, CH_2), 3.71 (s, 3H, OCH_3), 6.84 (d, *J* = 8.9 Hz, 2H, CH_{arom}), 7.15 (d, *J* = 8.5 Hz, 2H, CH_{arom}), 7.77 – 7.88 (m, 2H, CH_{arom}), 7.92 – 8.01 (m, 1H, CH_{arom}), 8.12 – 8.21 (m, 1H, CH_{arom}), 9.36 (s, 1H, NH). ^{13}C (100 MHz, DMSO-d_6) δ ppm: 29.93, 31.52, 42.27, 54.92, 113.72, 121.17, 122.91, 127.71, 129.24, 133.01, 133.06, 133.40, 142.13, 157.47, 159.22. HRMS (ESI): *m/z* calcd for $C_{17}H_{19}N_2O_3S$ $[M + H]^+$: 331.1111; found, 331.1112.

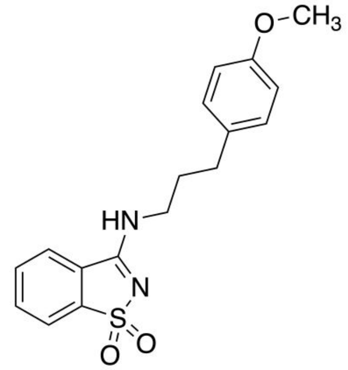

7h

3-((3-(4-chlorophenyl)propyl)amino)benzo[*d*]isothiazole 1,1-dioxide (7i). Product obtained according to the general procedure described in Section 2.1, with minor modifications. Four equivalents of HMDS (2.0 mmol, 4.0 equiv.) were used due to the amine (**6i**) being in its salt form. Reaction time: 120 min (2 h). Column chromatography on silica gel (hexane–ethyl acetate, 3:7). Appearance: white solid. Yield: 70%, 117 mg. m.p. 203–205 °C. HPLC: 96.7% at 254 nm (t_R = 16.9 min). FT-IR (ATR cm^{-1}): 1151 (S=O), 1272 (S=O), 1353 (C-N), 1591 (C=C), 1621 (C=N), 2922 ($C-H_{sp3}$), 3050 ($C-H_{arom}$), 3330 (N-H). ^1H NMR (400 MHz, DMSO-d_6) δ ppm: 1.95 (p, *J* = 7.5 Hz, 2H, CH_2), 2.68 (t, *J* = 7.6 Hz, 2H, CH_2), 3.48 (q, *J* = 6.6 Hz, 2H, CH_2), 7.27 (d, *J* = 8.2 Hz, 2H, CH_{arom}), 7.32 (d, *J* = 8.2 Hz, 2H, CH_{arom}), 7.77 – 7.87 (m, 2H, CH_{arom}), 7.93 – 8.00 (m, 1H, CH_{arom}), 8.11 – 8.20 (m, 1H, CH_{arom}), 9.39 (s, 1H, NH). ^{13}C (100 MHz, DMSO-d_6) δ ppm: 29.50, 31.67, 42.17, 121.14, 122.87, 127.67, 128.19, 130.18, 130.46, 132.99, 133.38, 140.28, 142.11, 159.24. HRMS (ESI): *m/z* calcd for $C_{16}H_{16}ClN_2O_2S$ $[M + H]^+$: 335.0616; found, 335.0611.

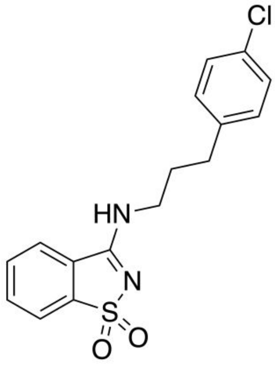

7i

3-(propylamino)benzo[*d*]isothiazole 1,1-dioxide (7j). Product obtained according to the general procedure described in Section 2.1, using amine (**6j**). Reaction time: 120 min (2 h). Column chromatography on silica gel (hexane–ethyl acetate, 25:75). Appearance: white solid. Yield: 96%, 107 mg. m.p. 202–203 °C. HPLC: 98.7% at 254 nm (t_R = 15.0 min). FT-IR (ATR cm^{-1}): 1149 (S=O), 1273 (S=O), 1344 (C-N), 1591 (C=C), 1622 (C=N), 2878 (C-H_{sp3}), 2935 (C-H_{sp3}), 2967 (C-H_{sp3}), 3034 (C-H_{arom}), 3110 (C-H_{arom}), 3325 (N-H). ^1H NMR (400 MHz, DMSO-d_6) δ ppm: 0.94 (t, J = 7.4 Hz, 3H, CH_3), 1.66 (sx, J = 7.5 Hz, 2H, CH_2), 3.43 (t, J = 7.4 Hz, 2H, CH_2), 7.77 – 7.89 (m, 2H, CH_{arom}), 7.92 – 8.00 (m, 1H, CH_{arom}), 8.15 – 8.22 (m, 1H, CH_{arom}), 9.39 (s, 1H, NH). ^{13}C (100 MHz, DMSO-d_6) δ ppm: 11.30, 21.40, 44.33, 121.14, 122.85, 127.71, 133.01, 133.37, 142.15, 159.20. HRMS (ESI): m/z calcd for $C_{10}H_{13}N_2O_2S$ $[M + H]^+$: 225.0692; found, 225.0695.

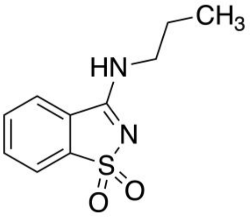

7j

3-((2-hydroxyethyl)amino)benzo[*d*]isothiazole 1,1-dioxide (7k). Product obtained according to the general procedure described in Section 2.1, using amine (**6k**), with additional steps applied. After starting material consumption (25 min, 0.42 h), a mixture of 5.0 mL methanol (125 mmol, 250 equiv.) and 0.55 mL distilled water (30 mmol, 60 equiv.) was added to the reaction and stirred for 60 min (1 h). The material was transferred to a 100 mL round-bottom flask and concentrated using a rotary evaporator. Reaction time: 25 min (0.42 h) for reaction + 60 min (1 h) for trimethylsilyl group removal. Column chromatography on silica gel (ethyl acetate, 100%). Appearance: white solid. Yield: 71%, 80 mg. m.p. 239–240 °C. HPLC: 99.9% at 254 nm (t_R = 12.9 min). FT-IR (ATR cm^{-1}): 1146 (S=O),

1277 (S=O), 1360 (C-N), 1590 (C=C), 1624 (C=N), 2888 (-CH_2-), 2942 (-CH_2-), 3037 (C-H_{arom}), 3094 (C-H_{arom}), 3228 (N-H), 3409 (O-H). ^{1}H NMR (400 MHz, DMSO-d_6) δ ppm: 3.50 – 3.58 (m, 2H, CH_2), 3.65 (t, *J* = 5.7 Hz, 2H, CH_2), 5.01 (s, 1H, OH), 7.77 – 7.89 (m, 2H, CH_{arom}), 7.92 – 8.04 (m, 1H, CH_{arom}), 8.17 – 8.29 (m, 1H, CH_{arom}), 9.50 (s, 1H, NH). ^{13}C (100 MHz, DMSO-d_6) δ ppm: 45.53, 58.47, 121.13, 123.05, 127.79, 133.03, 133.39, 142.14, 159.52. HRMS (ESI): *m/z* calcd for $C_9H_{11}N_2O_3S$ [M + H]$^+$: 227.0485; found, 227.0487.

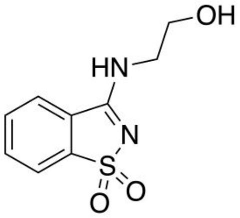

7k

3-((cyclohexylmethyl)amino)benzo[*d*]isothiazole 1,1-dioxide (7l). Product obtained according to the general procedure described in Section 2.1, using amine (**6l**). Reaction time: 20 min (0.33 h). Column chromatography on silica gel (hexane–ethyl acetate, 35:65). Appearance: white solid. Yield: 96%, 133 mg. m.p. 219–220 °C. HPLC: 99.0% at 254 nm (t_R = 16.6 min). FT-IR (ATR cm^{-1}): 1150 (S=O), 1275 (S=O), 1347 (C-N), 1592 (C=C), 1621 (C=N), 2850 (C-H_{sp3}), 2925 (C-H_{sp3}), 3047-3113 (C-H_{arom}), 3296 (N-H). ^{1}H NMR (400 MHz, DMSO-d_6) δ ppm: 0.91 – 1.05 (m, 2H, $CH_{2[cyclohexane]}$), 1.10 – 1.28 (m, 4H, $CH_{2[cyclohexane]}$), 1.58 – 1.65 (m, 1H, $CH_{[cyclohexane]}$), 1.66 – 1.79 (m, 4H, $CH_{2[cyclohexane]}$), 3.28 – 3.37 (m, 2H, CH_2), 7.77 – 7.86 (m, 2H, CH_{arom}), 7.96 (dd, *J* = 2.7, 6.0 Hz, 1H, CH_{arom}), 8.21 (dd, *J* = 2.8, 5.8 Hz, 1H, CH_{arom}), 9.38 (s, 1H, NH). ^{13}C (100 MHz, DMSO-d_6) δ ppm: 25.24, 25.84, 30.31, 36.66, 48.77, 121.12, 122.90, 127.69, 132.98, 133.35, 142.18, 159.38. HRMS (ESI): *m/z* calcd for $C_{14}H_{19}N_2O_2S$ [M + H]$^+$: 279.1162; found, 279.1156.

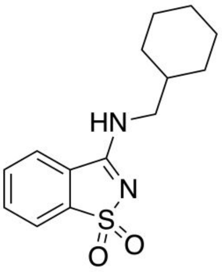

7l

3-((2-(thiophen-2-yl)ethyl)amino)benzo[*d*]isothiazole 1,1-dioxide (7m). Product obtained according to the general procedure described in Section 2.1, using amine (**6m**). Reaction time: 10

min (0.16 h). Column chromatography on silica gel (hexane–ethyl acetate, 35:65). Appearance: white solid. Yield: 71%, 103 mg. m.p. 234–235 °C. HPLC: 99.2% at 254 nm (t_R = 16.0 min). FT-IR (ATR cm^{-1}): 1149 (S=O), 1273 (S=O), 1349 (C-N), 1592 (C=C), 1621 (C=N), 2856 (-CH_2-), 2924 (-CH_2-), 3045 (C-H_{arom}), 3105 (C-H_{sp2}) 3334 (N-H). ^{1}H NMR (400 MHz, DMSO-d_6) δ ppm: 3.22 (t, J = 7.2 Hz, 2H, CH_2), 3.72 (q, J = 6.6 Hz, 2H, CH_2), 6.94 – 6.99 (m, 2H, $CH_{[thiophene]}$), 7.33 – 7.39 (m, 1H, $CH_{[thiophene]}$), 7.80 – 7.86 (m, 2H, CH_{arom}), 7.95 – 8.02 (m, 1H, CH_{arom}), 8.12 – 8.19 (m, 1H, CH_{arom}), 9.57 (t, J = 5.5 Hz, 1H, NH). ^{13}C (100 MHz, DMSO-d_6) δ ppm: 27.95, 44.02, 121.23, 122.84, 124.40, 125.54, 127.04, 127.59, 133.14, 133.50, 140.52, 142.04, 159.29. HRMS (ESI): *m/z* calcd for $C_{13}H_{13}N_2O_2S_2$ [M + H]$^+$: 293.0413; found, 293.0412.

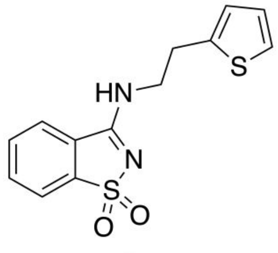

7m

3-((2-(piperidin-1-yl)ethyl)amino)benzo[*d*]isothiazole 1,1-dioxide (7n). Product obtained according to the general procedure described in Section 2.1, using amine (**6n**). Reaction time: 15 min (0.25 h). Column chromatography on silica gel (methanol, 100%). Appearance: white solid. Yield: 99%, 145 mg. m.p. 169–170 °C. HPLC: 99.8% at 254 nm (t_R = 12.6 min). FT-IR (ATR cm^{-1}): 1150 (S=O), 1279 (S=O), 1354 (C-N), 1590 (C=C), 1617 (C=N), 2738-2936 (C-H_{sp3}), 3048 (C-H_{arom}), 3306 (N-H). ^{1}H NMR (400 MHz, DMSO-d_6) δ ppm: 1.35 (p, J = 5.7 Hz, 2H, $CH_{2[piperidine]}$), 1.42 – 1.52 (m, 4H, $CH_{2[piperidine]}$), 2.36 – 2.42 (m, 4H, $CH_{2[piperidine]}$), 2.54 (t, J = 6.8 Hz, 2H, CH_2), 3.56 (t, J = 6.8 Hz, 2H, CH_2), 7.77 – 7.85 (m, 2H, CH_{arom}), 7.91 – 8.02 (m, 1H, CH_{arom}), 8.22 (dt, J = 3.4, 5.5 Hz, 1H, CH_{arom}). ^{13}C (100 MHz, DMSO-d_6) δ ppm: 23.94, 25.48, 40.32, 54.01, 56.57, 121.11, 122.98, 127.73, 132.98, 133.35, 142.18, 159.29. HRMS (ESI): *m/z* calcd for $C_{14}H_{20}N_3O_2S$ [M + H]$^+$: 294.1271; found, 294.1280.

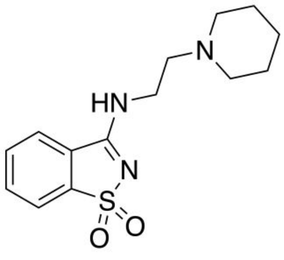

7n

3-((3-(piperidin-1-yl)propyl)amino)benzo[*d*]isothiazole 1,1-dioxide (7o). Product obtained according to the general procedure described in Section 2.1, using amine (**6o**). Reaction time: 20 min (0.33 h). Column chromatography on silica gel (methanol, 100%). Appearance: white solid. Yield: 74%, 113 mg. m.p. 174–175 °C. HPLC: 98.9% at 254 nm (t_R = 11.8 min). FT-IR (ATR cm^{-1}): 1161 (S=O), 1301 (S=O), 1346 (C-N), 1585 (C=C), 1619 (C=N), 2824-2932 (C-H_{sp3}), 3080 (C-H_{arom}), 3185 (N-H). ^1H NMR (400 MHz, DMSO-d_6) δ ppm: 1.36 (p, *J* = 5.8 Hz, 2H, $CH_{2[piperidine]}$), 1.47 (p, *J* = 5.6 Hz, 4H, $CH_{2[piperidine]}$), 1.79 (p, *J* = 7.1 Hz, 2H, CH_2), 2.27 – 2.35 (m, 6H, $CH_{2[piperidine + propyl chain]}$), 3.47 (t, *J* = 7.1 Hz, 2H, CH_2), 7.77 – 7.85 (m, 2H, CH_{arom}), 7.95 (dd, *J* = 2.7, 6.0 Hz, 1H, CH_{arom}), 8.16 (dd, *J* = 2.8, 5.8 Hz, 1H, CH_{arom}). ^{13}C (100 MHz, DMSO-d_6) δ ppm: 24.12, 25.38, 25.58, 41.39, 54.02, 55.99, 121.09, 122.81, 127.92, 127.95, 132.93, 133.26, 142.16, 159.06. HRMS (ESI): *m/z* calcd for $C_{15}H_{22}N_3O_2S$ [M + H]$^+$: 308.1427; found, 308.1439.

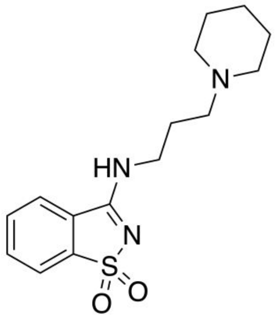

7o

3-(cyclopentylamino)benzo[*d*]isothiazole 1,1-dioxide (7p). Product obtained according to the general procedure described in Section 2.1, using amine (**6p**). Reaction time: 35 min (0.58 h). Column chromatography on silica gel (hexane–ethyl acetate, 35:65). Appearance: white solid. Yield: 91%, 113 mg. m.p. 230–231 °C. HPLC: 96.2% at 254 nm (t_R = 15.8 min). FT-IR (ATR cm^{-1}): 1151 (S=O), 1275 (S=O), 1341 (C-N), 1590 (C=C), 1616 (C=N), 2871 (C-H_{sp3}), 2952 (C-H_{sp3}), 3101 (C-H_{arom}), 3326 (N-H). ^1H NMR (400 MHz, DMSO-d_6) δ ppm: 1.57 – 1.80 (m, 6H, $CH_{2[cyclopentyl]}$), 1.97 – 2.11 (m, 2H, $CH_{2[cyclopentyl]}$), 4.24 – 4.37 (m, 1H, $CH_{[cyclopentyl]}$), 7.77 – 7.84 (m, 2H, CH_{arom}), 7.89 – 7.95 (m, 1H, CH_{arom}), 8.21 – 8.29 (m, 1H, CH_{arom}), 9.02 (s, 1H, NH). ^{13}C (100 MHz, DMSO-d_6) δ ppm: 23.44, 31.47, 54.38, 120.73, 122.84, 127.56, 132.48, 132.96, 142.06, 158.43. HRMS (ESI): *m/z* calcd for $C_{12}H_{15}N_2O_2S$ [M + H]$^+$: 251.0849; found, 251.0841.

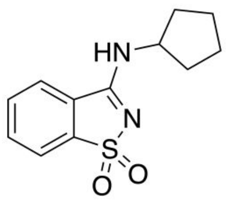

7p

3-((2,3-dihydro-1*H*-inden-2-yl)amino)benzo[*d*]isothiazole 1,1-dioxide (7q). Product obtained according to the general procedure described in Section 2.1, with minor modifications. Four equivalents of HMDS (2.0 mmol, 4.0 equiv.) were used due to the amine (**6q**) being in its salt form. Reaction time: 240 min (4 h). Column chromatography on silica gel (hexane–ethyl acetate, 2:8), followed by recrystallization from acetone*. Appearance: white solid. Yield: 43%, 64 mg (*31%, 19 mg). m.p. >300 °C. HPLC: 98.3% at 254 nm (t_R = 16.4 min). FT-IR (ATR cm^{-1}): 1149 (S=O), 1271 (S=O), 1379 (C-N), 1590 (C=C), 1615 (C=N), 2848 (C-H_{sp3}), 2937 (C-H_{sp3}), 3027 (C-H_{arom}), 3103 (C-H_{arom}), 3322 (N-H). ^{1}H NMR (400 MHz, DMSO-d_6) δ ppm: 3.12 (dd, *J* = 5.6, 16.3 Hz, 2H, $CH_{2[indane]}$), 3.42 (dd, *J* = 7.8, 16.2 Hz, 2H, $CH_{2[indane]}$), 4.73 – 4.86 (m, 1H, $CH_{[indane]}$), 7.15 – 7.24 (m, 2H, CH_{arom}), 7.26 – 7.32 (m, 2H, CH_{arom}), 7.75 – 7.88 (m, 2H, CH_{arom}), 7.91 – 7.98 (m, 1H, CH_{arom}), 8.21 – 8.29 (m, 1H, CH_{arom}), 9.39 (d, *J* = 6.8 Hz, 1H, NH). ^{13}C (100 MHz, DMSO-d_6) δ ppm: 38.32, 53.47, 120.79, 122.94, 124.24, 126.34, 127.44, 132.54, 133.06, 140.35, 142.02, 158.74. HRMS (ESI): *m/z* calcd for $C_{16}H_{15}N_2O_2S$ [M + H]$^+$: 299.0849; found, 299.0836.

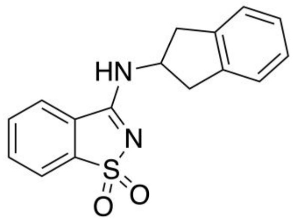

7q

3-(phenylamino)benzo[*d*]isothiazole 1,1-dioxide (7r). Product obtained according to the general procedure described in Section 2.1, using amine (**6r**). Reaction time: 24 h. Column chromatography on silica gel (hexane–ethyl acetate, 1:1). Appearance: white solid. Yield: 55%, 71 mg. m.p. >300 °C. HPLC: 97.2% at 254 nm (t_R = 15.9 min). FT-IR (ATR cm^{-1}): 1151 (S=O), 1271 (S=O), 1352 (C-N), 1563 (C=C), 1614 (C=N), 3055 (C-H_{arom}), 3107 (C-H_{arom}), 3325 (N-H). ^{1}H NMR (400 MHz, DMSO-d_6) δ ppm: 7.27 (t, *J* = 7.4 Hz, 1H, CH_{arom}), 7.49 (t, *J* = 7.7 Hz, 2H, CH_{arom}), 7.82 – 7.96 (m, 4H, CH_{arom}), 8.07 (d, *J* = 7.1 Hz, 1H, CH_{arom}), 8.49 (d, *J* = 7.3 Hz, 1H, CH_{arom}), 10.86 (s, 1H, NH). ^{13}C (100 MHz, DMSO-d_6) δ ppm:

121.45, 122.07, 123.56, 125.66, 128.24, 129.01, 133.29, 133.69, 137.43, 140.75, 156.75. HRMS (ESI): *m/z* calcd for $C_{13}N_{11}N_2O_2S$ $[M + H]^+$: 259.0536; found, 259.0528.

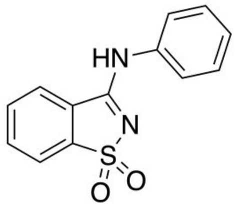

7r

3-(pyrrolidin-1-yl)benzo[*d*]isothiazole 1,1-dioxide (7s). Product obtained according to the general procedure described in Section 2.1, using amine (**6s**). Reaction time: 30 min (0.5 h). Column chromatography on silica gel (hexane–ethyl acetate, 15:85). Appearance: light brown solid. Yield: 97%, 114 mg. m.p. 260–262 °C. HPLC: 99.0% at 254 nm (t_R = 14.6 min). FT-IR (ATR cm^{-1}): 1156 (S=O), 1282 (S=O), 1345 (C-N), 1590 (C=C), 1607 (C=N), 2875 (CH_{sp3}), 2922 (CH_{sp3}), 2960 (CH_{sp3}), 3002 (C-H_{arom}), 3063 (C-H_{arom}). ^1H NMR (400 MHz, DMSO-d_6) δ ppm: 1.92 (p, *J* = 6.8 Hz, 2H, $CH_{2[pyrrolidine]}$), 2.09 (p, *J* = 6.8 Hz, 2H, $CH_{2[pyrrolidine]}$), 3.69 (t, *J* = 6.9 Hz, 2H, $CH_{2[pyrrolidine]}$), 4.08 (t, *J* = 6.9 Hz, 1H, $CH_{2[pyrrolidine]}$), 7.75 – 7.89 (m, 2H, CH_{arom}), 7.99 (d, *J* = 7.1 Hz, 1H, CH_{arom}), 8.12 (d, *J* = 7.5 Hz, 1H, CH_{arom}). ^{13}C (100 MHz, DMSO-d_6) δ ppm: 23.27, 26.10, 49.13, 51.50, 121.73, 126.43, 127.57, 133.03, 133.09, 143.67, 157.55. HRMS (ESI): *m/z* calcd for $C_{11}H_{13}N_2O_2S$ $[M + H]^+$: 237.0692; found, 237.0692.

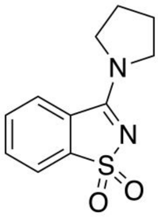

7s

3-(3,4-dihydroisoquinolin-2(1*H*)-yl)benzo[*d*]isothiazole 1,1-dioxide (7t). Product obtained according to the general procedure described in Section 2.1, using amine (**6t**), with modifications. The crude solid was washed with a mixture of acetonitrile–methanol (1:1) and filtered. Reaction time: 120 min (2 h). The compound was purified by washing with a mixture of acetonitrile–methanol (1:1). Appearance: white solid. Yield: 43%, 64 mg. m.p. 254–256 °C. HPLC: 98.9% at 254 nm (t_R = 16.3 min). FT-IR (ATR cm^{-1}): 1155 (S=O), 1289 (S=O), 1362 (C-N), 1562 (C=C), 1598 (C=N), 2842 (CH_{sp3}), 2939 (CH_{sp3}), 3069 (C-H_{arom}). ^1H NMR (400 MHz, DMSO-d_6) δ ppm: 3.09 (s, 2H, CH_2), 4.19 – 4.23 (m, 2H, CH_2), 4.99 – 5.03 (m, 2H, CH_2), 7.22 – 7.34 (m, 3H, CH_{arom}), 7.34 – 7.42 (m, 1H, CH_{arom}), 7.78 –

7.89 (m, 2H, CH_{arom}), 7.96 – 8.05 (m, 1H, CH_{arom}), 8.28 – 8.33 (m, 1H, CH_{arom}). ^{13}C (100 MHz, DMSO-d_6) δ ppm: 27.81, 45.96, 49.16, 121.61, 126.27, 126.34, 126.97, 127.25, 127.39, 132.77, 132.87, 134.75, 143.93, 159.74. HRMS (ESI): *m/z* calcd for $C_{16}H_{15}N_2O_2S$ $[M + H]^+$: 299.0849; found, 299.0841.

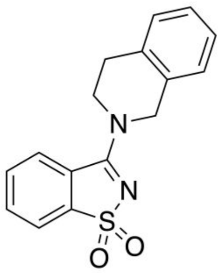

7t

2.2. Isolation and characterization of a putative tetrahedral adduct (7c-adduct) from the reaction leading to 7c

In a sealed 10 mL Schlenk tube, saccharin (**1**) (0.5 mmol, 1.0 equiv.), 4-chlorobenzylamine (**6c**) (0.7 mmol, 1.4 equiv.), hexamethyldisilazane (HMDS) (0.7 mmol, 1.4 equiv.), and ammonium sulfate ($(NH_4)_2SO_4$) (0.05 mmol, 0.1 equiv.) were heated at 125 °C (**Scheme S1**). The reaction progress was monitored by TLC using a hexane–ethyl acetate (4:6) mixture as eluent. After 35 min, the material was solubilized, transferred to a 100 mL round-bottom flask, and concentrated using a rotary evaporator. The crude solid was purified by column chromatography using a hexane and ethyl acetate (4:6) mixture under isocratic conditions. The fractions containing the desired product (**7c**) and the putative tetrahedral adduct (**7c-adduct**) were concentrated separately.

Scheme S2. Formation of (7c) and a putative tetrahedral adduct (7c-adduct) upon premature interruption of the reaction.[a]

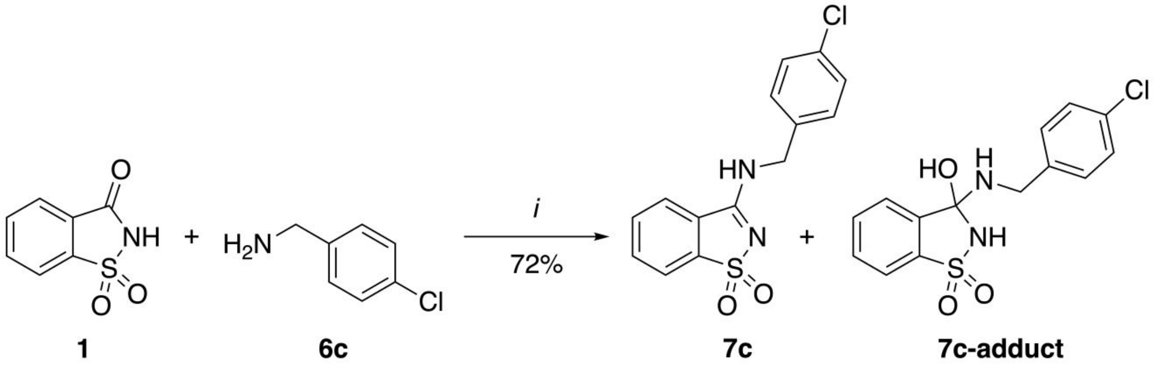

[a]Conditions and reagents are as follows: *i*) HMDS, $(NH_4)_2SO_4$, 125 °C, 35 min, 72% (**7c**)[1].

3-((4-chlorobenzyl)amino)-3-hydroxy-2,3-dihydrobenzo[*d*]isothiazole 1,1-dioxide (7c-adduct). Compound obtained according to the procedure described above, using amine (**6c**). Reaction time: 35 min (0.58 h). Column chromatography on silica gel (hexane–ethyl acetate, 4:6). Appearance: white solid. ^{1}H NMR (400 MHz, DMSO-d$_6$) δ ppm: 4.48 (d, *J* = 5.9 Hz, 2H, CH_2), 7.15 (s, 2H), 7.36 – 7.47 (m, 4H), 7.58 – 7.64 (m, 1H), 7.65 – 7.74 (m, 2H), 7.92 – 7.99 (m, 1H), 9.31 (t, *J* = 6.0 Hz, 1H). ^{13}C (100 MHz, DMSO-d$_6$) δ ppm: 41.98, 127.04, 128.18, 129.14, 129.17, 130.26, 131.44, 132.19, 134.61, 137.87, 140.88, 168.80. HRMS (ESI): *m/z* calcd for $C_{14}H_{14}ClN_2O_3S$ $[M + H]^+$: 325.0408; found, 325.0403.

[1] The spectroscopic, spectrometric, and physical data were consistent with those reported in Section 2.1.

2.3. Attempted silylation–amination of benzo[*d*]isothiazol-3(2*H*)-one (8) using the developed protocol

The applicability of the developed protocol (see **Section 2.1**) was also explored using benzo[*d*]isothiazol-3(2*H*)-one (**8**) as the five-membered hydroxy *N*-heterocycle (**Scheme S3**). Under the optimized conditions, the limiting reagent **8** was treated with phenethylamine (**6d**). The reaction was monitored up to 24 hours, and no conversion was observed.

Scheme S3. Attempted silylation–amination of benzo[*d*]isothiazol-3(2*H*)-one (8) under the developed protocol conditions.[a]

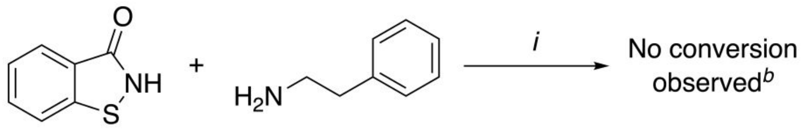

8 **6d**

[a]Conditions and reagents are as follows: *i*) HMDS, $(NH_4)_2SO_4$, 125 °C, 24 h.

[b]Based on TLC and HPLC analysis.

It is worth emphasizing that the reaction did not provide the desired product under the conditions of the developed protocol. Therefore, it is premature to conclude that silylation–amination does not occur in heterocycle **8** (**Scheme S3**), since other experimental conditions were not evaluated.

Notably, the SO_2 moiety appears to play an important role in the silylation–amination of saccharin (**1**) (**Scheme S1**), as the SO_2 oxygen atoms are absent in compound **8**. Previous studies have successfully applied silylation–amination to the five-membered hydroxy *N*-heterocycle 2-hydroxyindole, but not to the silylated benzo[*d*]oxazol-2-ol system.[1] Collectively, these findings suggest that five-membered hydroxy *N*-heterocycles may require specific conditions and/or structural features for an effective silylation–amination process. Further studies are needed to better understand the applicability of this transformation to such five-membered systems.

2.4. Scale-up of 3-(phenethylamino)benzo[*d*]isothiazole 1,1-dioxide (7d)

In a sealed 10 mL Schlenk tube, saccharin (**1**) (5.0 mmol, 1.0 equiv.), phenethylamine (**6d**) (7.0 mmol, 1.4 equiv.), hexamethyldisilazane (HMDS) (7.0 mmol, 1.4 equiv.), and ammonium sulfate ($(NH_4)_2SO_4$) (0.5 mmol, 0.1 equiv.) were heated at 125 °C (**Scheme S4**). The reaction progress was monitored by TLC using a hexane–ethyl acetate (35:65) mixture as eluent. Following reaction completion, the material was solubilized, transferred to a 100 mL round-bottom flask, and concentrated using a rotary evaporator. The crude solid was washed with water and filtered. Subsequently, the resulting filtered solid was recrystallized from acetonitrile.

Scheme S4. Synthesis of 3-(phenethylamino)benzo[*d*]isothiazole 1,1-dioxide (7d)[a]

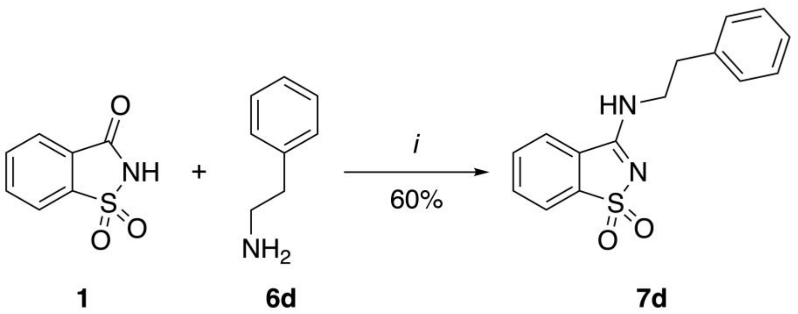

[a]Conditions and reagents are as follows: *i*) HMDS, $(NH_4)_2SO_4$, 125 °C, 2 h.

3-(phenethylamino)benzo[*d*]isothiazole 1,1-dioxide (7d). Product obtained according to the procedure described above, using amine (**6d**). Reaction time: 120 min (2 h). Recrystallization from acetonitrile. Appearance: white crystals. Yield: 60%, 859 mg (several crops). HPLC: 99.5% at 254 nm (t_R = 17.0 min). The spectroscopic, spectrometric, and physical data were consistent with those reported in Section 2.1.

2.5. Experimental notes

This section provides practical observations and considerations regarding the developed methodology and the silylation–amination process.

- It is recommended to use a Schlenk tube with a larger neck diameter to facilitate the addition of the reaction components.
- Although no strict order of addition is required, the following sequence was adopted: (i) ammonium sulfate ($(NH_4)_2SO_4$), to ensure complete transfer and visual confirmation; (ii) saccharin (**1**) (**Scheme S1**); (iii) the amine, if solid; and (iv) for liquid amines, the component with the lower boiling point (amine or HMDS) was added first.
- Silylation–amination can be performed in a round-bottom flask under reflux.[1,2] However, the use of a Schlenk tube is recommended when the amine has a boiling point below 125 °C, is supplied as a salt, or when its physical properties (boiling and/or melting point) are not specified by the supplier.
- Since the reaction is performed under solvent-free conditions, the system is initially in the solid state. Upon heating, the mixture normally becomes liquid. Bubble formation is typically observed during this initial stage, which is indicative of ammonia (NH_3) evolution.[1]
- Some reactions undergo a phase transition from liquid to a predominantly solid mixture, which may indicate product formation. However, this does not guarantee complete consumption of the limiting reagent. Even when the reaction mixture appears largely solid, additional time may be required to achieve full conversion. The reaction leading to 3-((4-chlorobenzyl)amino)benzo[*d*]isothiazole 1,1-dioxide (**7c**) (**Section 2.1**) serves as a practical example. Interestingly, this initial misinterpretation ultimately led to the isolation and characterization of the putative tetrahedral adduct (**7c-adduct**) (**Section 2.2**).
- As observed for 3-((2-hydroxyethyl)amino)benzo[*d*]isothiazole 1,1-dioxide (**7k**), reactants bearing additional hydroxyl or amino functionalities may undergo silylation upon HMDS treatment. The putative silylated analogue of **7k** was detected by TLC analysis, showing a higher R_f value compared with product **7k** obtained after treatment with a methanol–water mixture. This difference in polarity may also occur in other cases and facilitate monitoring of the conversion.

- Purification by column chromatography is relatively straightforward. As observed for *N*-phenethylquinazolin-4-amines,[2] silylation–amination using **1** also leads to the formation of fluorescent byproducts. Some of these are not detected by TLC (254/360 nm), possibly due to their low concentration, and are concentrated in fractions eluting prior to those corresponding to the desired product.
- Recrystallization represents a potential alternative to column chromatography for compounds with low solubility and may be further explored by testing different solvent systems to optimize recovery and purity.
- For additional examples of silylation–amination in related five- and six-membered systems, see refs[1–7].

3. ANALYTICAL DATA

3.1. ^{1}H and ^{13}C NMR spectra of the synthesized compounds

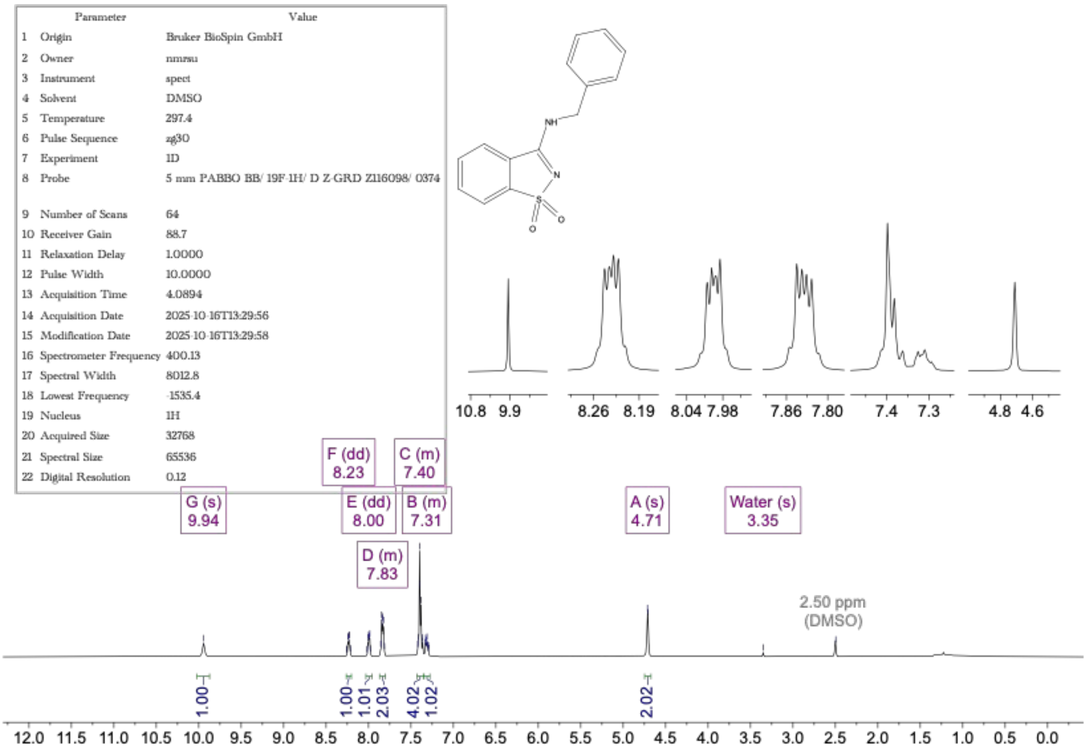

Figure S1. ^{1}H NMR spectrum (400 MHz, DMSO-d_6) of compound **7a**.

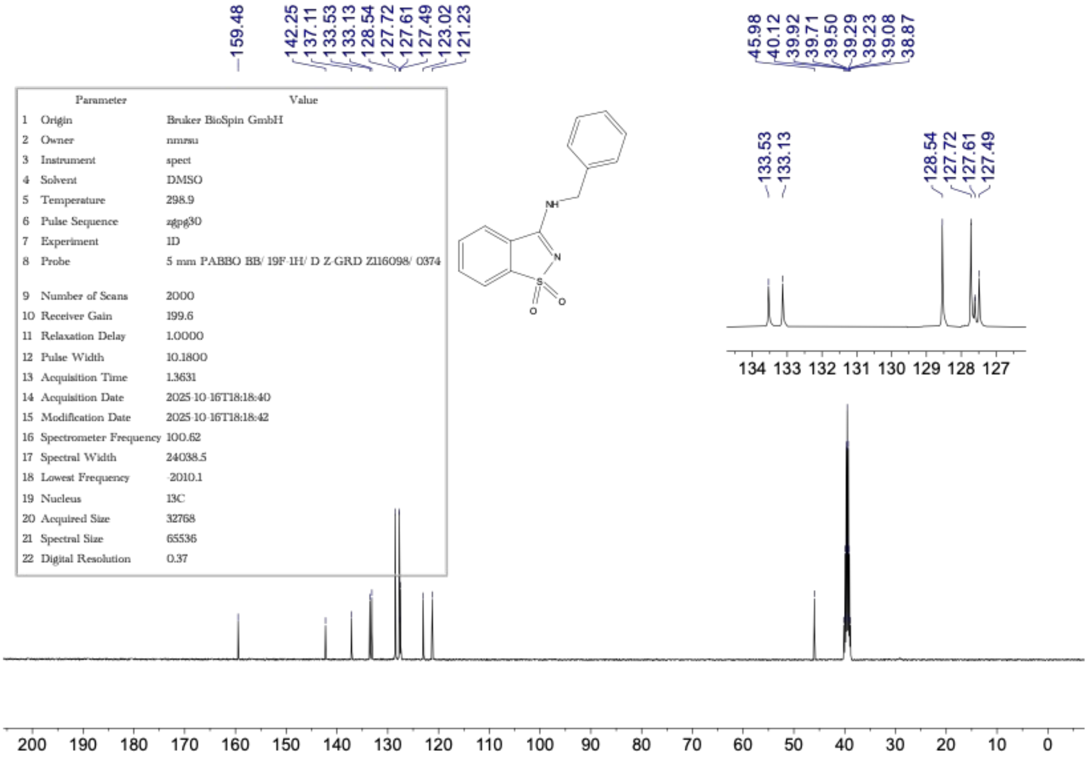

Figure S2. ^{13}C NMR spectrum (100 MHz, DMSO-d_6) of compound **7a**.

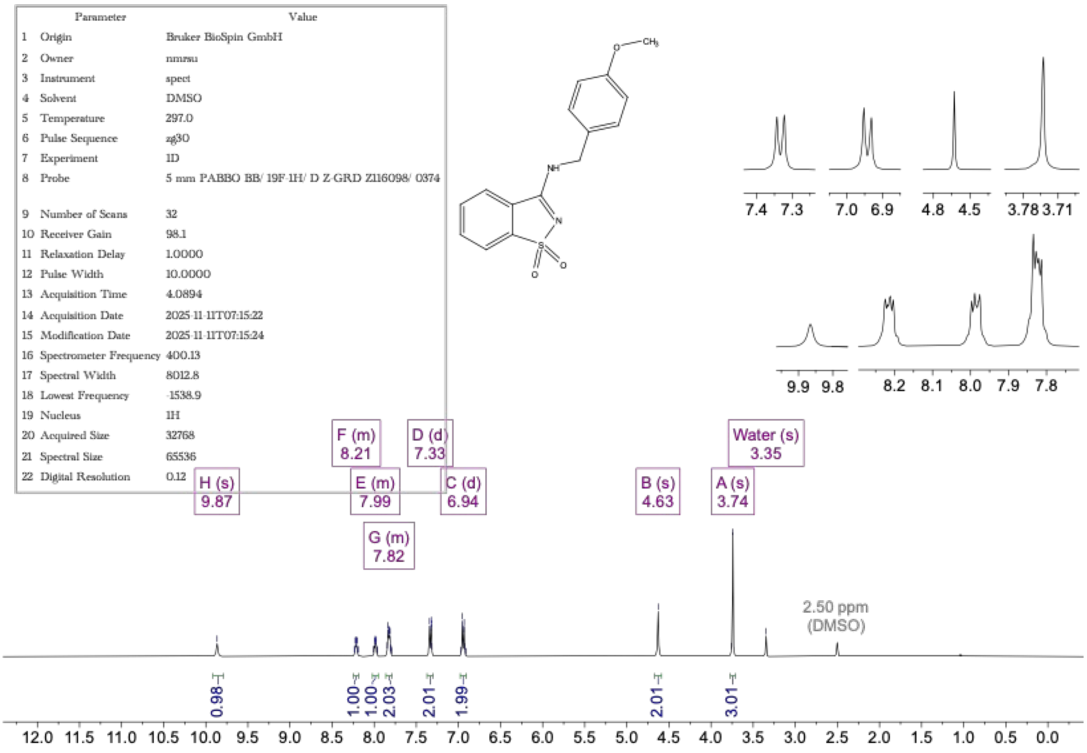

Figure S3. ^1H NMR spectrum (400 MHz, DMSO-d_6) of compound **7b**.

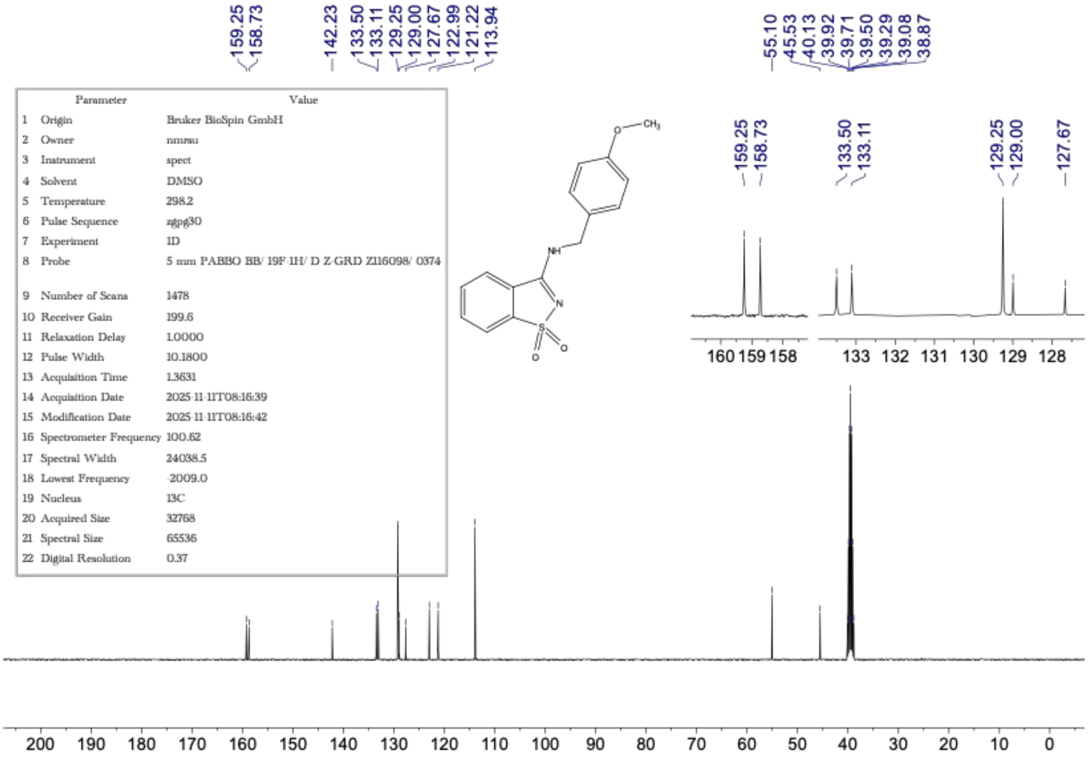

Figure S4. ^{13}C NMR spectrum (100 MHz, DMSO-d_6) of compound **7b**.

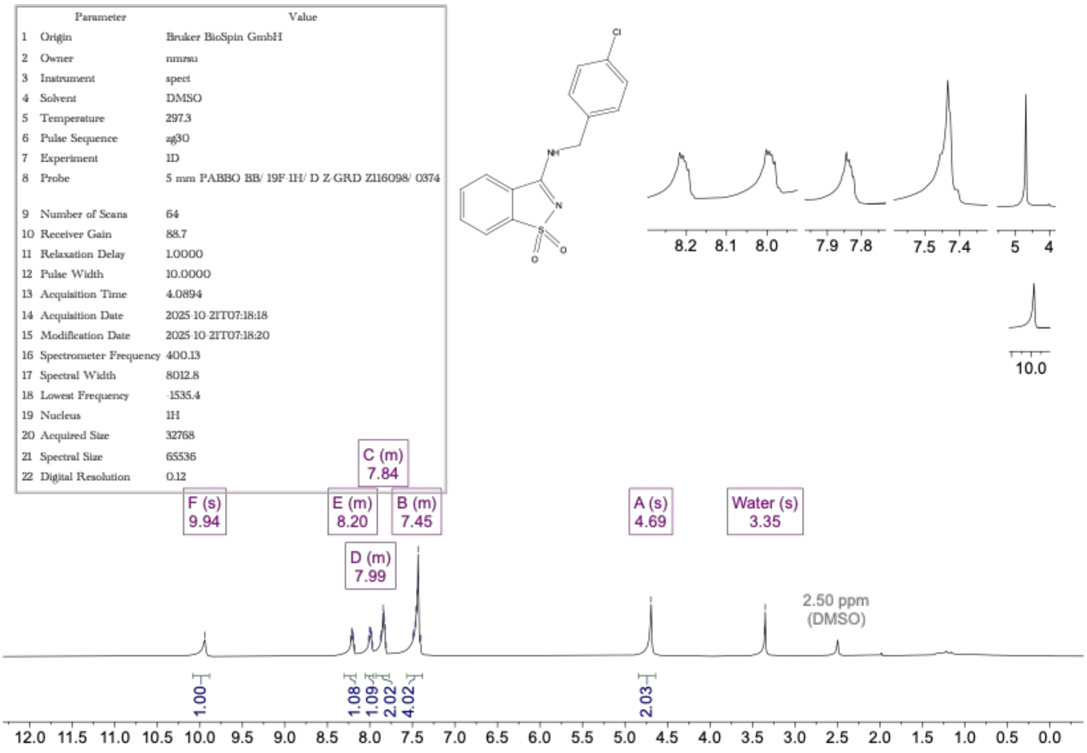

Figure S5. ^{1}H NMR spectrum (400 MHz, DMSO-d_6) of compound **7c**.

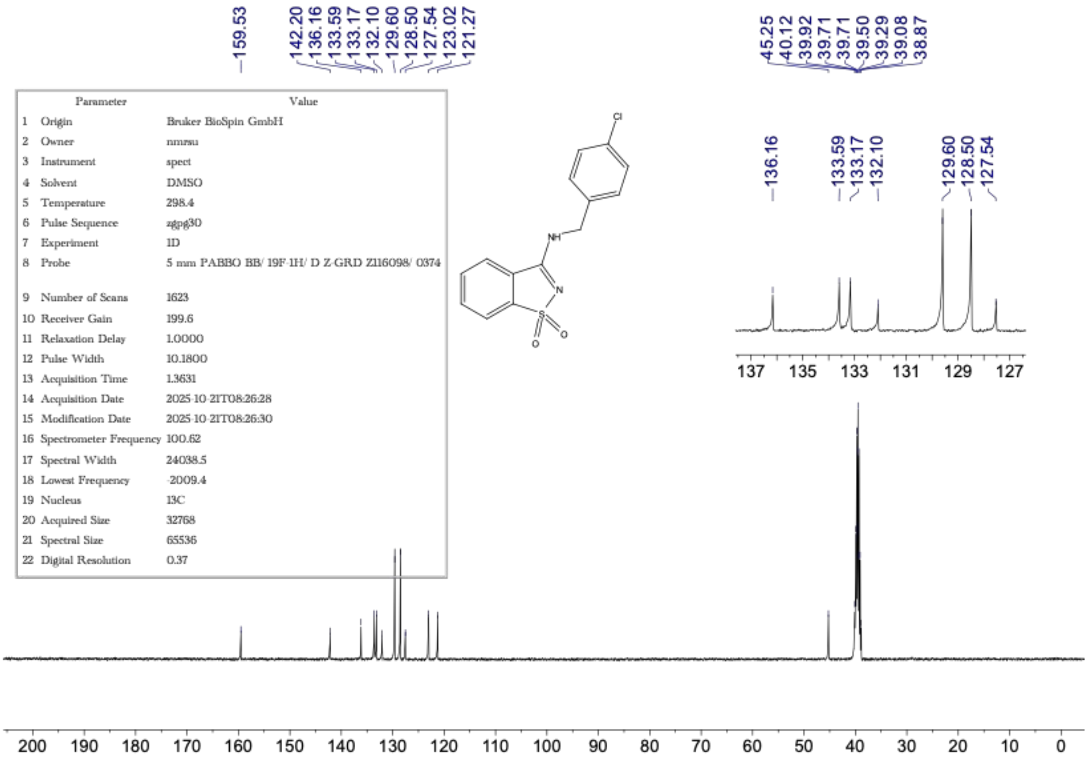

Figure S6. ^{13}C NMR spectrum (100 MHz, DMSO-d_6) of compound **7c**.

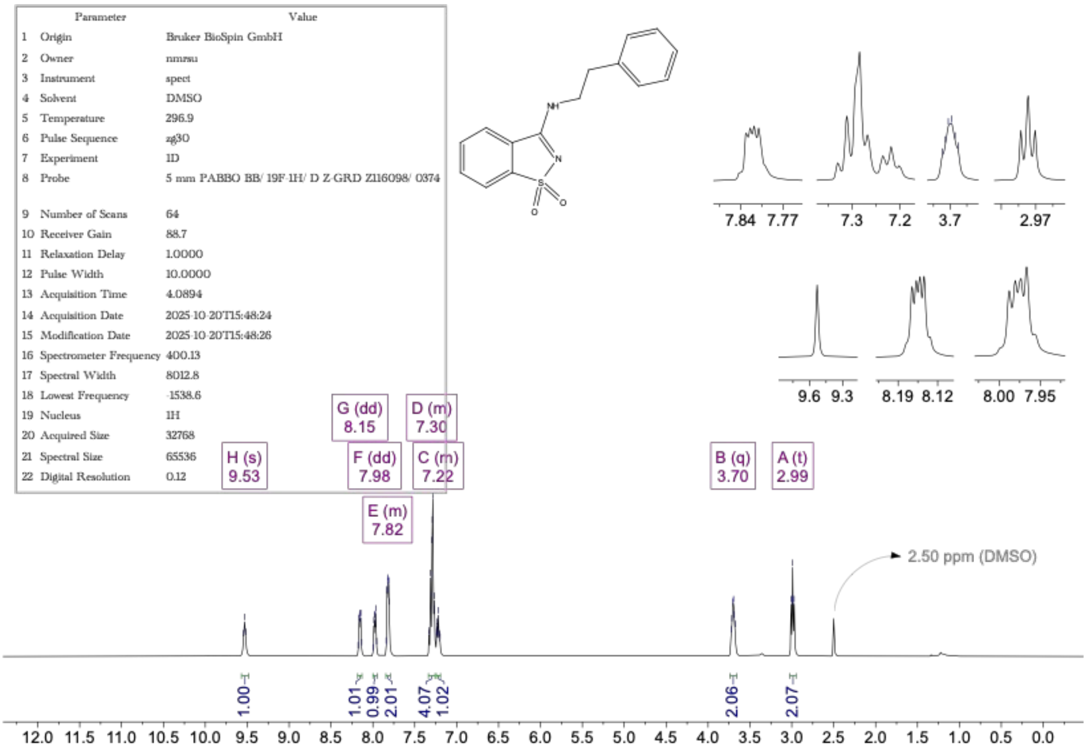

Figure S7. ^{1}H NMR spectrum (400 MHz, DMSO-d_6) of compound **7d**.

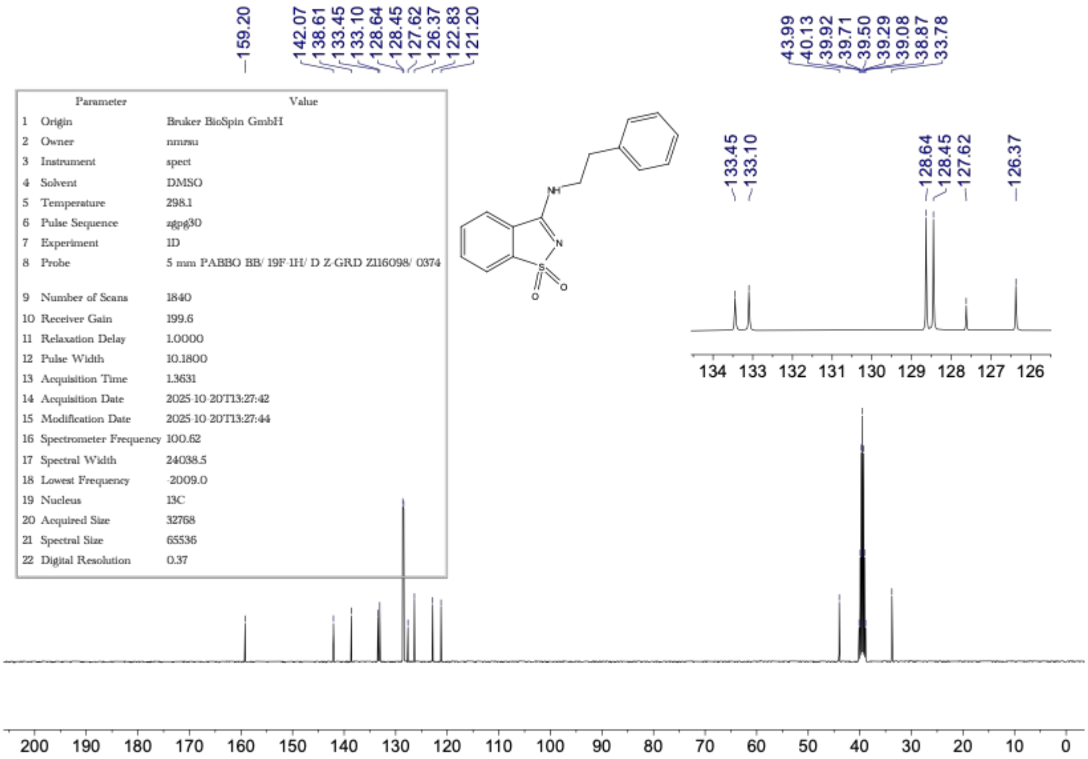

Figure S8. ^{13}C NMR spectrum (100 MHz, DMSO-d_6) of compound **7d**.

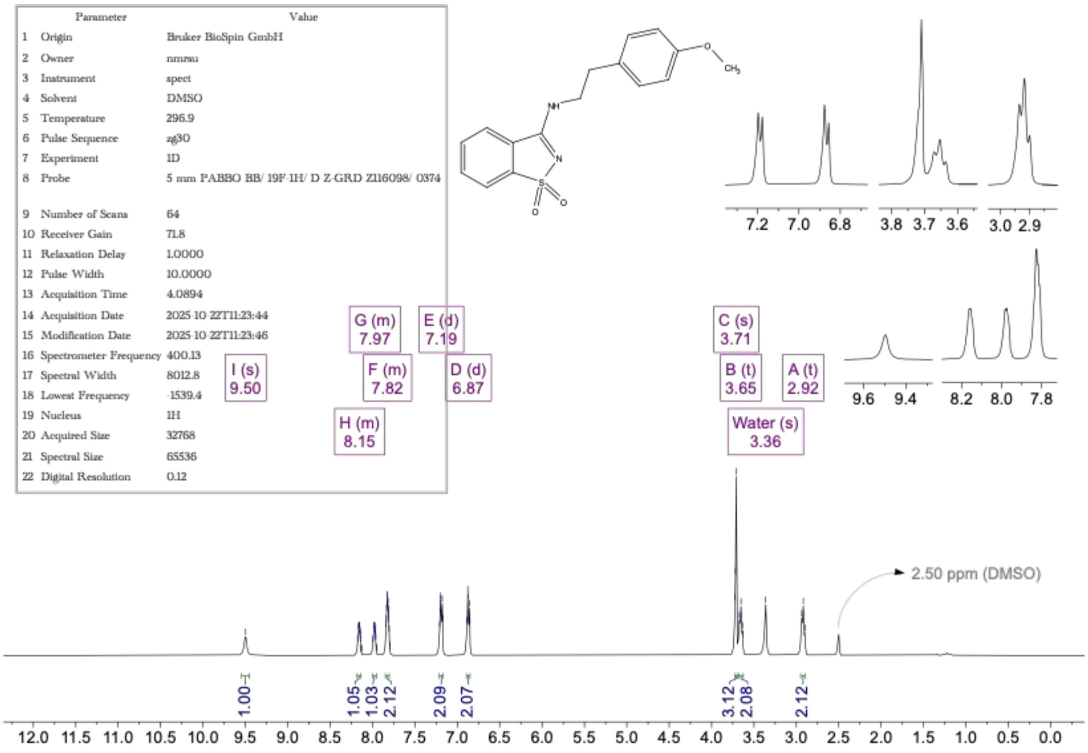

Figure S9. ^{1}H NMR spectrum (400 MHz, DMSO-d_6) of compound **7e**.

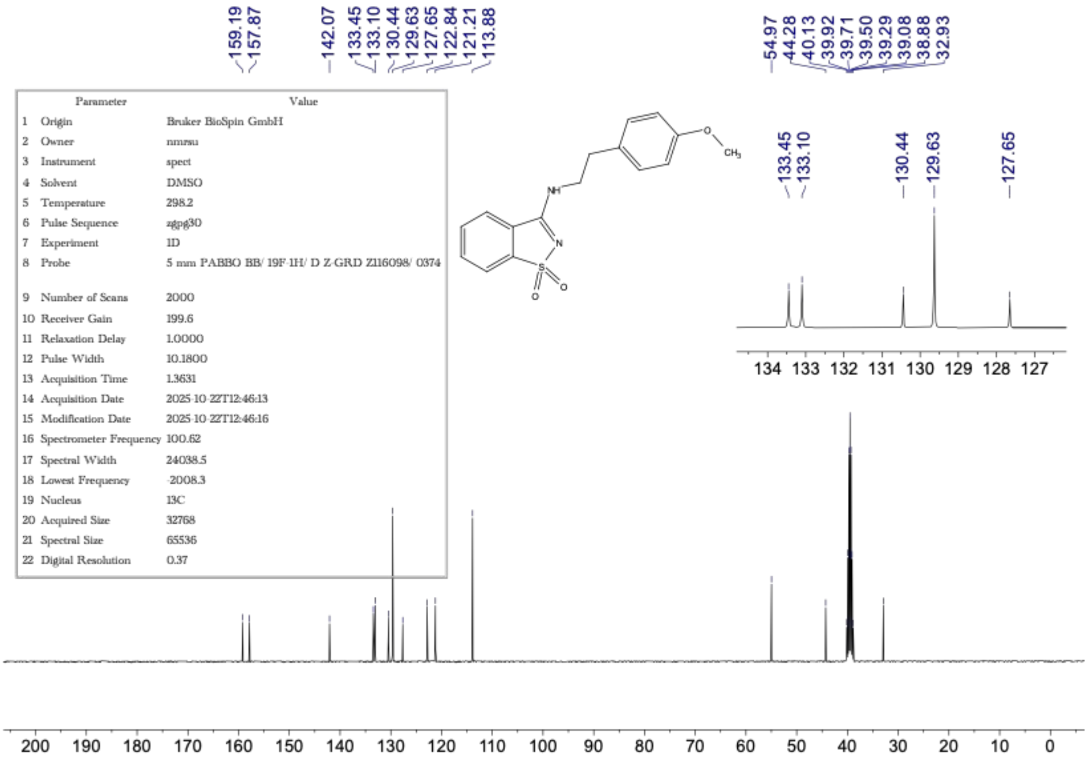

Figure S10. ^{13}C NMR spectrum (100 MHz, DMSO-d_6) of compound **7e**.

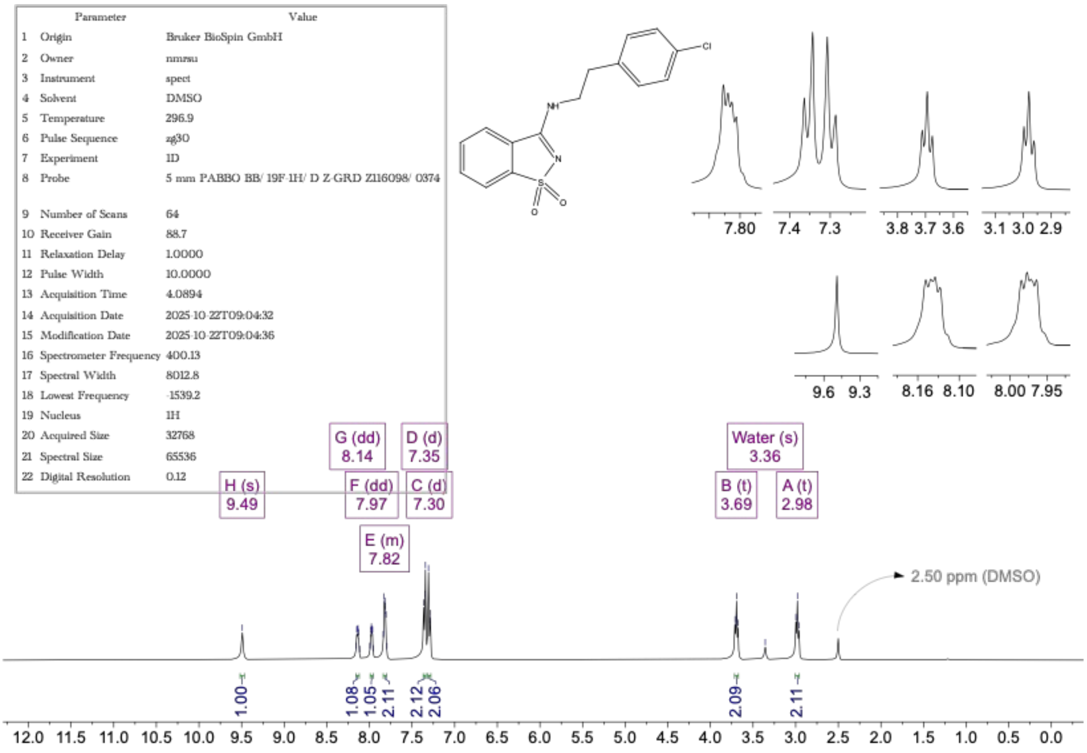

Figure S11. ^{1}H NMR spectrum (400 MHz, DMSO-d_6) of compound **7f**.

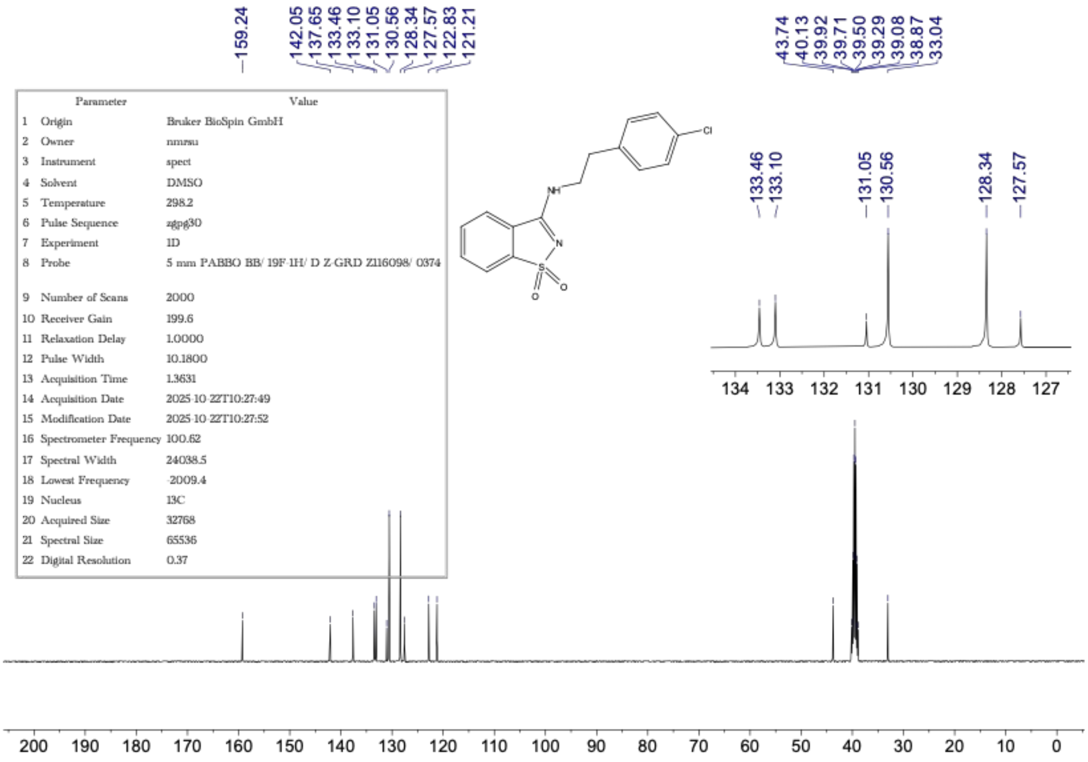

Figure S12. ^{13}C NMR spectrum (100 MHz, DMSO-d_6) of compound **7f**.

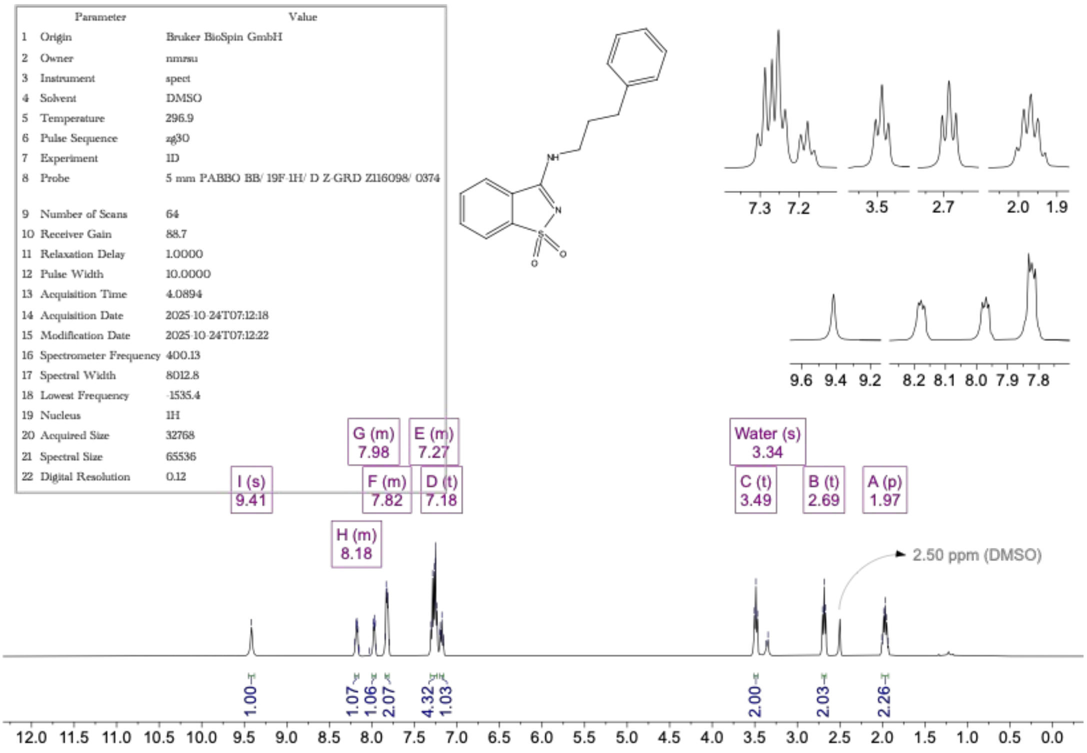

Figure S13. ^{1}H NMR spectrum (400 MHz, DMSO-d_6) of compound **7g**.

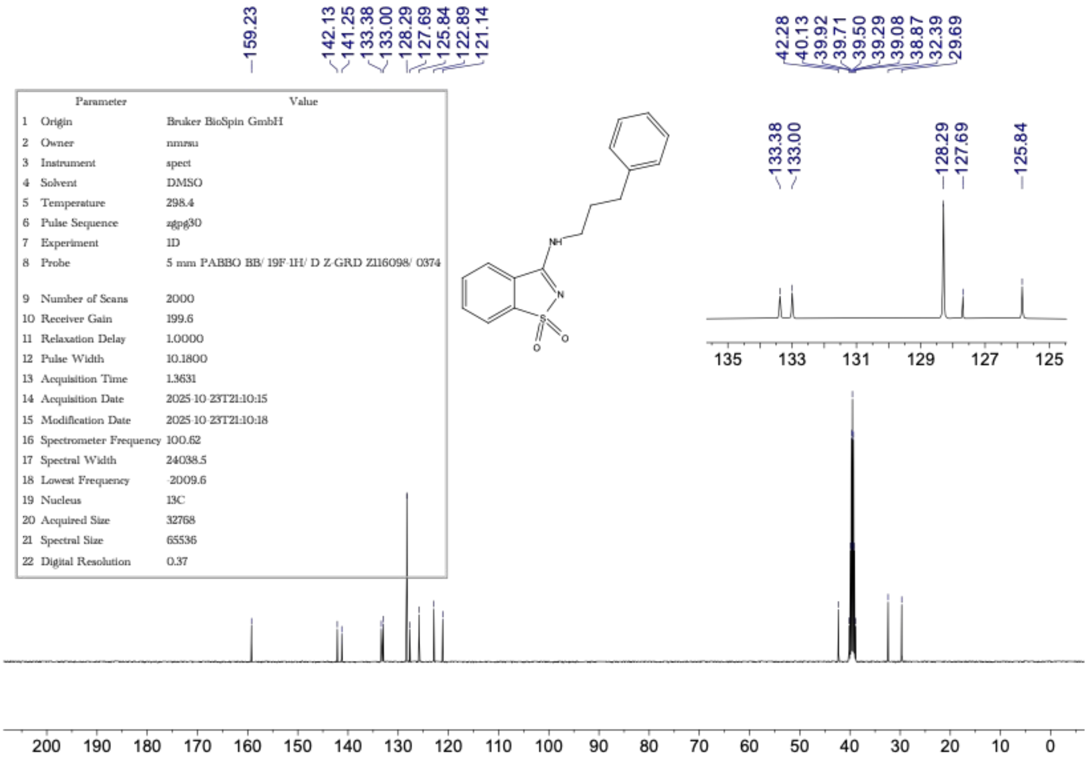

Figure S14. ^{13}C NMR spectrum (100 MHz, DMSO-d_6) of compound **7g**.

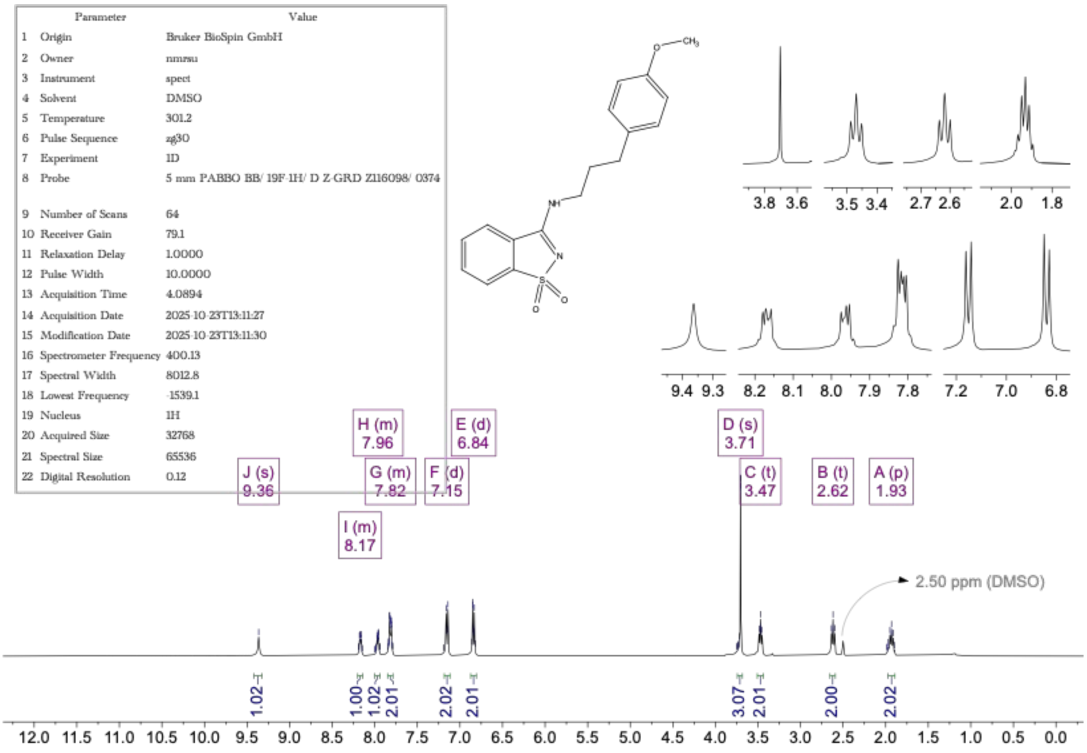

Figure S15. ^{1}H NMR spectrum (400 MHz, DMSO-d_6) of compound **7h**.

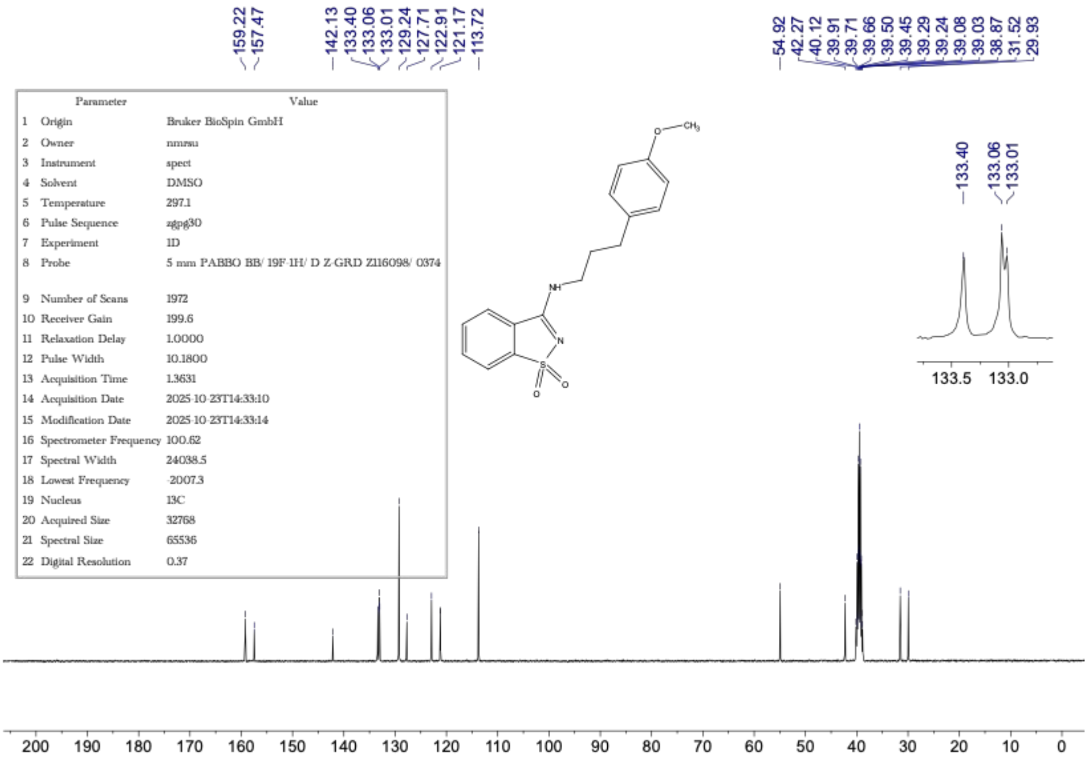

Figure S16. ^{13}C NMR spectrum (100 MHz, DMSO-d_6) of compound **7h**.

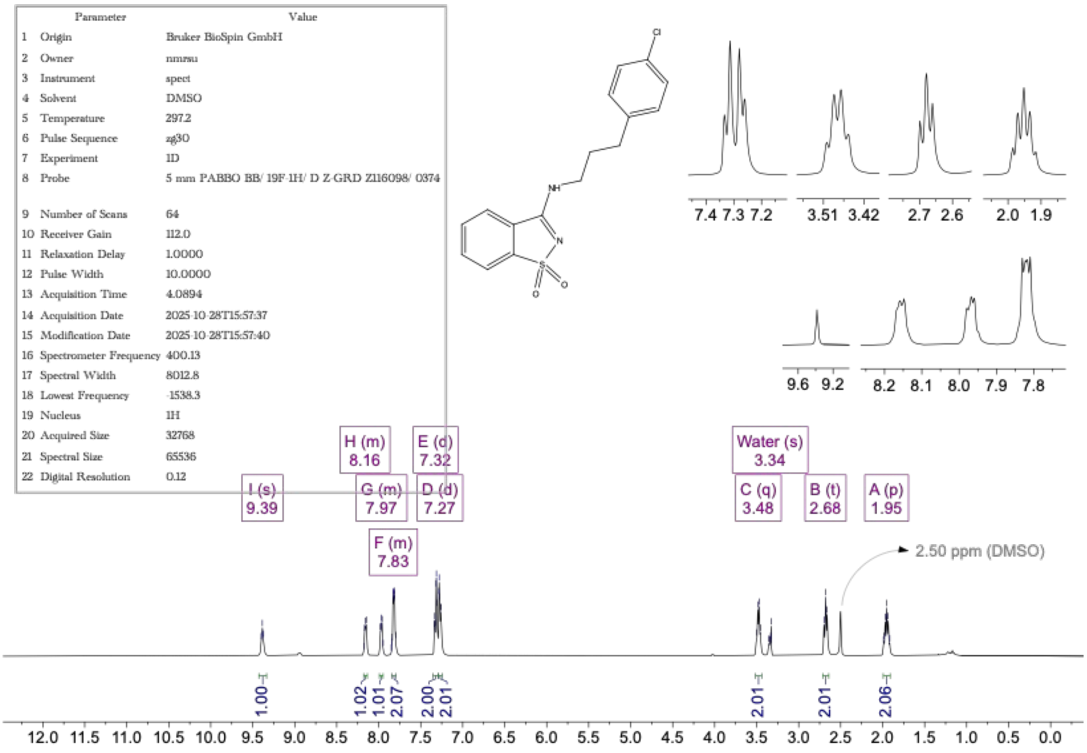

Figure S17. ^{1}H NMR spectrum (400 MHz, DMSO-d_6) of compound **7i**.

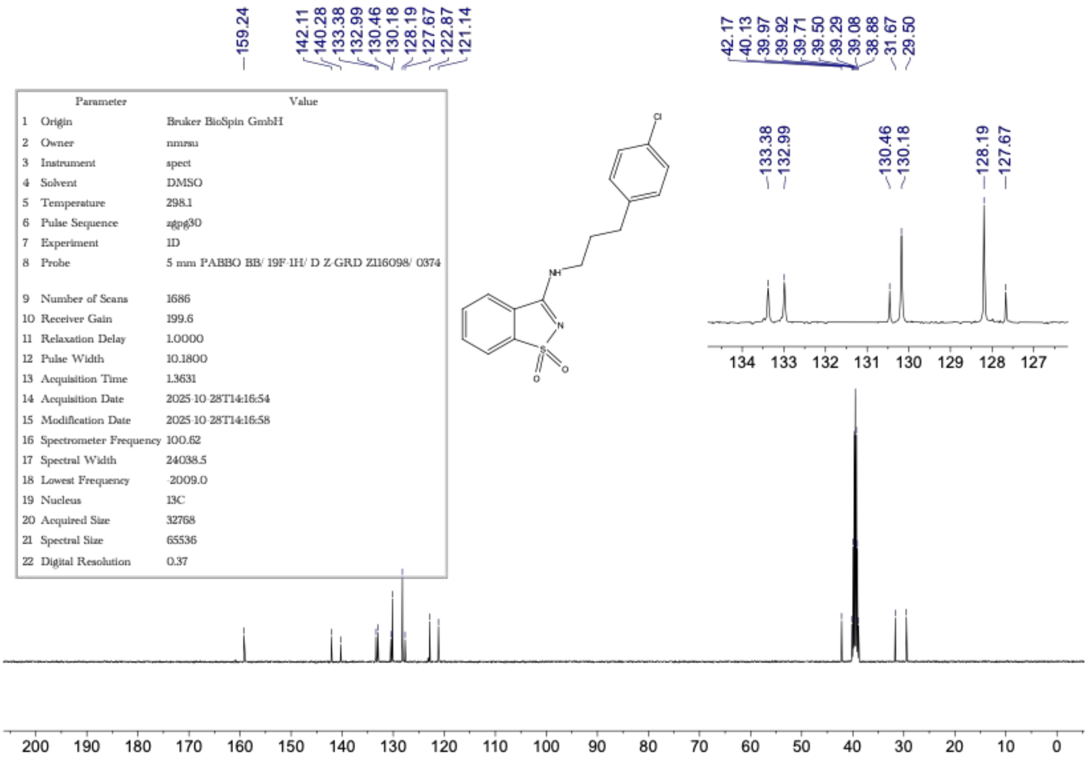

Figure S18. ^{13}C NMR spectrum (100 MHz, DMSO-d_6) of compound **7i**.

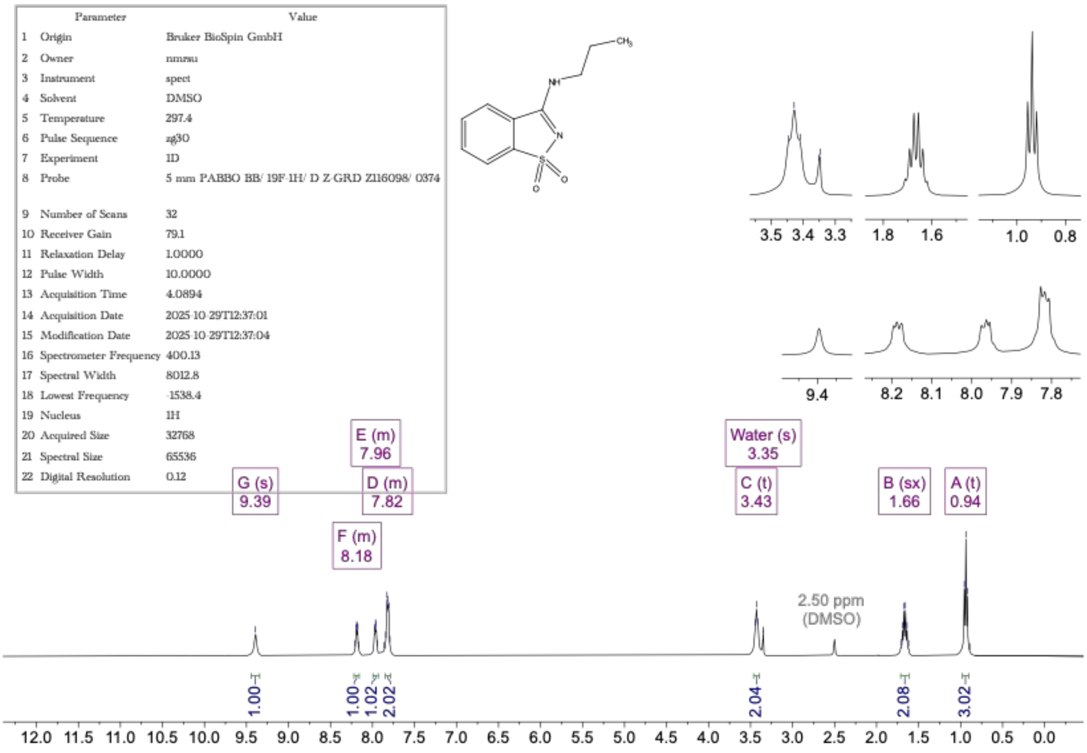

Figure S19. ^{1}H NMR spectrum (400 MHz, DMSO-d_6) of compound **7j**.

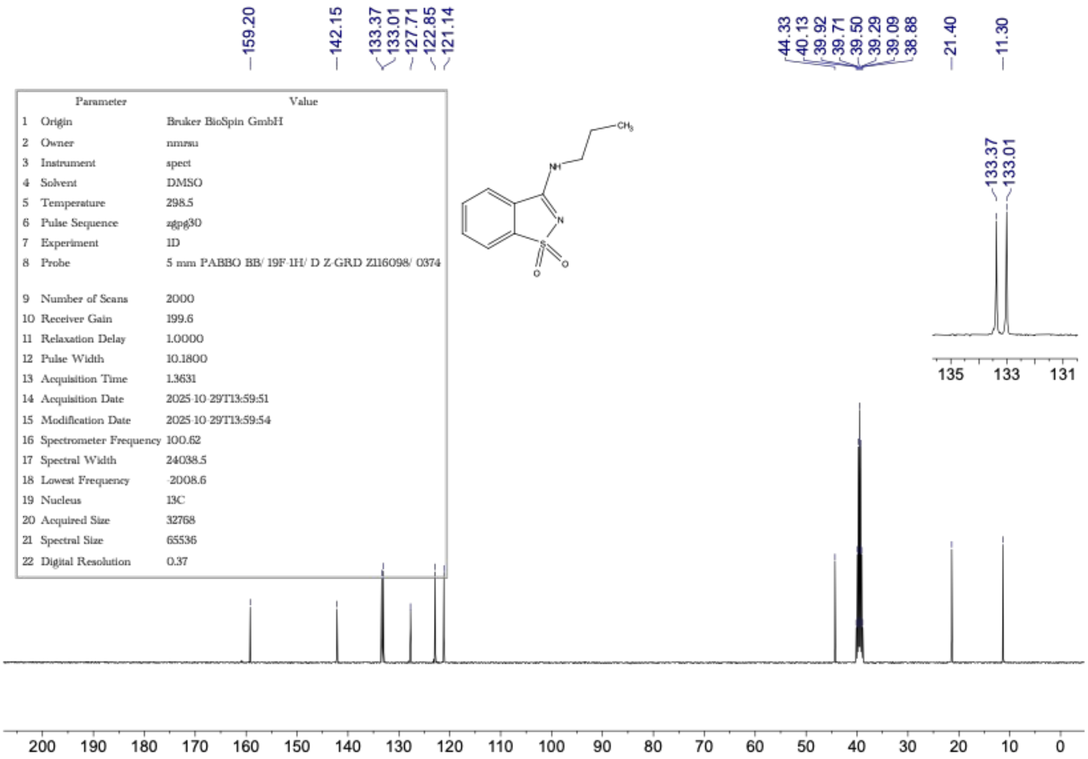

Figure S20. ^{13}C NMR spectrum (100 MHz, DMSO-d_6) of compound **7j**.

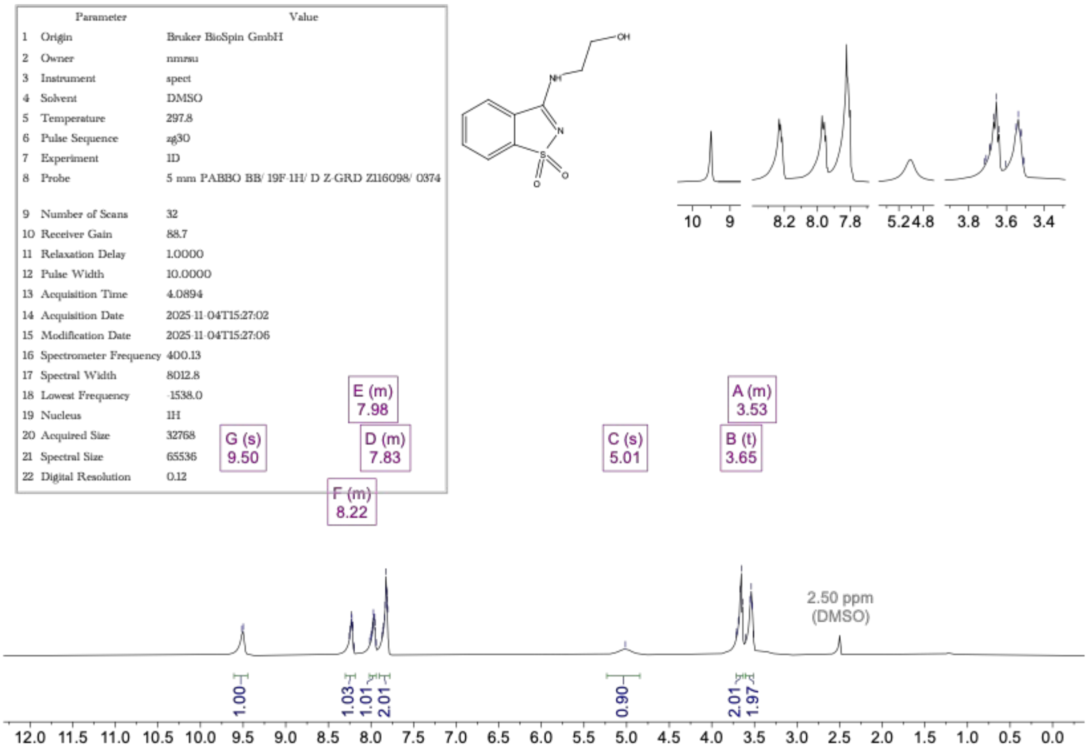

Figure S21. ^{1}H NMR spectrum (400 MHz, DMSO-d_6) of compound **7k**.

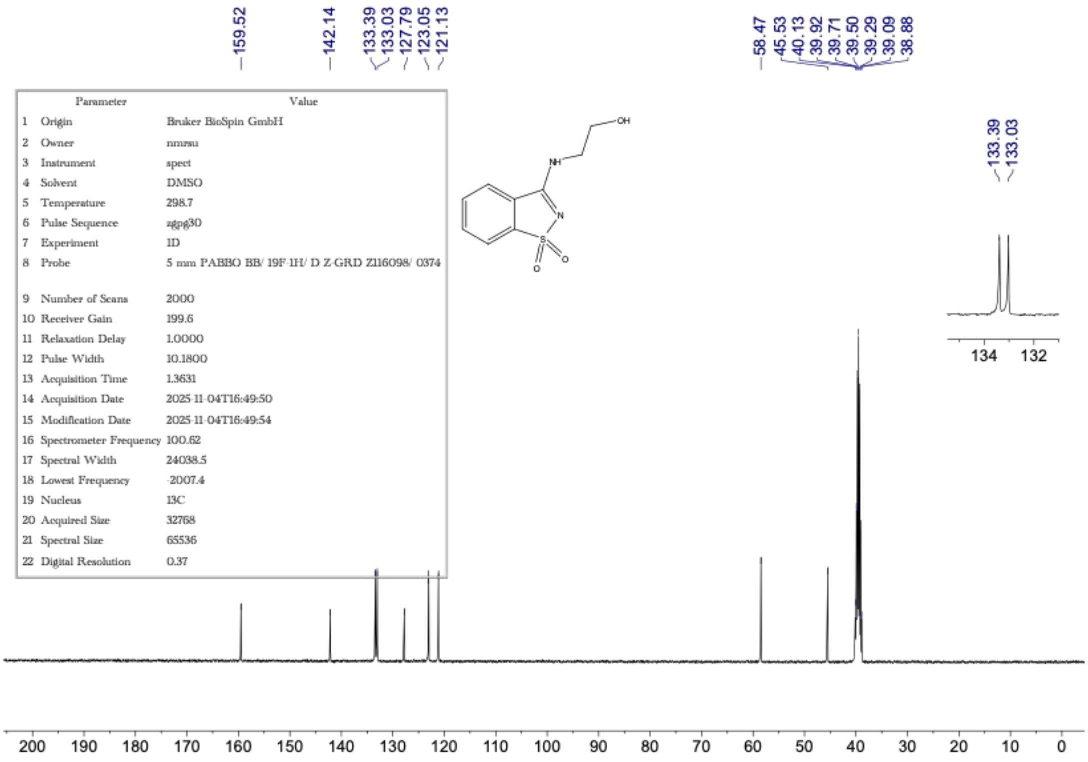

Figure S22. ^{13}C NMR spectrum (100 MHz, DMSO-d_6) of compound **7k**.

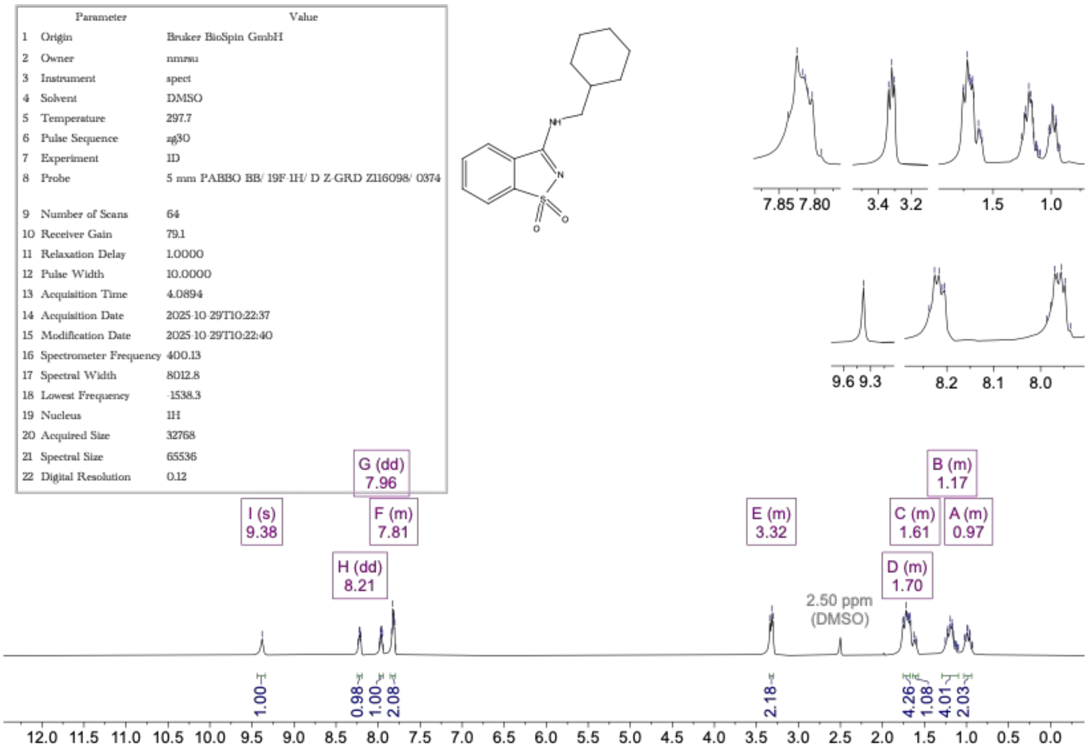

Figure S23. ^{1}H NMR spectrum (400 MHz, DMSO-d_6) of compound **7l**.

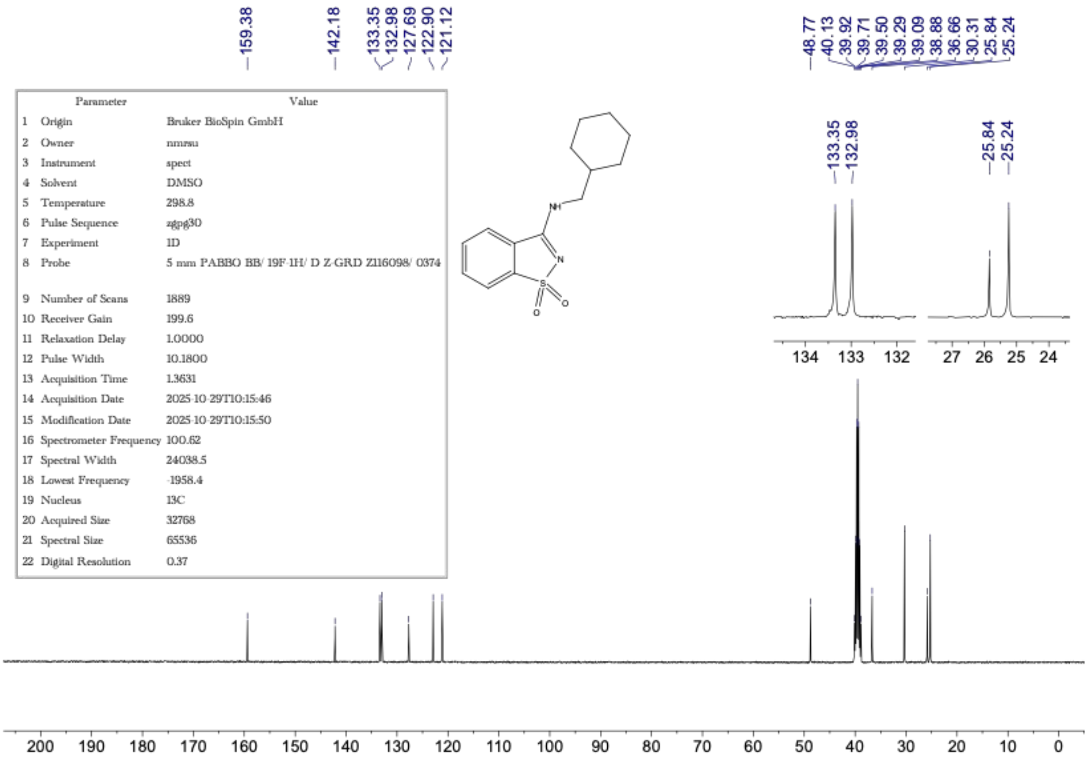

Figure S24. ^{13}C NMR spectrum (100 MHz, DMSO-d_6) of compound **7l**.

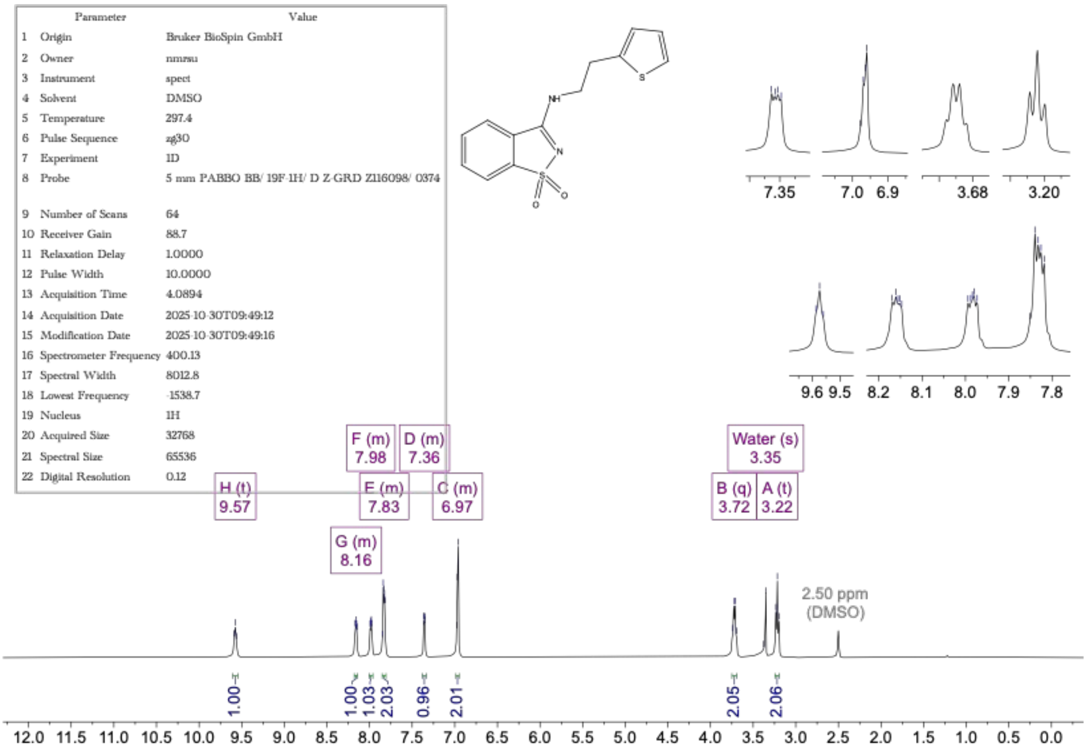

Figure S25. ^{1}H NMR spectrum (400 MHz, DMSO-d_6) of compound **7m**.

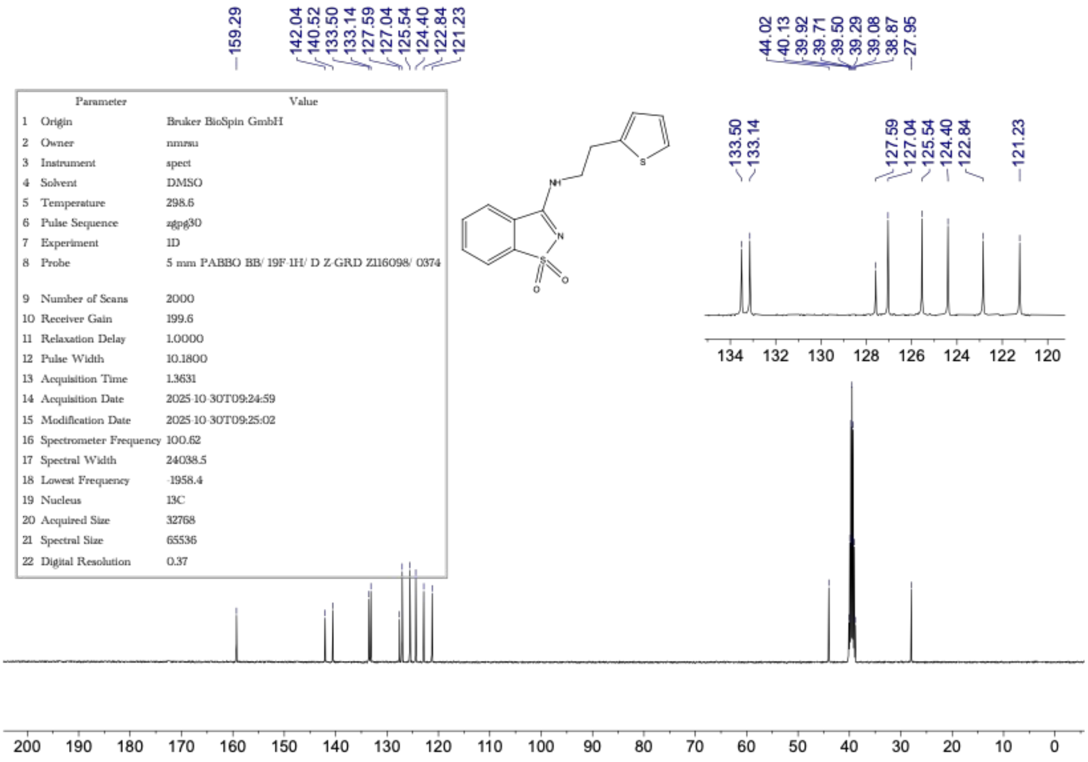

Figure S26. ^{13}C NMR spectrum (100 MHz, DMSO-d_6) of compound **7m**.

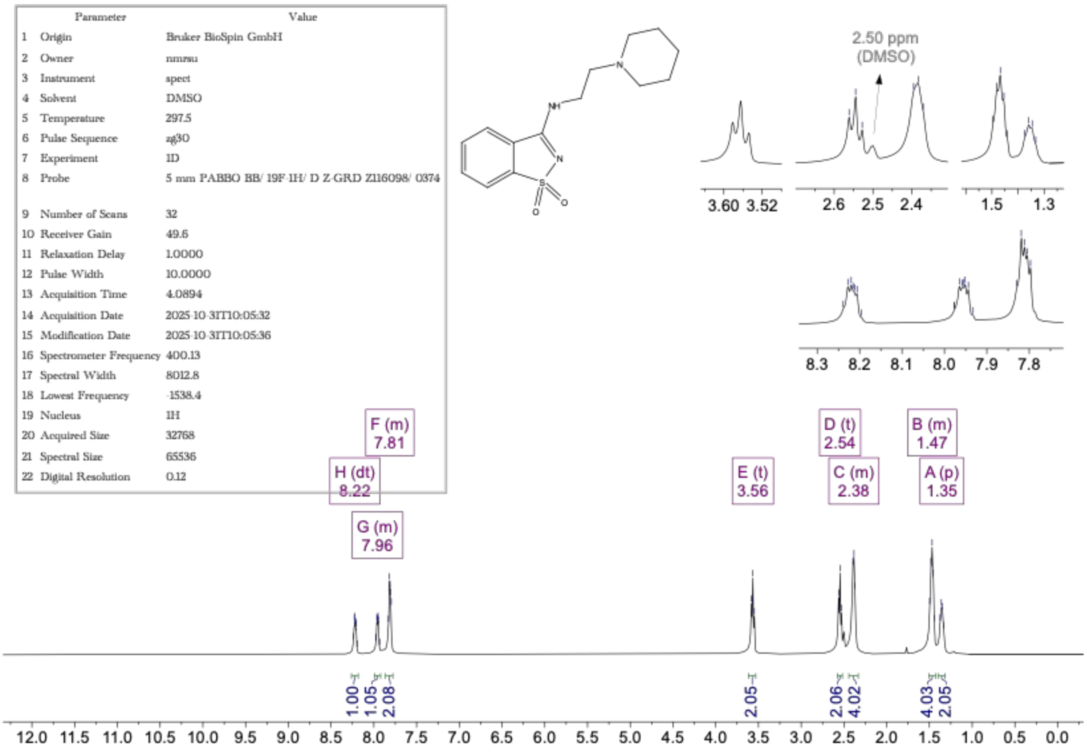

Figure S27. ^{1}H NMR spectrum (400 MHz, DMSO-d_6) of compound **7n**.

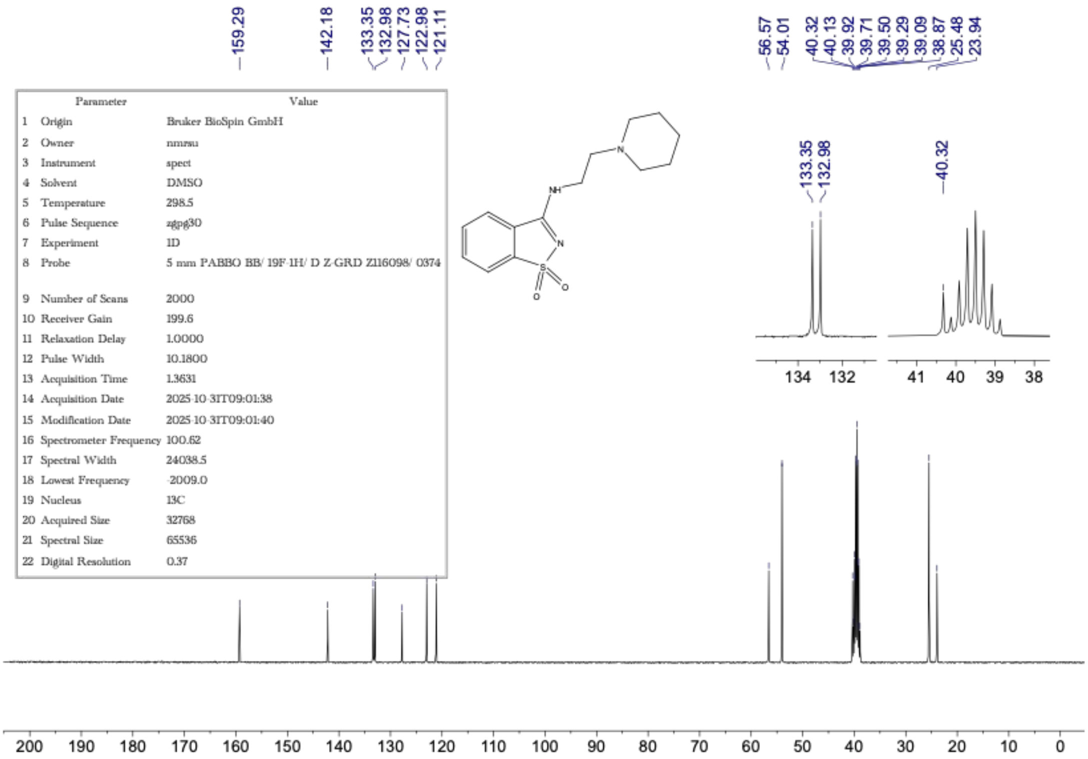

Figure S28. ^{13}C NMR spectrum (100 MHz, DMSO-d_6) of compound **7n**.

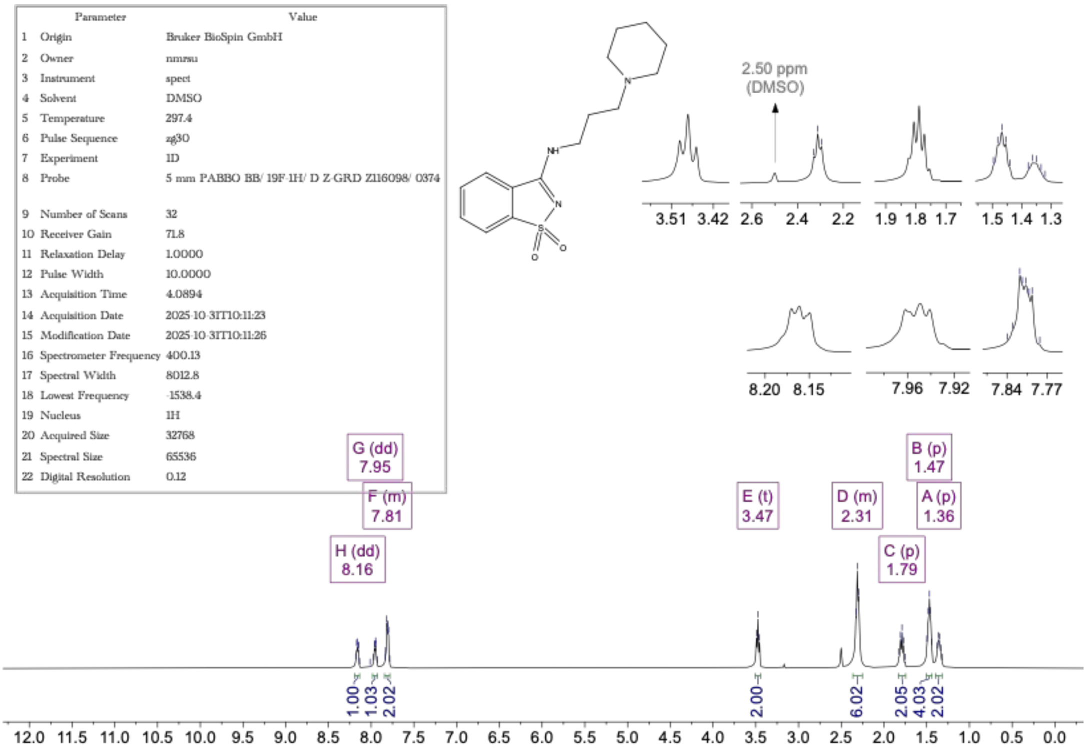

Figure S29. ^{1}H NMR spectrum (400 MHz, DMSO-d_6) of compound **7o**.

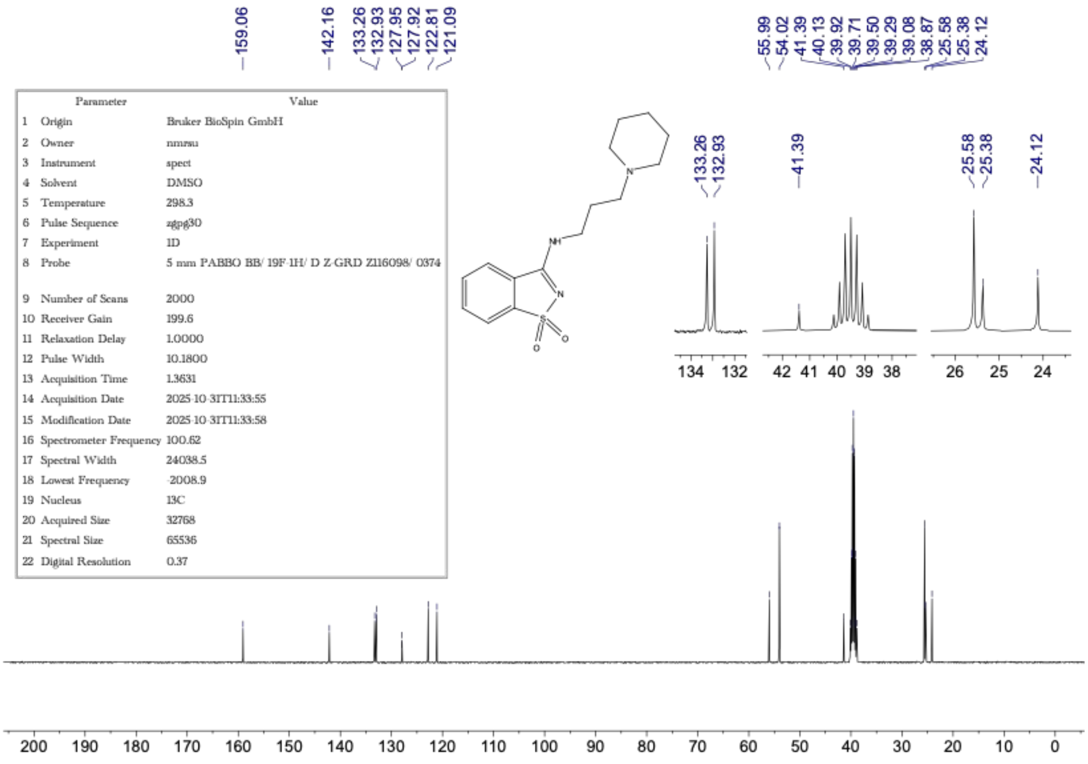

Figure S30. ^{13}C NMR spectrum (100 MHz, DMSO-d_6) of compound **7o**.

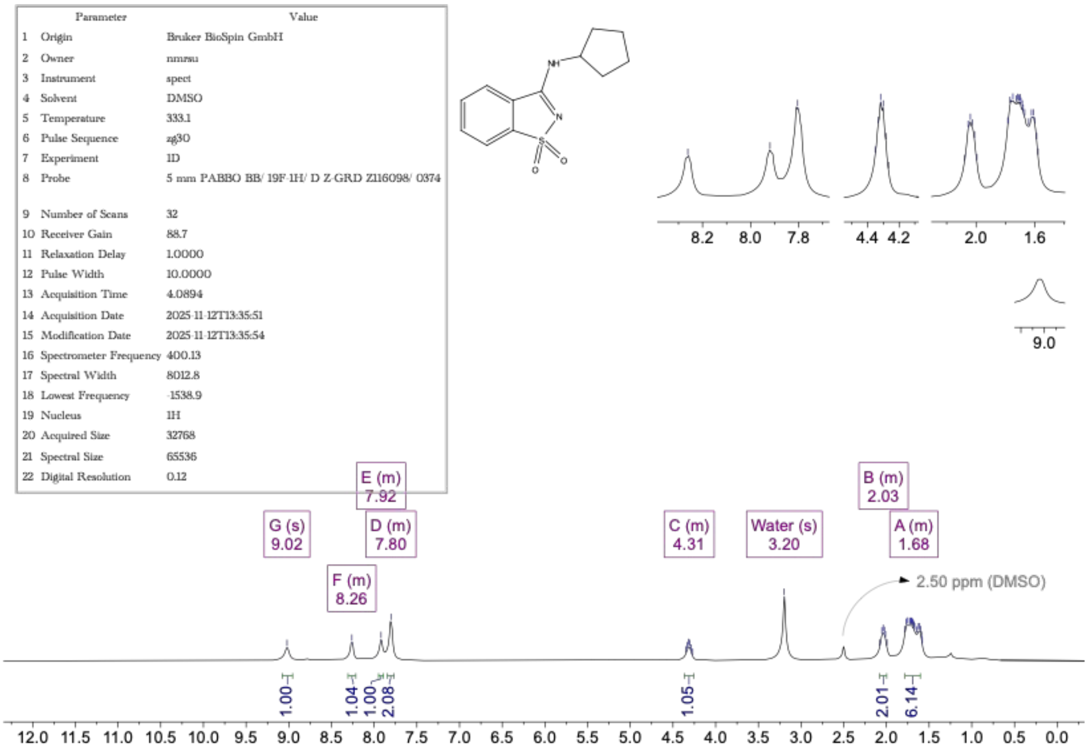

Figure S31. ^{1}H NMR spectrum (400 MHz, DMSO-d_6) of compound **7p**.

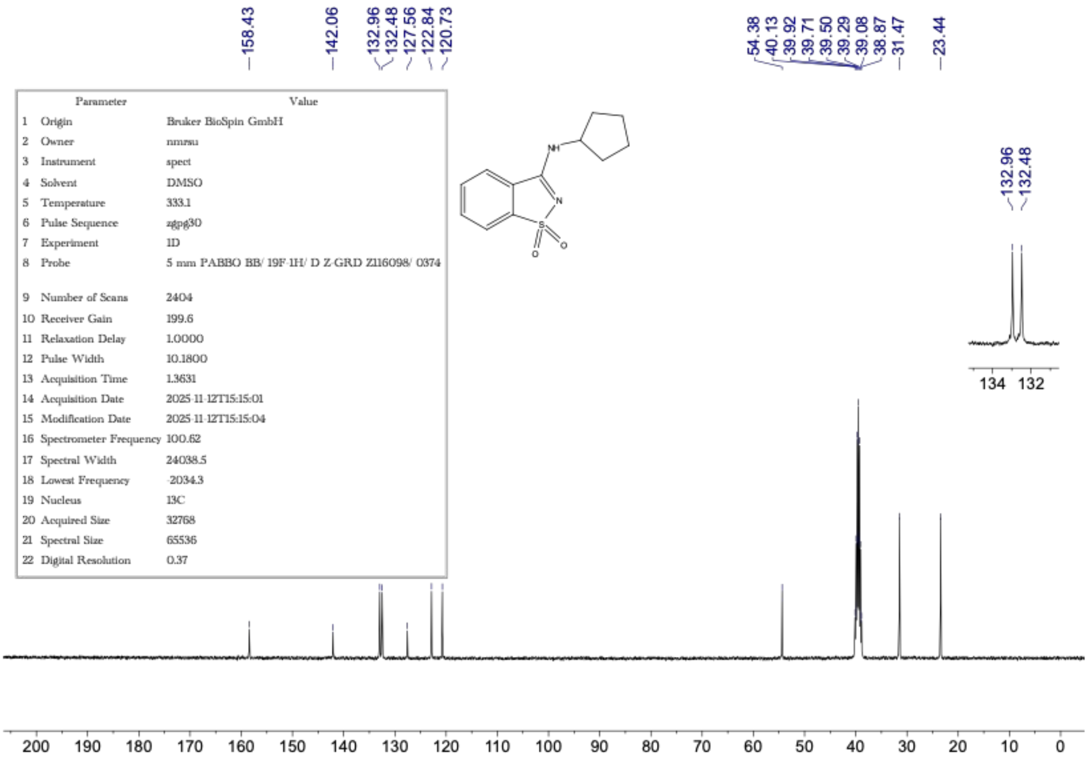

Figure S32. ^{13}C NMR spectrum (100 MHz, DMSO-d_6) of compound **7p**.

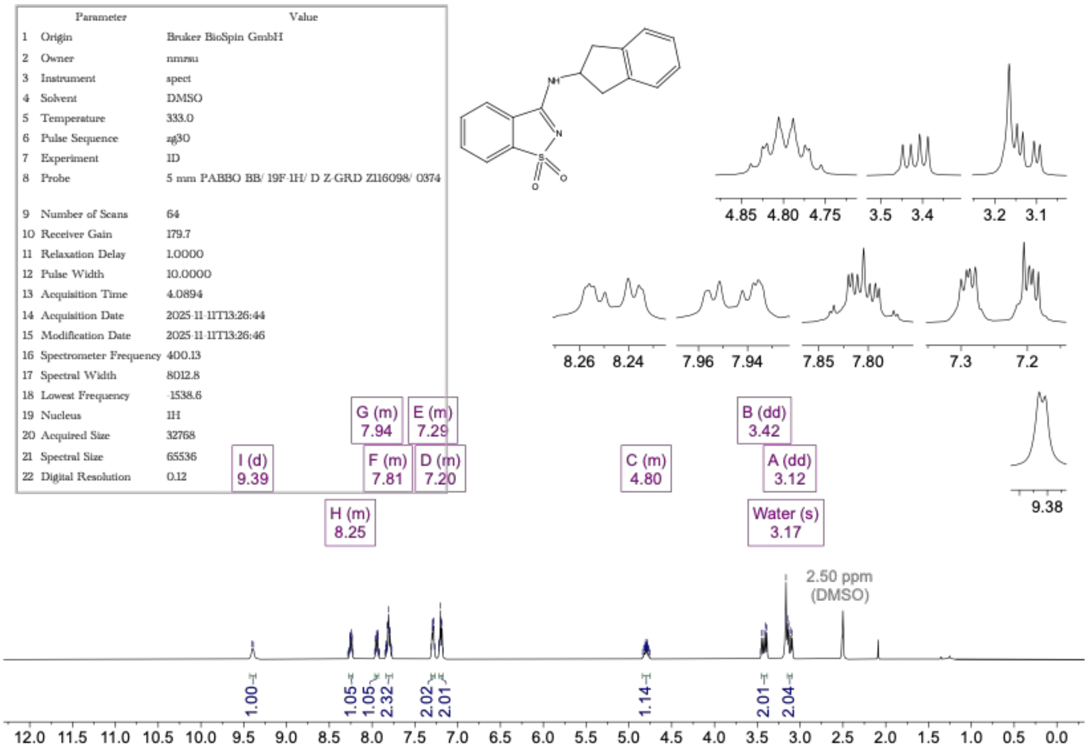

Figure S33. ^1H NMR spectrum (400 MHz, DMSO-d_6) of compound **7q**.

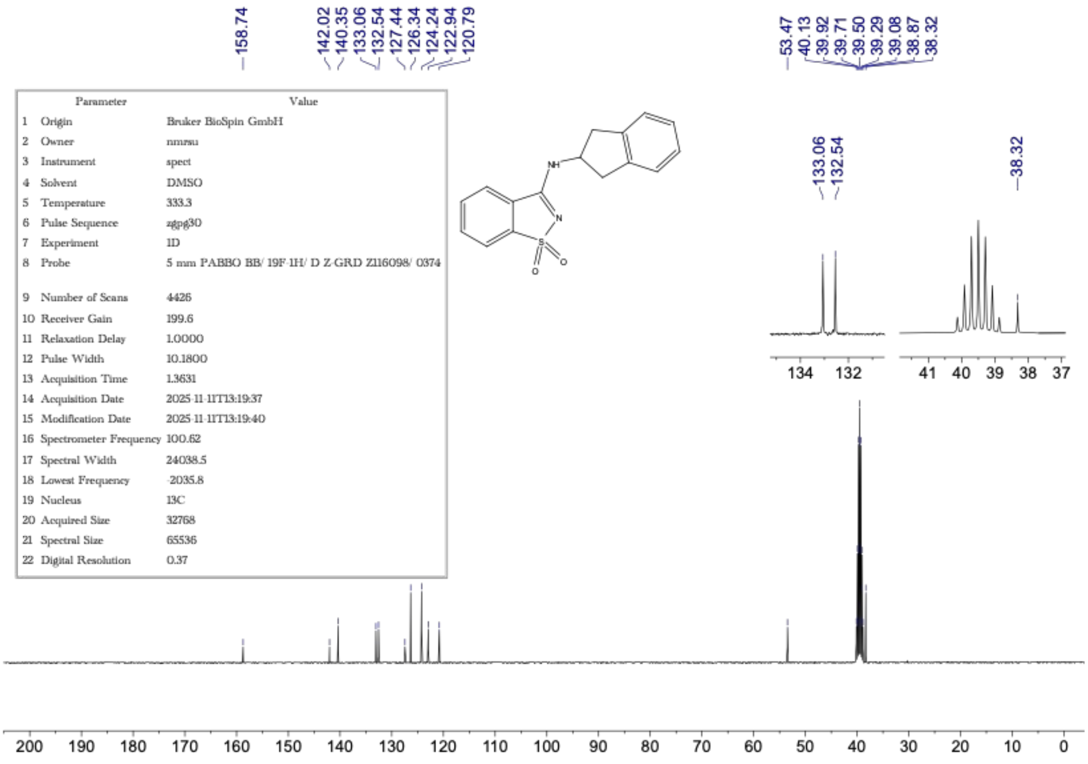

Figure S34. ^{13}C NMR spectrum (100 MHz, DMSO-d_6) of compound **7q**.

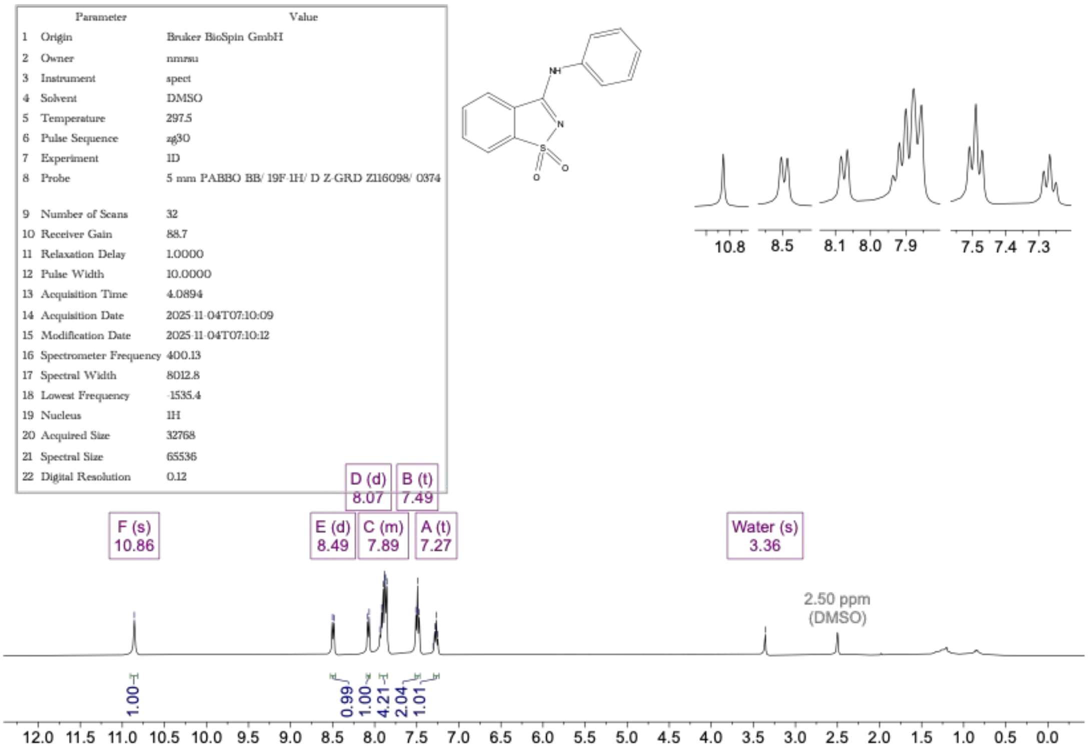

Figure S35. ^{1}H NMR spectrum (400 MHz, DMSO-d_6) of compound **7r**.

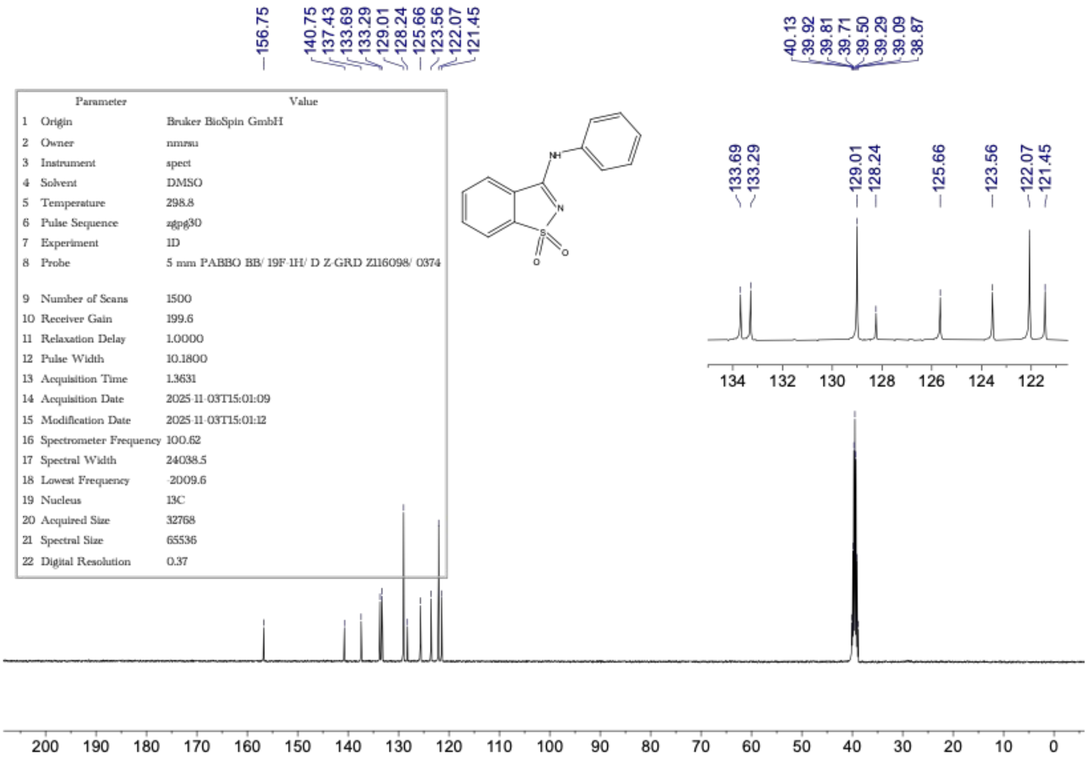

Figure S36. ^{13}C NMR spectrum (100 MHz, DMSO-d_6) of compound **7r**.

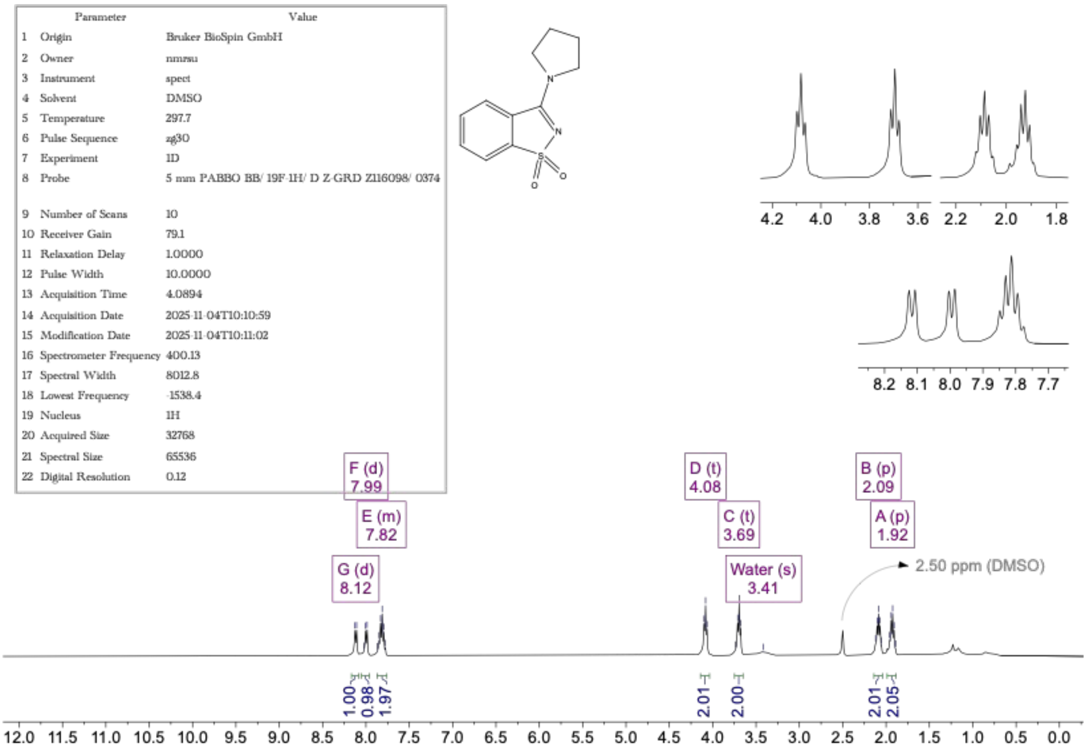

Figure S37. ^{1}H NMR spectrum (400 MHz, DMSO-d_6) of compound **7s**.

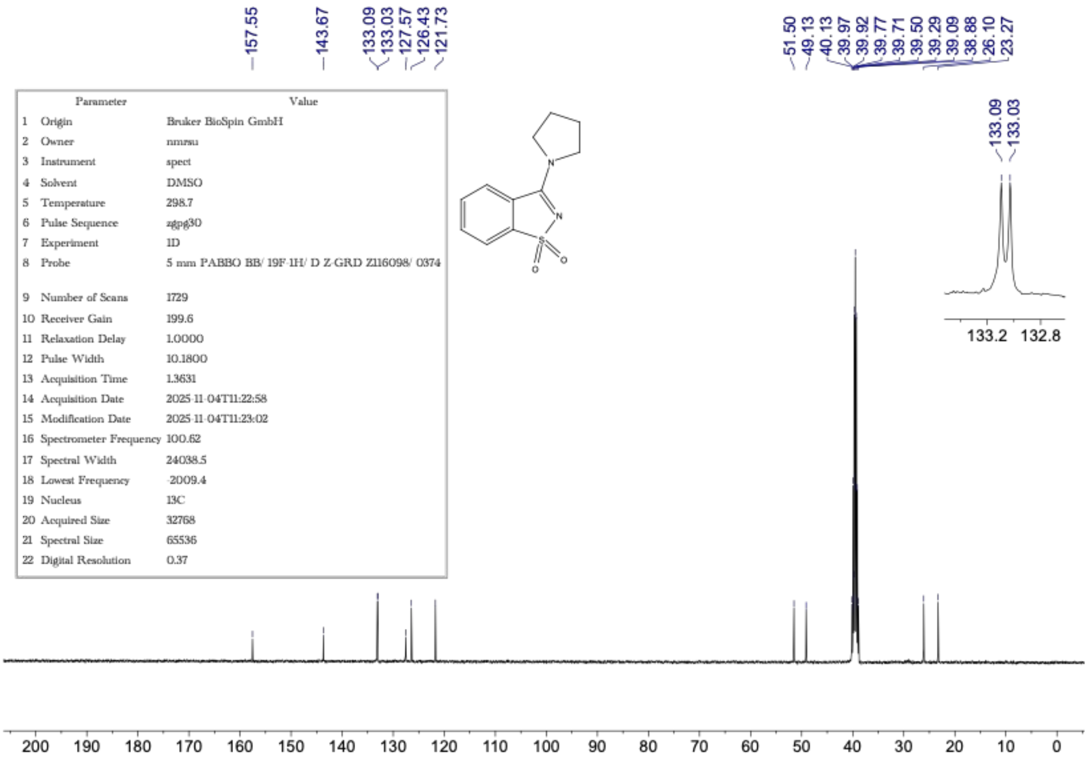

Figure S38. ^{13}C NMR spectrum (100 MHz, DMSO-d_6) of compound **7s**.

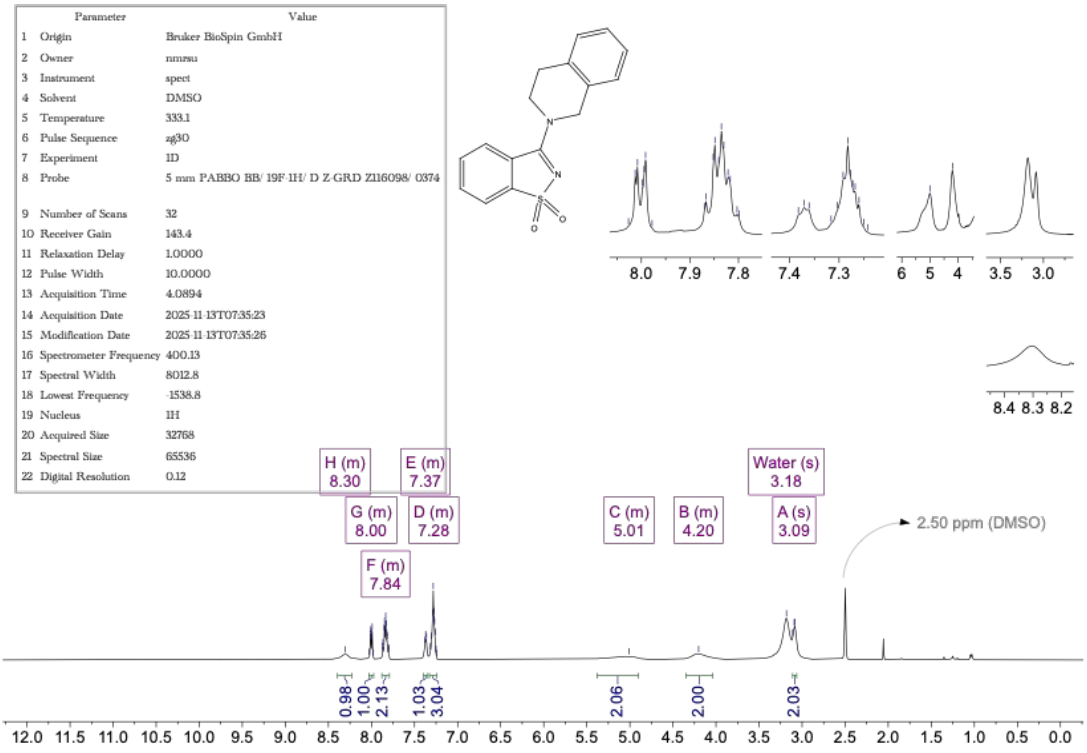

Figure S39. ^{1}H NMR spectrum (400 MHz, DMSO-d_6) of compound **7t**.

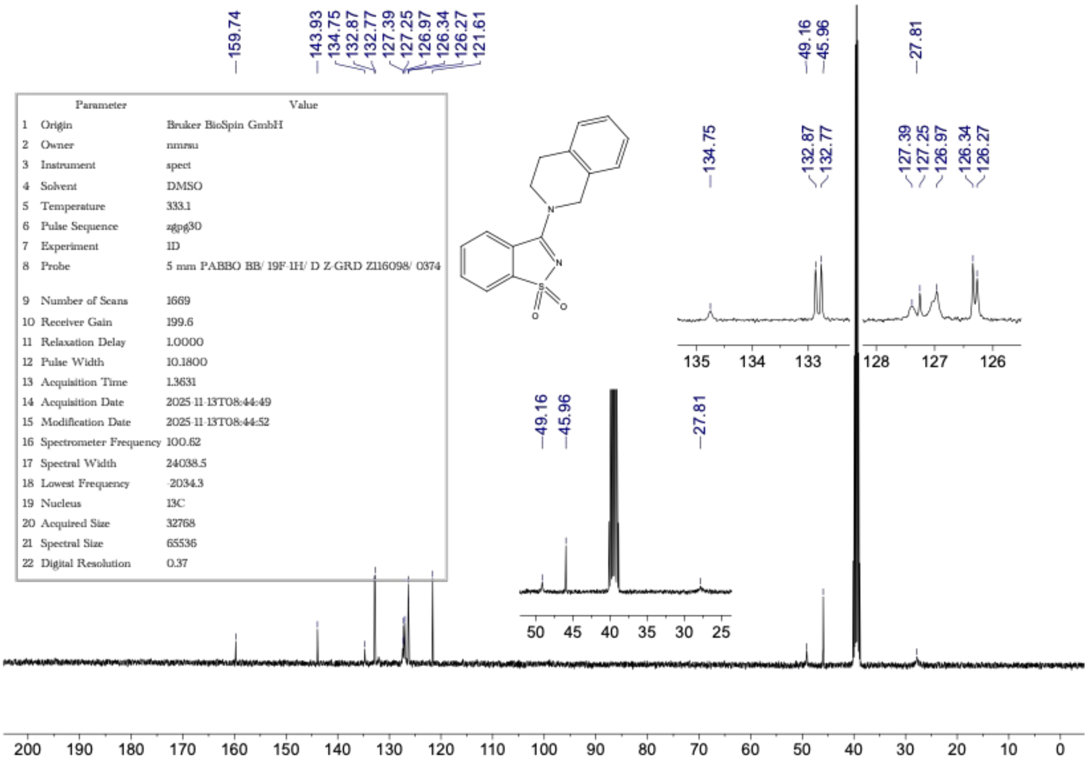

Figure S40. ^{13}C NMR spectrum (100 MHz, DMSO-d_6) of compound **7t**.

3.2. Spectroscopic and mass spectrometric data of the putative tetrahedral adduct (7c-adduct)

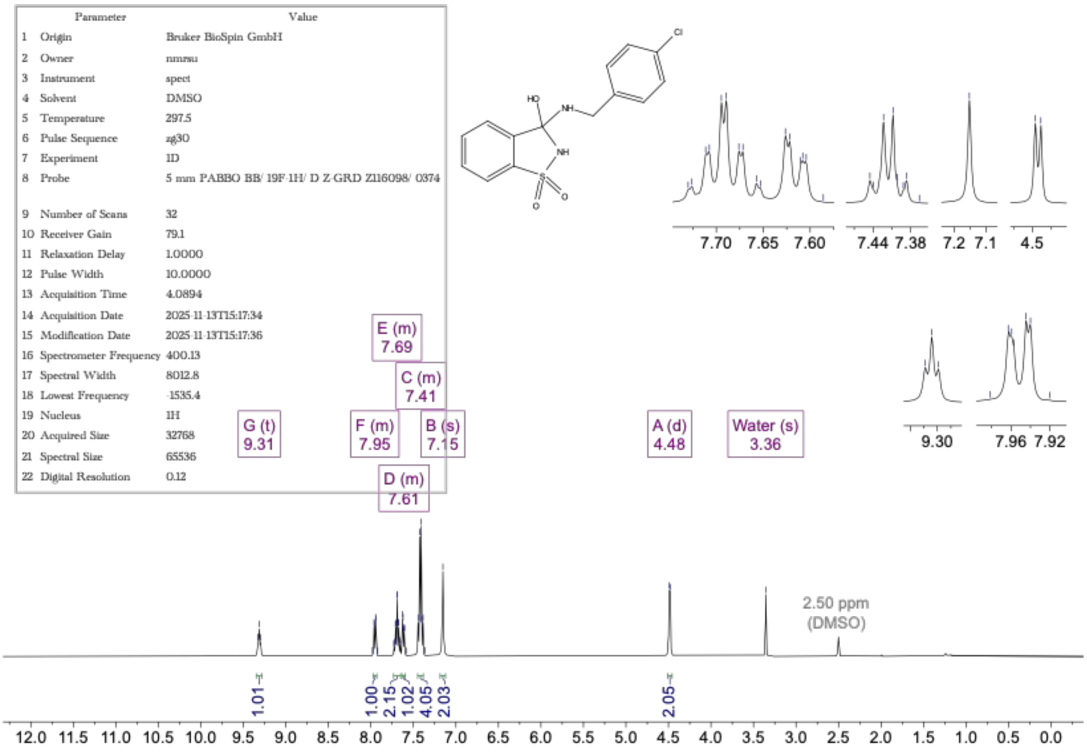

Figure S41. ^{1}H NMR spectrum (400 MHz, DMSO-d$_6$) of compound **7c-adduct**.

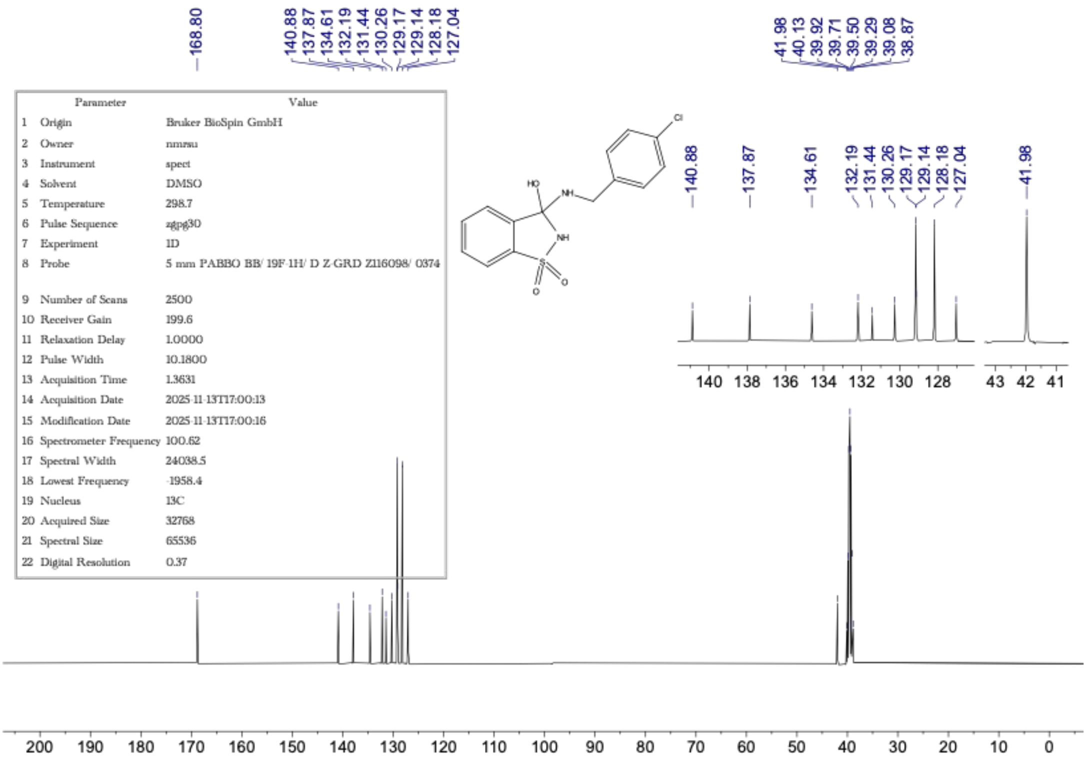

Figure S42. ^{13}C NMR spectrum (100 MHz, DMSO-d$_6$) of compound **7c-adduct**.

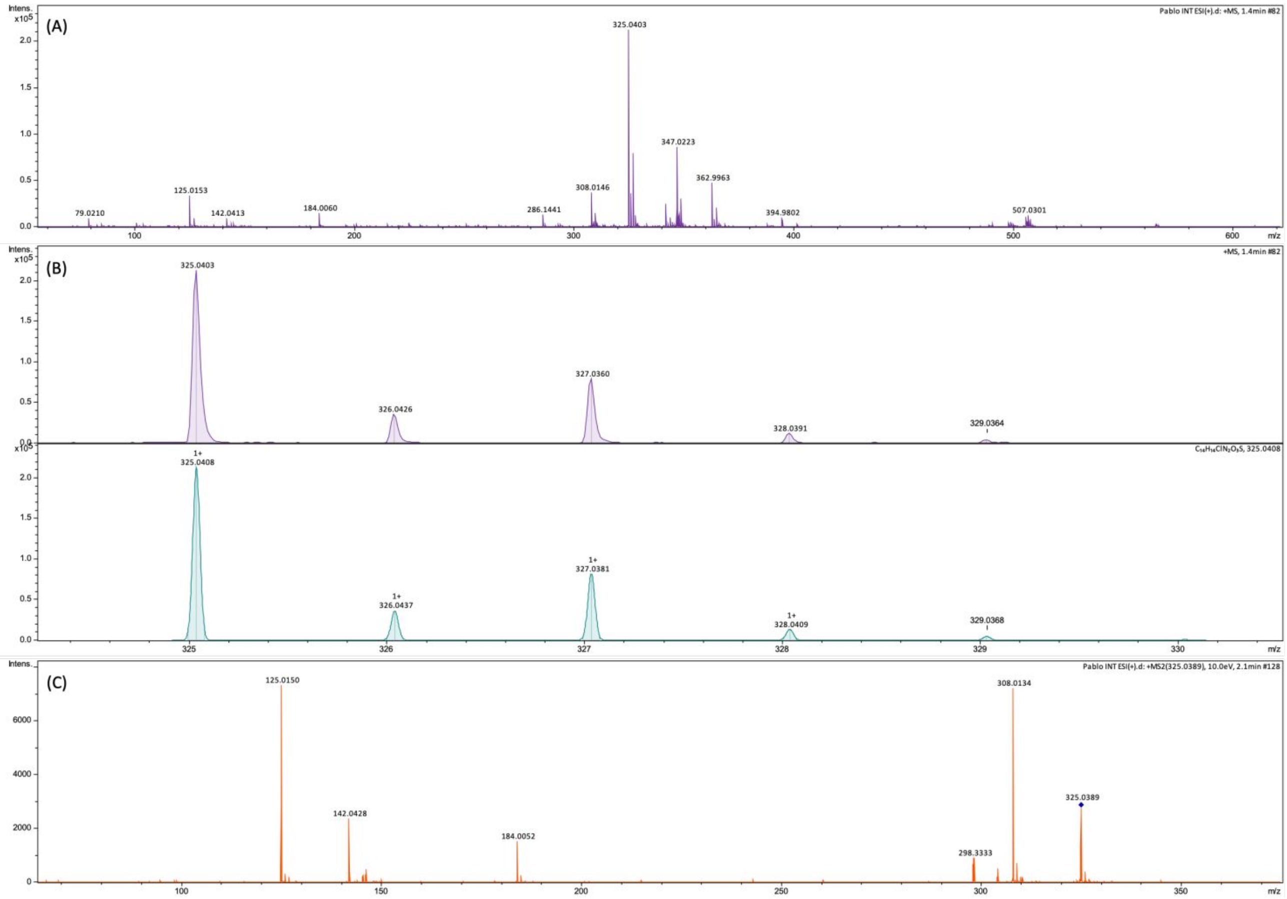

Figure S43. (A) ESI(+)-MS mass spectrum showing the $[M + H]^+$ ion at *m/z* 325.0403, consistent with the molecular formula $C_{14}H_{14}ClN_2O_3S$ assigned to compound **7c-adduct**. (B) Expanded view of the isotopic region of the molecular ion $[M + H]^+$ at *m/z* 325.0408 and comparison with the theoretical isotopic pattern for *m/z* 325.0403. (C) ESI(+)-MS/MS fragmentation spectrum (collision energy: 10.0 eV) obtained after selection of the precursor ion at *m/z* 325.0389, showing the main fragment ions of compound **7c-adduct**.

3.3. Single-crystal X-ray diffraction analysis of 3-(propylamino)benzo[*d*]isothiazole 1,1-dioxide (7j)

A colourless, plate-shaped crystal was mounted on the goniometer. Data for **7j** were collected from a single crystal in 16.61 hours at 100(2) K on a Bruker D8 QUEST Fixed Chi Diffractometer with a sealed tube using a curved graphite as monochromator and a Bruker PHOTON IV CPAD detector. The diffractometer was equipped with an Oxford Cryostream 1000 low temperature device and used Mo K_α radiation (λ = 0.71073 Å). All data were integrated with SAINT V8.42, yielding 29479 reflections of which 2574 where independent (average redundancy 11.45) and 64.8% were greater than 2σ(F^2).[8] A Multi-Scan absorption correction using SADABS 2016/2 was applied.[9] The structure was solved by intrinsic phasing methods with SHELXT 2018/2 and refined by full-matrix least-squares methods against F^2 using SHELXL-2019/2.[10,11] All non-hydrogen atoms were refined with anisotropic displacement parameters. All C-bound hydrogen atoms were refined with isotropic displacement parameters. Some of their coordinates were refined freely and some on calculated positions using a riding model with their U_{iso} values constrained to 1.5 times the U_{eq} of their pivot atoms for terminal sp^3 carbon atoms and 1.2 times for all other carbon atoms. Crystallographic data for the structures reported in this paper have been deposited with the Cambridge Crystallographic Data Centre.[12] CCDC 2535307 contain the supplementary crystallographic data for this paper. These data can be obtained free of charge from The Cambridge Crystallographic Data Centre via www.ccdc.cam.ac.uk/structures. This report and the CIF file were generated using FinalCif.[13]

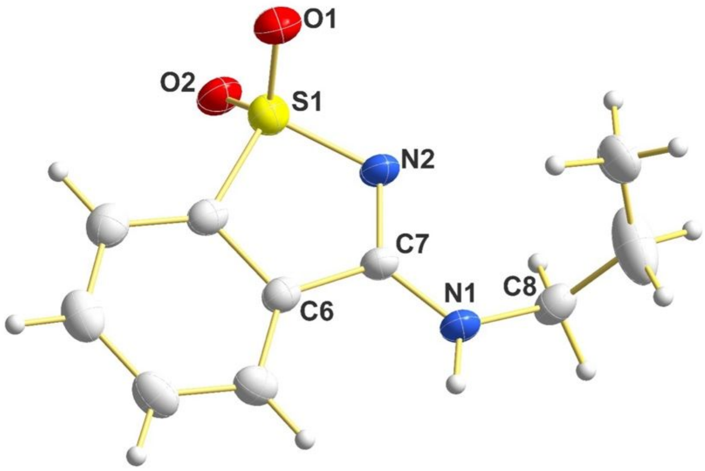

Figure S44. Molecular structure of 3-(propylamino)benzo[*d*]isothiazole 1,1-dioxide (**7j**), with the main atoms labeled. Thermal ellipsoids are drawn at the 50% probability level. The terminal methyl group is disordered over two positions; for clarity, only one of them is shown.

Table S1. Crystal data and structure refinement for 7j

CCDC number	2535307
Empirical formula	$C_{10}H_{12}N_2O_2S$
Formula weight	224.28
Temperature [K]	100(2)
Crystal system	triclinic
Space group (number)	$P\overline{1}$ (2)
a [Å]	7.0126(16)
b [Å]	7.1073(15)
c [Å]	10.401(2)
α [°]	95.338(8)
β [°]	92.959(9)
γ [°]	92.972(9)
Volume [Å³]	514.6(2)
Z	2
ρ_{calc} [gcm^{-3}]	1.447
μ [mm^{-1}]	0.295
F(000)	236
Crystal size [mm³]	0.032×0.197×0.24
Crystal colour	colourless
Crystal shape	plate
Radiation	Mo K_α (λ=0.71073 Å)
2θ range [°]	5.77 to 56.75 (0.75 Å)
Index ranges	−9 ≤ h ≤ 9<br>−9 ≤ k ≤ 9<br>−13 ≤ l ≤ 13
Reflections collected	29479
Independent reflections	2574<br>R_{int} = 0.1389<br>R_{sigma} = 0.0769
Completeness to θ = 25.242°	99.9
Data / Restraints / Parameters	2574 / 3 / 151
Goodness-of-fit on F^2	1.024
Final *R* indexes [I≥2σ(I)]	R_1 = 0.0720<br>wR_2 = 0.1878
Final *R* indexes [all data]	R_1 = 0.1152<br>wR_2 = 0.2182
Largest peak/hole [eÅ^{-3}]	1.07/−0.40

Table S2. Atomic coordinates and Ueq [Å²] for 7j

Atom	*x*	*y*	*z*	U_{eq}
S1	0.71968(13)	0.81360(11)	0.31791(9)	0.0298(3)
O1	0.8966(4)	0.9064(4)	0.2895(3)	0.0405(7)
O2	0.5599(4)	0.9334(3)	0.3245(3)	0.0353(6)
N1	0.6331(4)	0.3017(4)	0.2226(3)	0.0282(7)
H1N	0.645(6)	0.207(3)	0.268(3)	0.034
N2	0.6675(4)	0.6274(4)	0.2186(3)	0.0285(7)
C1	0.7483(5)	0.6967(5)	0.4593(3)	0.0277(7)
C2	0.7922(5)	0.7685(5)	0.5859(4)	0.0332(8)
H2	0.808928	0.900906	0.609687	0.040
C3	0.8108(5)	0.6386(6)	0.6768(4)	0.0377(9)
H3	0.841625	0.682624	0.764849	0.045
C4	0.7849(5)	0.4449(6)	0.6412(4)	0.0357(9)
H4	0.797816	0.359306	0.705634	0.043
C5	0.7408(5)	0.3744(5)	0.5139(4)	0.0306(8)
H5	0.723422	0.242045	0.490137	0.037
C6	0.7228(5)	0.5034(5)	0.4219(3)	0.0258(7)
C7	0.6729(5)	0.4725(4)	0.2807(3)	0.0254(7)
C8	0.5658(5)	0.2627(5)	0.0869(4)	0.0327(8)
H8A	0.488194	0.367516	0.062278	0.039
H8B	0.481761	0.145397	0.077050	0.039
C9	0.7244(7)	0.2403(9)	−0.0038(4)	0.0646(15)
H9A	0.776103	0.116071	0.009410	0.078
H9B	0.661730	0.226782	−0.091920	0.078
C10A	0.8762(12)	0.3595(11)	−0.0086(7)	0.044(2)
H10D	0.972557	0.296926	−0.059734	0.066
H10E	0.930400	0.397381	0.079255	0.066
H10F	0.837490	0.471775	−0.049006	0.066
C10B	0.8745(12)	0.1263(13)	0.0058(9)	0.037(2)
H10A	0.827708	−0.006492	−0.010901	0.056
H10B	0.936250	0.148063	0.092983	0.056
H10C	0.967220	0.155671	−0.057848	0.056

Table S3. Anisotropic displacement parameters (Å^2) for 7j. The anisotropic displacement factor exponent takes the form: $-2\pi^2[\,h^2(a^*)^2U_{11} + k^2(b^*)^2U_{22} + \ldots + 2hka^*b^*U_{12}\,]$

Atom	U_{11}	U_{22}	U_{33}	U_{23}	U_{13}	U_{12}
S1	0.0380(5)	0.0188(4)	0.0323(5)	0.0034(3)	0.0018(3)	−0.0022(3)
O1	0.0470(17)	0.0310(13)	0.0428(16)	0.0041(12)	0.0060(13)	−0.0101(12)
O2	0.0477(16)	0.0200(11)	0.0387(15)	0.0054(10)	0.0012(12)	0.0030(11)
N1	0.0376(16)	0.0175(13)	0.0301(16)	0.0058(11)	0.0030(12)	0.0013(11)
N2	0.0398(17)	0.0183(13)	0.0274(15)	0.0055(11)	0.0001(12)	−0.0013(12)
C1	0.0262(17)	0.0232(16)	0.0339(19)	0.0034(14)	0.0044(14)	0.0013(13)
C2	0.0285(18)	0.0340(19)	0.036(2)	0.0001(16)	0.0009(15)	0.0002(15)
C3	0.0292(19)	0.053(2)	0.031(2)	0.0006(17)	0.0006(15)	0.0038(17)
C4	0.034(2)	0.045(2)	0.0303(19)	0.0109(16)	0.0012(15)	0.0085(16)
C5	0.0303(18)	0.0297(18)	0.0337(19)	0.0085(15)	0.0035(14)	0.0072(14)
C6	0.0252(16)	0.0220(15)	0.0303(18)	0.0021(13)	0.0027(13)	0.0007(13)
C7	0.0304(17)	0.0192(15)	0.0270(17)	0.0045(12)	0.0027(13)	0.0006(12)
C8	0.037(2)	0.0235(16)	0.036(2)	0.0007(14)	−0.0044(15)	0.0000(14)
C9	0.044(3)	0.113(5)	0.034(2)	−0.013(3)	0.000(2)	0.011(3)
C10A	0.057(5)	0.047(4)	0.027(4)	0.005(3)	−0.001(3)	0.001(4)
C10B	0.029(4)	0.044(5)	0.038(5)	−0.003(4)	0.000(3)	0.009(4)

Table S4. Bond lengths for 7j

Atom–Atom	Length [Å]
S1–O1	1.432(3)
S1–O2	1.443(3)
S1–N2	1.615(3)
S1–C1	1.762(4)
N1–C7	1.314(4)
N1–C8	1.462(5)
N1–H1N	0.865(17)
N2–C7	1.329(4)
C1–C2	1.381(5)
C1–C6	1.393(4)
C2–C3	1.388(5)
C2–H2	0.9500
C3–C4	1.392(6)
C3–H3	0.9500
C4–C5	1.386(5)
C4–H4	0.9500
C5–C6	1.391(5)
C5–H5	0.9500
C6–C7	1.485(5)
C8–C9	1.500(6)
C8–H8A	0.9900
C8–H8B	0.9900
C9–C10A	1.332(9)
C9–C10B	1.366(9)
C9–H9A	0.9900
C9–H9B	0.9900
C10A–H10D	0.9800
C10A–H10E	0.9800
C10A–H10F	0.9800
C10B–H10A	0.9800
C10B–H10B	0.9800
C10B–H10C	0.9800

Table S5. Bond angles for 7j

Atom–Atom–Atom	Angle [°]
O1–S1–O2	114.86(15)
O1–S1–N2	111.20(16)
O2–S1–N2	110.48(16)
O1–S1–C1	110.65(16)
O2–S1–C1	111.11(16)
N2–S1–C1	97.18(15)
C7–N1–C8	124.1(3)
C7–N1–H1N	118(2)
C8–N1–H1N	118(2)
C7–N2–S1	110.3(2)
C2–C1–C6	122.6(3)
C2–C1–S1	130.4(3)
C6–C1–S1	107.0(3)
C1–C2–C3	117.0(3)
C1–C2–H2	121.5
C3–C2–H2	121.5
C2–C3–C4	121.1(4)
C2–C3–H3	119.4
C4–C3–H3	119.4
C5–C4–C3	121.4(4)
C5–C4–H4	119.3
C3–C4–H4	119.3
C4–C5–C6	117.9(3)
C4–C5–H5	121.0
C6–C5–H5	121.0
C5–C6–C1	119.9(3)
C5–C6–C7	130.5(3)
C1–C6–C7	109.5(3)
N1–C7–N2	122.7(3)
N1–C7–C6	121.4(3)
N2–C7–C6	115.9(3)
N1–C8–C9	113.6(3)
N1–C8–H8A	108.9
C9–C8–H8A	108.9
N1–C8–H8B	108.9
C9–C8–H8B	108.9
H8A–C8–H8B	107.7
C10A–C9–C8	126.4(6)
C10B–C9–C8	127.1(6)
C10A–C9–H9A	105.7
C8–C9–H9A	105.7

C10A–C9–H9B	105.7
C8–C9–H9B	105.7
H9A–C9–H9B	106.2
C9–C10A–H10D	109.5
C9–C10A–H10E	109.5
H10D–C10A–H10E	109.5
C9–C10A–H10F	109.5
H10D–C10A–H10F	109.5
H10E–C10A–H10F	109.5
C9–C10B–H10A	109.5
C9–C10B–H10B	109.5
H10A–C10B–H10B	109.5
C9–C10B–H10C	109.5
H10A–C10B–H10C	109.5
H10B–C10B–H10C	109.5

Table S6. Torsion angles for 7j

Atom–Atom–Atom–Atom	Torsion Angle [°]
O1–S1–N2–C7	113.8(3)
O2–S1–N2–C7	−117.4(3)
C1–S1–N2–C7	−1.7(3)
O1–S1–C1–C2	65.2(4)
O2–S1–C1–C2	−63.7(4)
N2–S1–C1–C2	−178.9(3)
O1–S1–C1–C6	−113.3(3)
O2–S1–C1–C6	117.9(2)
N2–S1–C1–C6	2.6(3)
C6–C1–C2–C3	0.1(5)
S1–C1–C2–C3	−178.1(3)
C1–C2–C3–C4	−0.4(5)
C2–C3–C4–C5	0.4(6)
C3–C4–C5–C6	0.0(5)
C4–C5–C6–C1	−0.2(5)
C4–C5–C6–C7	−178.4(3)
C2–C1–C6–C5	0.2(5)
S1–C1–C6–C5	178.8(3)
C2–C1–C6–C7	178.7(3)
S1–C1–C6–C7	−2.7(3)
C8–N1–C7–N2	−4.5(5)
C8–N1–C7–C6	174.1(3)
S1–N2–C7–N1	178.9(3)
S1–N2–C7–C6	0.3(4)
C5–C6–C7–N1	1.4(6)
C1–C6–C7–N1	−176.9(3)
C5–C6–C7–N2	−179.9(3)
C1–C6–C7–N2	1.7(4)
C7–N1–C8–C9	89.4(5)
N1–C8–C9–C10A	−51.0(8)
N1–C8–C9–C10B	50.7(8)

Table S7. Hydrogen bonds for 7j

D–H···A [Å]	d(D–H) [Å]	d(H···A) [Å]	d(D···A) [Å]	<(DHA) [°]
C2–H2···O1#1	0.95	2.54	3.226(4)	129.2

Symmetry transformations used to generate equivalent atoms:
#1: 2-X, 2-Y, 1-Z;

4. REFERENCES

(1) Vorbrüggen, H.; Krolikiewicz, K. Amination, III. Trimethylsilanol as Leaving Group, V. Silylation—Amination of Hydroxy N-Heterocycles. *Chem. Ber.* **1984**, *117* (4), 1523–1541. https://doi.org/10.1002/cber.19841170421.

(2) Arraché Gonçalves, G.; Castro Do Nascimento, F.; Moura E Silva, S.; Valim Bizarro, C.; Augusto Basso, L.; Machado, P. Synthesis of N-Phenethylquinazolin-4-Amines via Silylation-Amination Mediated by Hexamethyldisilazane. *Results in Chemistry* **2022**, *4*, 100539. https://doi.org/10.1016/j.rechem.2022.100539.

(3) Eresko, A.; Tolkunov, V.; Tolkunov, S. Simple and Efficient Preparation of 3-Substituted 6-(4-Chlorophenyl)-9-Methyl-12H-[1]Benzofuro[3,2-e][1,2,4]Triazolo[4,3-b][1,2]Diazepines via a Silylation–Amination Reaction. *Monatsh Chem* **2011**, *142* (9), 931–934. https://doi.org/10.1007/s00706-011-0500-z.

(4) Vorbrueggen, H. Adventures in Silicon-Organic Chemistry. *Acc. Chem. Res.* **1995**, *28* (12), 509–520. https://doi.org/10.1021/ar00060a007.

(5) Vorbrüggen, H.; Krolikiewicz, K.; Niedballa, U. SYNTHESIS OF NUCLEOSIDES WITH USE OF TRIMETHYLSILYL-HETEROCYCLES. *Annals of the New York Academy of Sciences* **1975**, *255* (1), 82–90. https://doi.org/10.1111/j.1749-6632.1975.tb29215.x.

(6) Jang, M.; De Jonghe, S.; Gao, L.; Rozenski, J.; Herdewijn, P. Development of Synthetic Strategies for the Construction of Pyrido[4,3- *d*]Pyrimidine Libraries – the Discovery of a New Class of PDE-4 Inhibitors. *Eur J Org Chem* **2006**, *2006* (18), 4257–4269. https://doi.org/10.1002/ejoc.200600339.

(7) De Jonghe, S.; Marchand, A.; Gao, L.-J.; Calleja, A.; Cuveliers, E.; Sienaert, I.; Herman, J.; Clydesdale, G.; Sefrioui, H.; Lin, Y.; Pfleiderer, W.; Waer, M.; Herdewijn, P. Synthesis and in Vitro Evaluation of 2-Amino-4-N-Piperazinyl-6-(3,4-Dimethoxyphenyl)-Pteridines as Dual Immunosuppressive and Anti-Inflammatory Agents. *Bioorganic & Medicinal Chemistry Letters* **2011**, *21* (1), 145–149. https://doi.org/10.1016/j.bmcl.2010.11.053.

(8) SAINT, 2025.

(9) Krause, L.; Herbst-Irmer, R.; Sheldrick, G. M.; Stalke, D. Comparison of Silver and Molybdenum Microfocus X-Ray Sources for Single-Crystal Structure Determination. *J Appl Crystallogr* **2015**, *48* (1), 3–10. https://doi.org/10.1107/S1600576714022985.

(10) Sheldrick, G. M. *SHELXT* – Integrated Space-Group and Crystal-Structure Determination. *Acta Crystallogr A Found Adv* **2015**, *71* (1), 3–8. https://doi.org/10.1107/S2053273314026370.

(11) Sheldrick, G. M. Crystal Structure Refinement with *SHELXL*. *Acta Crystallogr C Struct Chem* **2015**, *71* (1), 3–8. https://doi.org/10.1107/S2053229614024218.

(12) Groom, C. R.; Bruno, I. J.; Lightfoot, M. P.; Ward, S. C. The Cambridge Structural Database. *Acta Crystallogr B Struct Sci Cryst Eng Mater* **2016**, *72* (2), 171–179. https://doi.org/10.1107/S2052520616003954.

(13) *D. Kratzert, FinalCif, (Bruker Edition)*. https://dkratzert.de/finalcif.html.
